# A Comprehensive Summary of the Knowledge on COVID-19 Treatment

**DOI:** 10.14336/AD.2020.1124

**Published:** 2021-02-01

**Authors:** Yu Peng, Hongxun Tao, Senthil Kumaran Satyanarayanan, Kunlin Jin, Huanxing Su

**Affiliations:** ^1^State Key Laboratory of Quality Research in Chinese Medicine, Institute of Chinese Medical Sciences, University of Macau, Macao, China.; ^2^Department of Pharmacology and Neuroscience, University of North Texas Health Science Center, Fort Worth, TX 76107, USA.

**Keywords:** COVID-19, treatment, Chinese Medicine, strategies

## Abstract

Currently, the world is challenged by the coronavirus disease 2019 (COVID-19) pandemic. Epidemiologists and researchers worldwide are invariably trying to understand and combat this precarious new disease. Scrutinizing available drug options and developing potential new drugs are urgent needs to subdue this pandemic. Several intervention strategies are being considered and handled worldwide with limited success, and many drug candidates are yet in the trial phase. Despite these limitations, the development of COVID-19 treatment strategies has been accelerated to improve the clinical outcome of patients with COVID-19, and some countries have efficiently kept it under control. Recently, the use of natural and traditional medicine has also set the trend in coronavirus treatment. This review aimed to discuss the prevailing COVID-19 treatment strategies available globally by examining their efficacy, potential mechanisms, limitations, and challenges in predicting a future potential treatment candidate and bridging them with the effective traditional Chinese medicine (TCM). The findings might enrich the knowledge on traditional alternative medication and its complementary role with Western medicine in managing the COVID-19 epidemic.

## 1. Introduction

Since December 2019, the new coronavirus disease (COVID-19) has become a major global epidemic threat. On January 30, 2020, the World Health Organization (WHO) announced COVID-19 as a public health emergency of international concern (PHEIC) [1]. Up to November 23, 2020, 86,464 confirmed cases, 81,508 (94.27%) cured cases, and 4634 (5.36%) deaths were reported in mainland China (www.nhc.gov.cn/xcs/yqtb/list_gzbd.shtml). China has efficiently controlled this epidemic crisis. However, the global scenario seems out of control with the death toll more than one million, and more than 57 million people have been diagnosed with the viral infection (www.who.int/emergencies/diseases/novel-coronavirus-2019/situation-reports).

COVID-19 is caused by a new coronavirus severe acute respiratory syndrome coronavirus 2 (SARS-CoV-2), which belongs to the β-coronavirus group and is highly pathogenic to humans. SARS-CoV-2 is the third coronavirus strain discovered so far that can cause zoonotic diseases. The other two strains are SARS-CoV and Middle East respiratory syndrome (MERS) coronavirus (MERS-CoV). COVID-19 is the third global plague caused by coronavirus in the last 20 years. During 2002-2003, bat-originated SARS-CoV, which could be transmitted to humans through the intermediary host palm civet cat, infected 8422 people in China and Hong Kong. SARS-CoV resulted in 916 deaths and increased the cumulative mortality rate to 11% [2]. Ten years later, another coronavirus named MERS-CoV swept Saudi Arabia, which also originated from bats, but was transmitted to humans through its intermediate host camel. MERS-CoV infected 2494 people, caused 858 deaths, and increased the cumulative mortality rate to 34% [3].

Phylogenetic analysis has shown that SARS-CoV-2 belongs to the Sarbecovirus subtype of β-coronavirus, which is similar to the other two bat-derived severe acute respiratory syndrome (SARS)-like coronaviruses collected in 2018, named bat-SL-CoVZC45 and bat-SL-CoVZXC21 (88% similarity), but far away from SARS-CoV (about 79% similarity) and MERS-CoV (about 50% similarity) [4]. A recent report has revealed that the β-coronavirus found in Malayan pangolins (*Manis javanica*) is a close relative of SARS-CoV-2 with a high degree of identity (97%) in the receptor-binding domain (RBD) sequence [5]. The homology modeling has revealed that SARS-CoV-2 shares a similar RBD structure to SARS-CoV, indicating similar pathogenesis between these two diseases [4]. S protein of coronavirus can bind to angiotensin-converting enzyme 2 (ACE2) of the host cell to promote the entry of the virus into the target cell [6], while the tissue distribution of the host receptor is usually consistent with the tropisms of the virus [7]. ACE2 is mainly distributed in human respiratory epithelial cells, lung parenchyma, vascular endothelium, kidney cells, and small intestine cells [8, 9]. Mutation and recombination are the most important mechanisms in the evolution of RNA viruses and also the main reason for the genetic diversity of SARS-CoV-2. Nine putative recombination patterns have been identified in the SARS-CoV-2 genome, including six key recombination regions in the S gene, RNA-dependent RNA polymerase (RdRp), nsp13, and ORF3a. [10] The number and incidence of mutations are much higher in Europe and North America compared with Asia, which also indicates different mutation patterns. [11] A high-throughput and high-coverage SARS-CoV-2 complete genome sequence study has observed that distinct time-course evolution patterns are divided into four main mutation groups. [12] Two nonsynonymous mutations A23403G and C14408T are mainly from Europe and North America, one nonsynonymous mutation T28144C is mainly distributed in Asia and Spain, two nonsynonymous mutations G11083T and G26144T are mainly distributed in Asia and some European countries, while two other nonsynonymous mutations G1440A and G2891A are mainly found in Europe. However, some virus strains may be eliminated with the control of the disease and the passage of time. At present, the S protein D614G mutant strain (A23403G) has become the most common form of a global pandemic. G614 mutation is related to higher infectivity. The quantitative analysis has revealed that the infectivity titer of virus particles with D614G mutation increases by 2.6-9.3 times [13]. Drug screening should pay attention to the efficiency on different SARS-CoV-2 lineages or clades or subtypes to speed up drug development.

SARS-CoV-2- and SARS-CoV-infected patients have similar symptoms of lower respiratory tract infection and gastrointestinal manifestations; four amino acid variations of S protein exist between SARS-CoV-2 and SARS-CoV [14, 15]. Animal experiments have proved that SARS-CoV-2 can effectively replicate in respiratory epithelial cells of the entire respiratory tract tissues (including nasal cavity, bronchi, bronchioles, and alveoli) [16]. The symptoms of SARS-CoV-2 infection are mainly lower respiratory tract infections, such as fever and dry cough. Meanwhile, some patients have nasal congestion and runny nose; some patients might suffer from fatigue and myalgia; while a small number of patients also show digestive tract reactions, such as vomiting and diarrhea [17-20]. In severe cases, dyspnea and/or hypoxemia usually occur within a week of onset. More severe cases quickly progress to acute respiratory distress syndrome, septic shock, diffuse coagulation, and multiple-organ failure. SARS-CoV-2 has three main routes of person-to-person transmission: respiratory droplets, such as coughing, sneezing, and droplet inhalation; contact transmission, such as contact with the oral cavity, nasal cavity, and eye mucosa [21]; and aerosol transmission, for which experiments have shown that SARS-CoV-2 can remain viable in aerosols for 3 h [22], and virus aerosolization in a confined space may be contagious [23]. Patients with COVID-19 are most infectious before the onset of symptoms, while SARS-CoV-2 begins to shed 2-3 days before the first wave of symptoms appears. After symptoms appear, the viral load in the patient's body decreases faster [24]. A report of a SARS-CoV-2 infection in Germany indicates that the spread of the virus may also occur through contact with asymptomatic patients [25]. In a new study of an asymptomatic cynomolgus monkey model, SARS-CoV-2 virus shedding peaked in the early stage (4 days) of the infection process, similar to the situation of asymptomatic patients [16]. However, no reliable evidence has shown the efficiency of fecal-oral transmission route and vertical transmission risk yet, which seems relatively low temporarily, but still needs further research and confirmation [26, 27].

Data on pathology, computerized tomography scan (CT) imaging, clinical characteristics, or complications have shown that both MERS and SARS are more severe than COVID-19 [28-49] (Table 1). The mortality rate of MERS-CoV infection is more than three and six times of SARS-CoV and SARS-CoV-2, respectively. However, MERS-CoV is not easy to be transmitted from person to person through close contact with infected patients; it is primarily through droplets of infected persons [50]. Despite the low case mortality rate, the number of deaths caused by COVID-19 has already exceeded the sum of SARS and MERS [51]. Taken together, it indicates that the differences in the epidemiological characteristics of these viruses can be caused by other factors, including high viral load of the upper respiratory tract, possibility of asymptomatic distribution and transmission of the virus in people infected with SARS-CoV-2, as well as other related social aspects. It has been found that the SARS-CoV RNA viral load reaches its peak 7-10 days after the onset of SARS symptoms, while the peak of SARS-CoV-2 RNA is within 5 days after the onset of symptoms [52]. The Diagnosis and Treatment Protocol for Novel Coronavirus Pneumonia (Trial Version 8) states that the incubation period is infectious, and highly infectious within 5 days after the onset [49]. In addition, SARS-CoV-2 begins to shed 2-3 days before the onset of symptoms [24]. When the patient’s symptoms are still mild and the symptoms of upper respiratory tract infection have just appeared, the new coronavirus can spread more effectively than the SARS virus through active pharynx virus shedding activities. The intensive care unit (ICU) air samples and low-altitude samples of Wuhan Fangcang Hospital have been tested positive for the virus, indicating that the deposition and resuspension of virus aerosols on protective clothing or floor surfaces are potential transmission routes [53]. Meanwhile, exposure to virus-contaminated objects can also cause infection [49]. The airborne SARS-CoV-2 concentration increases significantly with the increase in the flow of people. The gathering of asymptomatic carriers may be a potential source of airborne SARS-CoV-2 [53]. Experiments have also shown that the stability of SARS-CoV-2 in aerosols and on metal surface is similar to that of SARS-CoV [22]. Currently, no specific and effective clinical treatment exists for these three diseases. The official clinical management strategies of SARS [46, 47], MERS [48], and COVID-19 [49] are summarized in Table 1, which are mainly based on supportive treatments focusing on relieving symptoms and preventing or treating complications. The specific treatment should depend on clinical manifestations and patient factors (for example, age and whether they have comorbidities). Meanwhile, many drug candidates have shown good therapeutic prospects and may be valuable in the future.

**Table 1:** Pathogenetic, epidemiological and clinical characteristics of SARS-CoV-2, SARS-CoV and MERS-CoV.

	SARS-Cov	MERS-Cov	SARS-Cov-2
Genus	Beta-CoVs lineage B	Beta-CoVs lineage C	Beta-CoVs lineage B
Date/Place first detected	November 2002, Guangdong China	June 2012, Jeddah, Saudi Arabla	December 2020, Wuhan China
Possible nature reservoir	Bat	Bat	Bat
Possible intermediate host	Palm civets	Camel	Pangolin
Virus transmission	1. Respiratory droplets 2. Contact 3. Aerosol [28]	1. Respiratory droplets 2. Contact [29]	1. Respiratory droplets 2. Contact 3. Aerosol [30]
Predominant cellular receptor [31]	ACE2	DDP4	ACE2
Receptor distribution [31]	Arterial and venous endothelium; arterial smooth muscle; small intestine, respiratory tract epithelium; alveolar monocytes and macrophages	Respiratory tract epithelium; kidney, small intestine; liver and prostate; activated leukocytes	Arterial and venous endothelium; arterial smooth muscle; small intestine, respiratory tract epithelium; alveolar monocytes and macrophages
Number of affected countrie and area	29	27	183
Confirmed cases	8096	2494	571678
Death cases	744	858	26494
Mortality rate	9.19%	34.40%	4.63%
Severity Rate	10-20% [28]	--	7-21% [17, 18, 32]
Incubation Period	4d (2-10d)[33]	5.2d (2-14d)[34]	5.2d (2-14d)[35]
Epidemic doubling time[36]	4.6-14.2	90	6.4
Reproductive number, R0[36, 37]	1.4-5.5	<1	2.2-3.6
Ventilation support	13-26% [38]	85.2% [39]	4.2% [32]
ICU admission	19-34% [38]	53-89% [39]	10% [40]
invasive mechanical ventilation	17% [31]	37% [31]	7-9.6% [17, 18]
Symptom	Fever (99%); Headache (39%); Myalgia (59%); Cough(58%); Shortness of breath (27%); Sore throat (17%); Nausea/vomiting (15%); Diarrhoea (17%)[31]	Fever (84%); Headache (19%); Myalgia (98%); Cough(63%); Shortness of breath 35%); Sore throat (13%); Nausea/vomiting (15%); Diarrhoea (20%)[31]	Fever (83%) Cough (82%) Shortness of breath (31%) Muscle ache (11%) Confusion (9%) Headache (8%) Sore throat (5%) Rthinorrhoea (4%) [18]
Pathology	Edematous lung, bronchial epithelial denudation, loss of cilia, squamous metaplasia fibrosis [41].	Exudative diffuse alveolar damage with hyaline membranes, pulmonary edema, type II pneumocyte hyperplasia, interstitial pneumonia [41].	Inflammation, mucus and fibrosis [18].
CT imaging	1. Air-space opacities; 2. ~50% unilateral multifocal or bilateral involvement [31].	1. Ground glass opacities and consolidation; 2. Higher rate of Pleural effusion and pneumothorax [31].	1. Small patches and interstitial changes; 2. Ground glass opacity; 3. Rare Pleural effusion [17].
Clinical characters	1. Hypoalbuminemia; 2. Thrombocytopenia; 3. Leukopenia; 4. Lymphopenia.[28, 42]	1. Increase White Blood Cells count; 2. Decrease lymphocytes count; 3. Decrease platelets count; 4. Decrease Red Blood Cells count [43].	1. The total number of peripheral blood leukocytes was normal or decreased; 2. Decreased lymphocyte count; 3. Increased CRP and erythrocyte sedimentation rate [44].
Complication	1. Acute kidney injury (AKI) is a significant characteristic of SARS patients [38].	1. Acute kidney injury (AKI) is a significant characteristic of MERS patients; 2. Vasopressor therapy was much more common in MERS, (81%)[31].	1. Acute respiratory distress syndrome; 2. RNA aemia, acute cardiac injury; 3. Secondary (super-)infections [41].
Affected organ	Respiratory tract; kidney; liver [38]	Respiratory tract; intestinal tract; genitourinary tract; liver, kidney, neurons; monocyte; T lymphocyte; Cardiovascular [31].	Respiratory tract; intestinal tract; liver; kidney [32].
Prognostic factor	1. Age 2. Underlying condition 3. Male 4. LDH level 5. Neutrophil count 6. CD4 7. CD8 [38]	1. Age 2. Underlying condition 3. Male [45]	1. Age 2. Underlying condition 3. Male 4. Lymphocyte count 5. Lactic acid 6. IL-6 and CRP [18]
Clinical management	-Continuous nasal cannula oxygen is given early (the oxygen concentration is generally 1~3 L/min); -Given oseltamivir within 48 hours of onset can help reduce the symptoms. -Fever>38.5, or obvious body aches, can use antipyretic analgesics; Those with high fever should be given physical cooling measures; Salicylic acid antipyretic analgesics are forbidden for children. -Cough and expectorants can be given antitussive and expectorant drugs; -With damage to organs such as heart, liver, kidney, etc. should be treated accordingly. -With diarrhea should be paid attention to rehydration and correct water and electrolyte imbalances; -Use of glucocorticoids, the recommended adult dose is equivalent to 80-320 mg/d of methylprednisolone, reduced or stopped when the clinical manifestations have been improved or the chest radiograph shown the absorbed shadows in lung; -Antiviral therapy has not yet found specific drugs; Possible to try protease inhibitors such as lopinavir and ritonavir; -When the diagnosis is unclear, new quinolones or β-lactams combined with macrolides can be used for trial treatment; -The pathogens of secondary infections include gram-negative bacilli, drug-resistant gram-positive cocci, fungi, and Mycobacterium tuberculosis, and appropriate antibacterial drugs should be selected accordingly; -Chinese medicine as an alternative strategy. [46, 47]	For patients with pneumonia or comorbidities: -With signs of severe respiratory distress, shock or hypoxemia, oxygen therapy should be started immediately. -It is recommended that fluid management in patients is necessary, while provided that there is no sign of shock. -Empirical antibacterial treatment (including antibiotics and antiviral drugs) should be initiated for hospitalized patients with suspected MERS pneumonia; If sepsis is suspected, it should be started within one hour; -Antipyretic/analgesic is recommended to control fever and pain. -Corticosteroids are generally not recommended; however, stress doses can be given when needed. -Patients who are about to develop or have developed respiratory failure should be admitted to the ICU ward. For patients without pneumonia or comorbidities: -Supportive treatment is recommended, including the use of antipyretics and analgesics (such as acetaminophen, ibuprofen) to relieve pain and fever; -Patients should stay hydrated, but should not consume too much fluid, as this may worsen oxygenation [48].	-General treatment: strengthen supportive treatment, ensure adequate energy intake; pay attention to water and electrolyte balance, maintain internal environment stability; give effective oxygen therapy measures in time, including nasal cannula, mask oxygen and nasal high flow oxygen therapy; avoid blind or inappropriate use of antibacterial drugs, especially the combined use of broad-spectrum antibacterial drugs. -Antiviral treatment: drugs with potential antiviral effects (such as α-interferon, ribavirin, chloroquine, and arbidol) should be used early, and recommended to be applied to patients with severe high-risk factors and severely ill tendencies. -Immunotherapy: convalescent plasma from recovered patients, intravenous injection of COVID-19 human immunoglobulin, tocilizumab; -Glucocorticoid treatment can be applied for patients with progressive deterioration of oxygenation indicators, rapid imaging progress, and excessive activation of the body's inflammatory response for a short period of time (equivalent to methylprednisolone 0.5~1mg/kg/day, 3 to 5 days); -For severe and critical cases: On the basis of the above treatment, actively prevent and treat complications, treat basic diseases, prevent secondary infections, and provide organ function support in time; -Chinese medicine as an alternative strategy [49].

Accumulating recent pieces of evidence have unraveled the understanding of the development and direction of the epidemic. The National Health Commission of China has issued and implemented a series of guidelines based on the epidemiological evidence of pathogens responsible for COVID-19 infection, as well as epidemiological characteristics, clinical characteristics, prevention, diagnosis, staging, treatment, traditional Chinese medicine (TCM) syndrome differentiation treatment, and rehabilitation management [49, 54, 55]. Since the beginning of the pandemic, clinicians and epidemiologists around the world have been exploring the most effective treatment strategy to greatly benefit those who are affected by potentially life-threatening complications of COVID 19, as well as to further subdue this pandemic.

## 2. Current Therapeutics and Drug Development

According to clinical syndromes, patients with COVID-19 can be divided into four categories: (1) mild cases, presenting mild fever and mild fatigue, but no symptom of pneumonia on imaging; (2) moderate cases, with fever, respiratory tract and other symptoms, and manifestations of pneumonia on imaging; (3) severe cases, presenting shortness of breath, respiratory rate ≥30 times/min, resting oxygen saturation ≤93%, arterial oxygen partial pressure (PaO_2_)/oxygen inhalation concentration (FiO_2_) ≤300 mmHg (1 mm Hg = 0.133 kPa); and (4) critical cases, with shortness of breath and the need for mechanical ventilation, or shock, or combined with other organ failure, which requires ICU care [49]. For mild and moderate cases, the main clinical measures are to implement close observation of vital signs, blood oxygen saturation, and electrolyte balance; ensure adequate sleep; as well as strengthen nutrition. Oxygen therapy and antiviral therapy should be given in time according to real-time conditions such as blood routine, biochemical indicators, blood gas analysis, and chest imaging. For severe and critical cases, the number of peripheral blood lymphocytes progressively decreases, along with the progressive increase in the expression of peripheral blood inflammatory factors such as interleukin 6 (IL-6) and C-reactive protein (CRP), as well as the progressive elevation of lactic acid. A rapid progression of intrapulmonary lesions in the short term is a clinical warning indicator for severe and critical cases. Besides symptomatic treatment, these patients should also be paid great attention to prevent complications and secondary infections and provide organ function support promptly. These patients usually require noninvasive ventilation or high-flow nasal catheter oxygen therapy. When the airway platform pressure is less than 30 cm H_2_O, mechanical ventilation should be provided. Closed sputum aspiration and bronchoscopy are performed when necessary along with the other appropriate treatments to act on the airway secretions. Extracorporeal membrane oxygenation (ECMO) is also considered in severe cases without contraindications [49]. However, for severe and critically affected patients, circulatory support, renal replacement therapy, and solutions to reverse cytokine storms (CS) should also be considered and administered.

**Figure 1.**
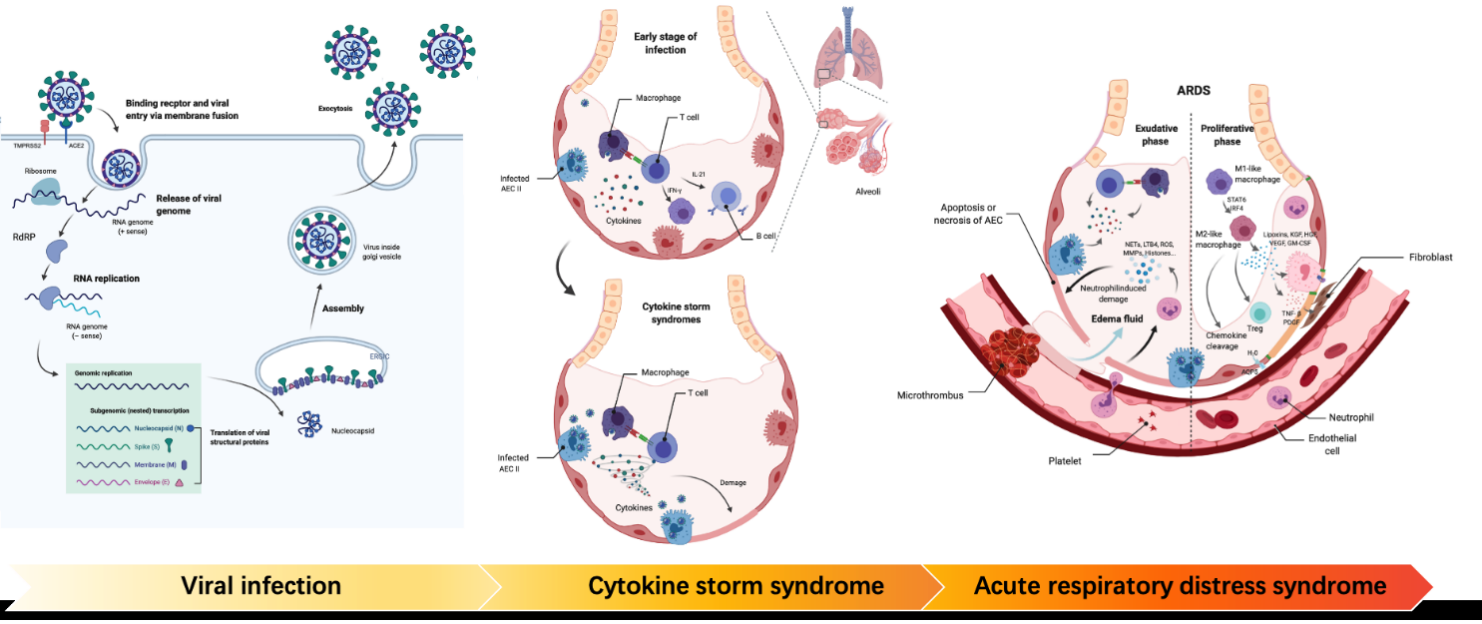
The development of COVID19. Viral infection: TMPRSS2 cleaves the S protein of SARS-CoV-2; The RBD on the S1 subunit binds to ACE2 on the cell surface. Following entry into the cell, viral RNA is released and combined with RdRp to synthesize a full-length negative-strand RNA as an RNA replication template. After translation, structural proteins are localized to the inner membrane of Golgi for assembly. Cytokine storm syndrome: The immune system is over-activated, followed by the overproduction of multiple inflammatory factors. Multiple immune cells are recruited. As a result, healthy cells are damaged by overactive immune response. Acute respiratory distress syndrome: In the exudative phase, macrophages are activated and release pro-inflammatory mediators, which leads to the aggregation of neutrophils and monocytes. Activated neutrophils induce further damage. The injury leads to loss of barrier function and fluid accumulation in the interstitium and alveoli. In the proliferative period, the tissue homeostasis is recovered.

As mentioned earlier, SARS-CoV-2 can infect the human body by binding to host cell receptors, thereby causing body damage. As shown in Figure 1, in severe cases, it may be associated with the induction of excessive immune response, which is related to "self-attack" followed by multiple-organ damage and CS [56]. At present, the research and development of drugs mainly focus on three aspects: antiviral drugs, suppression of excessive immune response, and vaccination.

### 2.1 Antiviral treatment

Currently, the Food and Drug Administration (FDA) has authorized the emergency use of remdesivir to treat COVID-19. China has gained a lot of experience in the treatment process and developed “Diagnosis and Treatment Protocol for COVID-19 (Trial Version 8).” The guideline has recommended the use of α-interferon, ribavirin combined with α-interferon or lopinavir/ ritonavir, chloroquine phosphate, and arbidol. However, no specific antiviral treatment for COVID-19 still exists. Therefore, the development of antiviral drugs is imminent. The main antigen component S protein (spike protein) on SARS-CoV-2 plays an important role during the viral infection process. The S protein comprises two subunits, S1 and S2, which can be cleaved by the transmembrane protease Serine 2 (TMPRSS2). The RBD on the S1 subunit binds to ACE2 on the host cell surface [57] to promote the virus's entry into the host cell through endocytosis [58]. After entering the cell, the viral RNA is released. Then, two open reading frames and protein transcriptase [RNA-dependent RNA polymerase (RdRp)] form a viral transcriptase complex [59]. Subsequently, a full-length negative-strand RNA is synthesized from the viral RNA (positive strand) as a full-length genomic RNA replication template [60]. After translation, structural proteins are localized to the inner membrane of the Golgi for assembly [61]. According to the mechanism of viral infection, blocking virus entry, inhibiting the process of viral replication and assembly, and removing the virus are the key strategies to the development of antiviral drugs.

#### 2.1.1 Blocking the virus from entering the host cell

ACE2 is the host cell binding site for viral infection. At the beginning of the disease outbreak, it is believed that the use of inhibitors of the renin-angiotensin-aldosterone system (RAAS) may increase the expression level of ACE2, thereby making individuals susceptible to severe COVID-19 [62]. A large case study by Francisco et al. [63] has confirmed that the application of RAAS inhibitors does not increase the proportion of COVID-19 admissions, nor does it increase ICU admissions and mortality after adjusting for age, sex, cardiovascular comorbidities, and risk factors, suggesting that these inhibitors need not be banned and can still be used as appropriate.

TMPRSS2-cracked virus S protein plays an important role in initiating the SARS-CoV-2 virus infection process. Camostat mesilate is an effective TMPRSS2 inhibitor. Preclinical evidence has shown that camostat mesilate can prevent the SARS-CoV-2 virus from infecting 293T cell lines [57] and reduce the mortality of SARS-CoV-infected mice [64]. Compared with camostat mesilate, its analogue nafamostat mesilate has a stronger activity to block the entry of the SARS-CoV-2 virus in *in vitro* tests [65]. Theoretically, imatinib can also have a type II transmembrane serine protease inhibitory effect [66, 67], while α-1antitrypsin, as a TMPRSS2 inhibitor, can also exhibit the ability to block the virus from entering host cells [68]. These TMPRSS2 inhibitors have all entered the clinical phase. Interestingly, androgens can mediate the upregulation of TMPRSS2 mRNA [69], which may be related to the difference in the proportion of men and women infected with the SARS-CoV-2 virus. A large observational study has revealed that patients with prostate cancer receiving androgen-deprivation therapies (ADTs) are partially protected from infection [70]. ADT therapeutic drugs such as bicalutamide and enzalutamide, which are androgen receptor blockers, can reduce the expression of TMPRSS2 or the entry of SARS-CoV-2 virus into host cells. This hypothesis has entered the clinical verification stage. In addition, CD147 expressed by host cells can bind to the spike protein of SARS-CoV-2 and participate in host cell invasion [71], indicating that the anti-CD147 antibody meplazumab may prevent SARS-CoV-2 infection [72].

Since the sixth edition of “Diagnosis and Treatment Protocol for COVID-19,” arbidol (200 mg tid) and chloroquine phosphate (500 mg bid) have been included in the plan. Arbidol has the characteristic core of indole, which can inhibit the fusion between the viral envelope and the host cell, thereby preventing the virus from entering the target cell [73]. A comparative analysis of protein sequences has revealed that the trimerization domain (S2) of the SARS-CoV-2 spike protein is similar to the hemagglutinin (HA) protein in influenza virus H3N2, which may be the binding site of arbidol [74]. Arbidol can also stimulate the humoral immune response and induce the production of interferon, thereby exhibiting a regulatory effect on the immune system [75]. However, the results of a retrospective analysis have shown that arbidol treatment cannot improve the symptoms of the disease or shorten the negative turning time of respiratory specimen virus nucleic acid [76]. However, chloroquine and hydroxychloroquine have been regarded as drug candidates with great therapeutic potential because they can block viral infection by increasing the endosomal pH value required for viral cell fusion [77] and inhibit viral replication through the suppression of p38 mitogen-activated protein kinase (MAPK) activation [78]. On March 28, 2020, the FDA issued an Emergency Use Authorization (EUA), allowing the distribution of hydroxychloroquine sulfate and chloroquine phosphate products donated to the Strategic National Stockpile to certain hospitalized patients with COVID-19 (www.fda.gov/media/138945/download). However, further clinical studies have revealed that chloroquine and hydroxychloroquine are not beneficial to hospitalized patients with COVID-19 and even have potential cardiac side effects. [79, 80] Therefore, on June 15, the FDA officially announced the withdrawal of the EUA of chloroquine and hydroxychloroquine for treating COVID-19 [81]. Recently, analogue mefloquine is under clinical trial.

#### 2.1.2 Blocking virus replication

*In vitro* studies have revealed that SARS-CoV-2 can infect human lung tissue more effectively and replicate more efficiently compared with SARS-CoV. The number of viral particles in lung tissues infected by SARS-CoV-2 is more than 3.2 times the number of SARS-CoV within 48 h. [82] Blocking viral replication is particularly important in anti-SARS-CoV-2 virus therapy. RNA-dependent RNA polymerase (RdRP) is a key enzyme in the life cycle of RNA viruses and one of the most promising drug targets in anti-coronavirus treatment. Compared with several other types of positive-sense RNA viruses (hepatitis C virus, Zika virus) [83], SARS-CoV-2 polymerase and several key amino acid residues conserved in the active site show the structural similarity with SARS-CoV (97%) [84, 85]. Therefore, the inhibition of RdRP for SARS-CoV drug development may have therapeutic potential for SARS-CoV-2. Chloroquine, hydroxychloroquine, and mefloquine can block membrane fusion, while also exhibiting an RdRP inhibitory effect [86].

Nucleoside analogue antiviral drugs, mainly including adenosine analogues, guanosine analogues, and pyrimidine analogues, also target RdRP. Among these, remdesivir is an adenosine analogue considered to be one of the most promising therapeutic drugs in the early stage of the virus outbreak. The first patient with COVID-19 in the United States [87] was given an intravenous infusion of remdesivir on the seventh day of admission, and the symptoms improved significantly ever since the next day. In the clinical trial of compassionate drug use [88], remdesivir has shown a certain clinical improvement effect, but the experiment lacks a control group and the median observation time is short, which needs further clinical verification. Currently, a Phase III clinical trial, including mild, moderate, and severe cases, of remdesivir is also underway. In the “Handbook of COVID-19 Prevention and Treatment” [89], darunavir/cobicistat is also recommended as an antiviral treatment. Taken together, it suggests that the drugs that can reduce viral replication may also be promising for treating COVID-19.

Ribavirin, also known as "tribavirin," is a nucleoside antiviral drug and a guanosine analogue, which has been approved by the FDA mainly for treating respiratory syncytial virus infections in adults and children (inhalation only). In Trial Version 8, it is recommended to use ribavirin in combination with α-interferon or lopinavir/ritonavir. α-Interferon is an important cytokine in innate immunity, which can enhance the killing activity of the immune system against virus-infected cells. In a clinical trial of α-interferon/ribavirin combined treatment of patients with severe MERS, the 28-day mortality rate was not improved, although the 14-day mortality rate was significantly reduced [90]. α-Interferon/Ribavirin combined treatment was also applied in SARS, but no significant improvement was observed in prognosis. [91] Other adenosine analogue investigational drugs used in treating SARS-CoV [85], such as galidesivi, favipiravir, atazanavir, daclatasvir, and oseltamivir, are currently in clinical trials. Similarly, nucleoside analogues, such as triazavirin, clevudine, and clofazimine, have also been included in clinical trials. In addition, computer matching analysis has found that the inhibitors of nucleoside transport, reverse transcriptase, and nucleic acid synthesis may have therapeutic potential, which have entered the clinical validation stage.

The coronavirus genome contains two open reading frames (ORF1a and ORF1b), which encode polyproteins. These polyproteins are processed by 3C-like protease (3CL) and a papain-like protease (PLpro), which can produce 16 kinds of nonstructural proteins, including RdRP [92]. Therefore, the addition of 3CL inhibitors can affect the production of RdRP, thereby reducing viral replication. In addition, the level of viral replication is regulated by the cellular *de novo* pyrimidine nucleotide biosynthesis pathway. Dihydroorotate dehydrogenase (DHODH) is a restriction enzyme on this pathway and can be a therapeutic target for antiviral drugs. By inhibiting sphingosine kinase 2, lipid metabolism can be changed, while phospholipid aggregation can be reduced, thereby limiting virus assembly. Even by inhibiting heat shock protein 90 (Hsp90), the virus hijacks infected cells through the process of autophagy, thus suppressing virus replication. These therapeutic regimens are currently in clinical trials. Other promising drugs such as azithromycin, carrimycin, and DAS181 are also in clinical trials. For example, in combination with antibiotics and proton pump inhibitors, the bismuth salt for treating *Helicobacter pylori* was found to inhibit both NTPase and SARS-CoV-2 nsp13 helicase, thereby reducing viral replication [93]

**Table 2:** Combined use of different treatments in COVID19.

Treatment	Drug	Drug target	Dosage	Source
Arbidol + Bromhexine	Arbidol	Membrane fusion inhibitor	PO	NCT04273763 (CN)
	Bromhexine	Expectorant	PO	
Arbidol + IFN-β1α	Arbidol	Membrane fusion inhibitor	PO, 200 mg, tid, 14 d	NCT04254874 (CN)
	IFN-β1α	Immunomodulator	INH, 14 d	
Chloroquine + Ivermectin	Chloroquine	Membrane fusion inhibitor and immunomodulator	PO	NCT04382846 (EG)
	Ivermectin	Importin (IMP) α/β receptor	PO	
Chloroquine + Ivermectin + Vitamin D	Chloroquine	Membrane fusion inhibitor and immunomodulator	PO, 500 mg, 4 d, 30 d	NCT04399746 (MX)
	Ivermectin	IMP α/β receptor	PO, 6 mg, qd, Day 1, 7 and 8	
	Vitamin D	Vitamins	PO, 400 UI, bid	
Chloroquine + Losartan	Chloroquine	Membrane fusion inhibitor and immunomodulator	PO, 450 mg, bid	NCT04428268 (MX)
	Losartan	Angiotensin II receptor (type AT1) antagonis	PO, 25 mg, bid	
Chloroquine + Zinc	Chloroquine	Membrane fusion inhibitor and immunomodulator	PO	NCT04447534 (EG)
	Zinc		PO	
Hydroxychloroquine + Azithromycin	Hydroxychloroquine	--/ Receptor binding and membrane fusion inhibitor	PO, 400 mg, bid, 7 d	NCT04321278 (IL) NCT04322123 (BR) NCT04329832 (US)
	Azithromycin	Tetrcycline	PO, 500 mg, qd	
Hydroxychloroquine + Azithromycin + / - tocilizumab	Hydroxychloroquine	Membrane fusion inhibitor and immunomodulator	PO, 800 mg, qd	NCT04347031 (RU)
	Azithromycin	Tetrcycline	PO, 250 mg, bid	
	Tocilizumab	Anti-IL-6R antibody	IV	
Hydroxychloroquine + Azithromycin + Convalescent plasma	Hydroxychloroquine	Membrane fusion inhibitor and immunomodulator	PO, 400 mg, bid, 5 d	NCT04441424 (IQ)
	Azithromycin	Tetrcycline	PO, 500 mg, qd, 5 d	
	Convalescent plasma	Immunomodulator	IV, 400 mL, 5 d	
Hydroxychloroquine + Azithromycin + Oseltamivir	Hydroxychloroquine	Membrane fusion inhibitor and immunomodulator	PO, 200 mg, tid, 5 d	NCT04338698 (BR) NCT04338698 (PK)
	Azithromycin	Tetrcycline	PO, 500 mg, Day 1; 250 mg, Day 2-5	
	Oseltamivir	Nucleoside analog	PO, 75 mg, bid, 5 d	
Hydroxychloroquine + Azithromycin + Sarilumab	Hydroxychloroquine	Membrane fusion inhibitor and immunomodulator	PO, 200 mg, tid	NCT04341870 (FR)
	Azithromycin	Tetrcycline	PO, 500 mg, Day 1; 250 mg, Day 2-5	
	Sarilumab	Anti-IL-6R antibody	IV, 400 mg, Day 1	
Hydroxychloroquine + Azithromycin + zinc	Hydroxychloroquine	Membrane fusion inhibitor and immunomodulator	PO, 600 mg, Day 1, 200 mg, Day 2-9	NCT04528927 (TN)
	Azithromycin	Tetrcycline	PO, 500 mg, Day 1; 250 mg, Day 2-5	
	Zinc		PO, 220 mg, 10 d	
Hydroxychloroquine + Baricitinib	Hydroxychloroquine	Membrane fusion inhibitor and immunomodulator	PO, 200 mg, tid, 14 d	NCT04373044 (US)
	Baricitinib	JAK inhibitor	PO, 2 mg, qd, 14 d	
Hydroxychloroquine + Bromhexine	Hydroxychloroquine	Membrane fusion inhibitor and immunomodulator	PO, 200 mg, tid	NCT04273763 (CN) NCT04340349 (MX)
	Bromhexine	Expectorant	PO, 8 mg, tid	
Hydroxychloroquine + Camostat mesylate	Hydroxychloroquine	Membrane fusion inhibitor and immunomodulator	PO, 400 mg, bid, 10 d	NCT04355052 (IL)
	Camostat mesylate	TMPRSS2 inhibitor	PO, 200 mg, qd, 10 d	
Hydroxychloroquine + Clindamycin	Hydroxychloroquine	Membrane fusion inhibitor and immunomodulator	PO, 200 mg, tid, 7 d	NCT04349410 (US)
	Clindamycin	Lincomycin antibiotics	IV, 4800 mg	
Hydroxychloroquine + Clindamycin + Primaquine	Hydroxychloroquine	Membrane fusion inhibitor and immunomodulator	PO, 200 mg, tid, 7 d	NCT04349410 (US)
	Clindamycin	Lincomycin antibiotics	IV, 4800 mg, 7 d	
	Primaquine	Membrane fusion inhibitor and immunomodulator	PO, 200 mg, qd, 7 d	
Hydroxychloroquine + Daclatasvir + Sofosbuvir	Hydroxychloroquine	Membrane fusion inhibitor and immunomodulator	PO, 400 mg, bid, 14 d	NCT04443725 (EG)
	Daclatasvir	Nucleoside analog	PO, 90 mg, qd, 14 d	
	Sofosbuvir	Nucleoside analog	PO, 400 mg, qd, 14 d	
Hydroxychloroquine + Doxycycline	Hydroxychloroquine	Membrane fusion inhibitor and immunomodulator	PO, 200 mg, tid, 10 d	NCT04349410 (US)
	Doxycycline	Tetrcycline	IV, 100 mg, bid, 10 d	
Hydroxychloroquine + Favipiravir	Hydroxychloroquine	Membrane fusion inhibitor and immunomodulator	PO, 200 mg, bid, 7 d	NCT04359615 (IR) NCT04376814 (IR)
	Favipiravir	Nucleoside analog	PO, 1600 mg, Day 1, 600 mg, tid, 7 d	
Hydroxychloroquine + IFN-α2β	Hydroxychloroquine	Membrane fusion inhibitor and immunomodulator	PO	NCT04273763 (CN)
	IFN-α2β	Immunomodulator	INH	
Hydroxychloroquine + Imatinib	Hydroxychloroquine	Membrane fusion inhibitor and immunomodulator	PO, 200 mg, bid	NCT04346147 (ES)
	Imatinib	TMPRSS2 inhibitor	PO, 400 mg, qd	
Hydroxychloroquine + Indomethacin +Azithromycin	Hydroxychloroquine	Membrane fusion inhibitor and immunomodulator	PO, 200 mg, bid, 7d	NCT04344457 (US)
	Indomethacin	Nonsteroidal anti-inflammatory drug	PO, 50 mg, tid, 14 d	
	Zithromax	Tetrcycline	PO, 500 mg, qd, 3d	
Hydroxychloroquine + Lopinavir + Ritonavir + IFN-β1α	Hydroxychloroquine	Membrane fusion inhibitor and immunomodulator	PO	NCT04350684 (IR) NCT04343768 (IR) NCT04350671 (IR)
	Lopinavir	Nucleoside analog	PO	
	Ritonavir	Nucleoside analog	PO	
	IFN-β 1a	Immunomodulator	IV	
Hydroxychloroquine + Lopinavir + Ritonavir	Hydroxychloroquine	Membrane fusion inhibitor and immunomodulator	PO, 200 mg, bid	NCT04390152 (CO) NCT04346147 (ES)
	Lopinavir	Nucleoside analog	PO, 200 mg, qd	
	Ritonavir	Nucleoside analog	PO, 50 mg, qd	
Hydroxychloroquine + Lopinavir + Ritonavir + IFN-β1β	Hydroxychloroquine	Membrane fusion inhibitor and immunomodulator	PO	NCT04343768 (IR)
	Lopinavir	Nucleoside analog	PO	
	Ritonavir	Nucleoside analog	PO	
	IFN-β1β	Immunomodulator	PO	
Hydroxychloroquine + Oseltamivir	Hydroxychloroquine	Membrane fusion inhibitor and immunomodulator	PO, 200 mg, bid, 5 d	NCT04338698 (PK) NCT04303299 (TH)
	Oseltamivir	Nucleoside analog	PO, 75 mg, bid, 5 d	
Hydroxychloroquine + Sirolimus f	Hydroxychloroquine	Membrane fusion inhibitor and immunomodulator	PO, 600 mg, 10 d	NCT04374903 (JO)
	Sirolimus	Immunosuppressant	PO, 250 mg, 10 d	
Hydroxychloroquine + Tofacitinib	Hydroxychloroquine	Membrane fusion inhibitor and immunomodulator	PO, 200 mg, tid, 14 d	NCT04390061(US)
	Tofacitinib	JAK inhibitor	PO, 10 mg, bid, 14 d	
Azithromycin + Amoxicillin	Azithromycin	Tetrcycline	PO, 500 mg, Day 1; 250 mg, Day 2-5, 2 d	NCT04363060 (FR)
	Amoxicillin/Clavulanate	Antibacterial drugs	PO, 250 mg, tid, 2 d	
Azithromycin + Atovaquone	Azithromycin	Tetrcycline	PO, 500 mg, Day 1; 250 mg, Day 2-5	NCT04339426 (US)
	Atovaquone		PO, 750 mg, bid, 10 d	
Azithromycin + Clavulanate	Azithromycin	Tetrcycline	PO, 250 mg, tid, 7 d	NCT04363060 (FR)
	Clavulanate			
Azithromycin + Doxycycline	Azithromycin	Tetrcycline	PO, 500 mg, Day 1; 250 mg, Day 2-5	NCT04528927 (TN)
	Doxycycline	Tetrcycline	PO, 200 mg, qd, 10 d	
Azithromycin + Ivermectin + Nitazoxanide	Azithromycin	Tetrcycline	PO	NCT04382846 (EG)
	Ivermectin	IMP α/β receptor	PO	
	Nitazoxanide	Immunomodulator	PO	
Azithromycin + Mefloquine + / - Tocilizumab	Azithromycin	Tetrcycline	PO, 250 mg, tid, 7 d	NCT04347031 (RU)
	Mefloquine	Membrane fusion inhibitor and immunomodulator	PO, 500 mg, bid, 7 d	
	Tocilizumab	Anti-IL-6R antibody	IV	
Azithromycin + Nitazoxanide	Azithromycin	Tetrcycline	PO	NCT04382846 (EG)
	Nitazoxanide	Immunomodulator	PO	
Azithromycin + Oseltamivir	Azithromycin	Tetrcycline	PO, 500 mg, Day 1; 250 mg, Day 2-5	NCT04338698 (PK)
	Oseltamivir	Nucleoside analog	PO, 75 mg, bid	
Azythromycin + Ivermectin + Dutasteride	Azythromycin	Tetrcycline	PO, 500 mg, qd	NCT04446429 (BR)
	Ivermectin	IMP α/β receptor	PO, 200 mcg/kg, qd	
	Dutasteride	TMPRSS2 inhibitor	PO, 0.5 mg	
Azythromycin + Ivermectin+ Proxalutamide	Azythromycin	Tetrcycline	PO, 500 mg, qd	NCT04446429 (BR)
	Ivermectin	IMP α/β receptor	PO, 200 mcg/kg, qd	
	Proxalutamide	TMPRSS2 inhibitor	PO, 200 mg	
Ivermectin + Doxycycline	Ivermectin	IMP α/β receptor	PO, 200 mcg/kg, qd, 5 d	NCT04407130 (BD) NCT04523831 (BD) NCT04403555 (EG)
	Doxycycline	Tetrcycline	PO, 200 mg, 5 d	
Ivermectin + Dutasteride +	Ivermectin	IMP α/β receptor	PO, 200 mcg/kg, qd	NCT04446429 (BR)
	Dutasteride	TMPRSS2 inhibitor	PO, 0.5 mg	
Ivermectin + Losartan	Ivermectin	IMP α/β receptor	PO, 12 mg, qd, 15 d	NCT04447235 (BR)
	Losartan	Angiotensin II receptor (type AT1) antagonis	PO, 50 mg, qd, 15 d	
Ivermectin + Nitazoxanide	Ivermectin	IMP α/β receptor	PO	NCT04382846 (EG)
	Nitazoxanide	Immunomodulator	PO	
Ivermectin + Nitazoxanide + Ribavirin	Ivermectin	IMP α/β receptor	PO, 7 d	NCT04392427 (EG)
	Nitazoxanide	Immunomodulator	PO, 7 d	
	Ribavirin	Nucleoside analog	PO, 7 d	
Ledipasvir + Sofosbuvir	Ledipasvir	Nucleoside analog	PO, 90 mg, qd, 14 d	NCT04498936 (EG)
	Sofosbuvir	Nucleoside analog	PO, 400 mg, qd, 14 d	
Daclatasvir + Sofusbuvir	Daclatasvir	Nucleoside analog	PO, 120 mg, Day 1; 60 mg, Day 2-9	NCT04468087 (BR) NCT04460443 (EG)
	Sofusbuvir	Nucleoside analog	PO, 800 mg, Day 1; 400 mg, Day 2-9	
Danoprevir + Ritonavir	Danoprevir	Nucleoside analog	PO, 100 mg, bid, 10 d	NCT04345276 (CN) NCT04291729 (CN)
	Ritonavir	Nucleoside analog	PO, 100 mg, bid	
Darunavir + Cobicistat	Darunavir	Viral RNA-dependent RNA polymerase inhibitor/ CYP3A protein inhitor	PO, 800 mg, qd, 5 d	NCT04252274 (CN) NCT04425382 (QA)
	Cobicistat	Protease inhibitor	PO, 150 mg, qd, 5 d	
Darunavir + Ritonavir + Favipiravir+ Hydroxychloroquine	Darunavir	Nucleoside analog	PO, 400 mg, tid	NCT04303299 (TH)
	Ritonavir	Nucleoside analog	PO, 200 mg, qd	
	Favipiravir	Nucleoside analog	PO, 2400 mg, qd	
	Hydroxychloroquine	Membrane fusion inhibitor and immunomodulator	PO, 800 mg, qd	
Darunavir + Ritonavir + Oseltamivir + Hydroxychloroquine	Darunavir	Nucleoside analog	PO, 400 mg, tid	NCT04303299 (TH)
	Ritonavir	Nucleoside analog	PO, 200 mg, qd	
	Oseltamivir	Nucleoside analog	PO, 300 mg, qd	
	Hydroxychloroquine	Membrane fusion inhibitor and immunomodulator	8 PO, 00 mg, qd	
Darunavir + Ritonavir + Oseltamivir	Darunavir	Nucleoside analog	PO, 400 mg, tid	NCT04303299 (TH)
	Ritonavir	Nucleoside analog	PO, 200 mg, qd	
	Oseltamivir	Nucleoside analog	PO, 300 mg, qd	
Emtricitabine + Tenofovir	Emtricitabine	Protease inhibitor	PO, 300 mg, qd, 8 d	NCT04519125 (CO)
	Tenofovir	Nucleoside analog	PO, 200 mg, qd	
Emtricitabine + Tenofovir Alafenamide	Emtricitabine	Protease inhibitor	PO, 200 mg, qd	NCT04405271 (AR)
	Tenofovir alafenamide	Nucleoside analog	PO, 25 mg, qd	
Favipiravir + Maraviroc	Favipiravir	Nucleoside analog	PO, 200 mg, qd, 10 d	NCT04475991 (MX)
	Maraviroc	Chemokine receptor antagonist	PO, 300 mg, qd, 10 d	
Favipiravir + Tocilizumab	Favipiravir	Nucleoside analog	PO, 1600 mg, Day 1, 600 mg, tid, 6 d	NCT04310228 (CN)
	Tocilizumab	Anti-IL-6R antibody	IV, 4-8 mg/kg, 7 d	
Oseltamivir + ASC09F	Oseltamivir	RdRP inhibitor	PO, 75 mg, qd, 14 d	NCT04261270 (CN)
	ASC09F	CYP3A4 inhibitor	PO, 400 mg, bid, 14 d	
Oseltamivir + Mesenchymal stem cells	Oseltamivir	Nucleoside analog	PO, 4 w	NCT04371601 (CN)
	Mesenchymal stem cells	MSC therapy	IV, 1 ×10^6 cell/kg/w, 4 w	
Oseltamivir + Ritonavir	Oseltamivir	Viral RNA-dependent RNA/ Booster of other protease polymerase inhibitor	PO, 75 mg, qd, 7 d	NCT04315896 (MX) NCT04318444 (US) NCT04328285 (FR)
	Ritonavir	Nucleoside analog	PO, 300 mg, bid, 7 d	
Lopinavir + Ritonavir	Lopinavir	Anti-retroviral of the protease inhibitor/booster of other protease inhibitors	PO, 400 mg, bid, 5 d	Guidelines (version 7) for treatment of COVID-19 NCT04328285 (FR) NCT04328012 (US)
	Ritonavir	Nucleoside analog	PO, 100 mg, bid, 5 d	
Lopinavir + Ritonavir + Arbidol	Lopinavir	Anti-retroviral of the protease inhibitor/booster of other protease inhibitors	PO, 400 mg, bid, 5-21 d	NCT04350671 (IR) NCT04403100 (BR) NCT04376814 (IR)
	Ritonavir	Nucleoside analog	PO, 100 mg, bid, 5-21 d	
	Arbidol	Membrane fusion inhibitor and immunomodulator/Anti-retroviral of the protease inhibitor/booster of other protease	PO, 200 mg, tid, 5-21 d	
Lopinavir + Ritonavir + Atorvastatin	Lopinavir	Anti-retroviral of the protease inhibitor/booster of other protease inhibitors	PO, 200 mg, qd, 10 d	NCT04466241 (FR)
	Ritonavir	Nucleoside analog	PO, 50 mg, qd, 10 d	
	Atorvastatin	Statin medication	PO, 20 mg, qd, 10 d	
Lopinavir + Ritonavir + Favipiravir	Lopinavir	Anti-retroviral of the protease inhibitor/booster of other protease inhibitors	PO, 400 mg, bid, 7 d	NCT04499677 (GB) NCT04303299 (TH)
	Ritonavir	Nucleoside analog	PO, 100 mg, bid, 7 d	
	Favipiravir	Nucleoside analog	PO, 1800 mg, bid, Day 1; 400 mg, 4 times, 7 d	
Lopinavir + Ritonavir + IFN-β1α	Lopinavir	Anti-retroviral of the protease inhibitor/booster of other protease inhibitors	PO, 200 mg, qd	NCT04315948 (FR) NCT04276688 (CN)
	Ritonavir	Nucleoside analog	PO, 50 mg, qd	
	IFN-β1α	Immunomodulator	INH, 44 μg/ 0.5 mL	
Lopinavir + Ritonavir + Oseltamivir	Lopinavir	Anti-retroviral of the protease inhibitor/booster of other protease inhibitors	PO, 800 mg, qd	NCT04303299 (TH)
	Ritonavir	Nucleoside analog	PO, 200 mg, qd	
	Oseltamivir	Nucleoside analog	PO, 300 mg, qd	
Lopinavir + Ritonavir + Telmisartan	Lopinavir	Anti-retroviral of the protease inhibitor/booster of other protease inhibitors	PO, 200 mg, qd, 10 d	NCT04466241 (FR)
	Ritonavir	Nucleoside analog	PO, 50 mg, qd, 10 d	
	Telmisartan	Angiotensin II receptor (type AT1) antagonis	PO, 40 mg, qd, 10 d	
Lopinavir + Ritonavir +Ribavirin + IFN-β1α	Lopinavir	Anti-retroviral of the protease inhibitor/booster of other protease inhibitors	PO, 400 mg, bid, 14 d	NCT04276688 (CN) NCT04343768 (IR)
	Ritonavir	Nucleoside analog	PO, 100 mg, bid, 14 d	
	Ribavirin	Nucleoside analog	PO, 400 mg, bid, 14 d	
	IFN-β1α	Immunomodulator	SC, 0.25 mg	
Remdesivir + Apremilast	Remdesivir	Nucleoside analog	PO, 200 mg, qd, Day 1; 100 mg, qd, Day 2-9	NCT04488081 (US)
	Apremilast	Antiemetic	PO, 30 mg, bid, 9 d	
Remdesivir + Cenicriviroc	Remdesivir	Nucleoside analog	PO, 200 mg, qd, Day 1; 100 mg, qd, Day 2-9	NCT04488081 (US)
	Cenicriviroc	CCR5 inhibitor	PO, 150 mg, bid, 28 d	
Remdesivir + Baricitinib	Remdesivir	Nucleoside analog	PO, 200 mg, qd, Day 1; 100 mg, qd, Day 2-9	NCT04401579 (US)
	Baricitinib	JAK inhibitor	PO, 4 mg, qd, 14 d	
Remdesivir + Icatibant	Remdesivir	Nucleoside analog	PO, 200 mg, qd, Day 1; 100 mg, qd, Day 2-9	NCT04488081 (US)
	Icatibant	Peptide-based hormone	SC, 30 mg, 9 d	
Remdesivir + IFN-β1α	Remdesivir	Nucleoside analog	PO, 200 mg, qd, Day 1; 100 mg, qd, Day 2-9	NCT04492475 (US)
	IFN-β1α	Immunomodulator	SC, 44 μg/ 0.5 mL	
Remdesivir + Merimepodib	Remdesivir	Nucleoside analog	PO, 200 mg, qd, Day 1; 100 mg, qd, Day 2-9	NCT04410354 (US)
	Merimepodib	Inosine monophosphate dehydrogenase (IMPDH) inhibitor	IV, 400 mg, tid, 10 d	
Remdesivir + NA-831	Remdesivir	Nucleoside analog	PO, 1 mg/kg	NCT04480333 (US)
	NA-831	Endogenous small molecule	INH, 0.2 mg/kg	
Remdesivir + Razuprotafib	Remdesivir	Nucleoside analog	PO, 200 mg, qd, Day 1; 100 mg, qd, Day 2-9	NCT04488081 (US)
	Razuprotafib	VE-PTP inhibitor	SC, 10 mg, tid, 7d	
Remdesivir + Tocilizumab	Remdesivir	Nucleoside analog	PO, 10 d	NCT04409262 (US)
	Tocilizumab	Anti-IL-6R antibody	IV, 10 d	
Ribavirin + Sofosbuvir	Ribavirin	Nucleoside analog	PO	NCT04460443 (EG)
	Sofosbuvir	Nucleoside analog	PO	
Ritonavir + ASC09	Ritonavir	Nucleoside analog	PO, 100 mg, bid, 14 d	NCT04261907 (CN)
	ASC09	Protease inhibitors	PO, 300 mg, bid, 14 d	
Ritonavir + Ganovo + IFN-Nebulization	Ritonavir	Nucleoside analog	PO, 100 mg, bid, 14 d	NCT04291729 (CN)
	Danoprevir	Nucleoside analog	PO, 100 mg, bid, 14 d	
	IFN-Nebulization	Immunomodulator	INH, 50 μg, bid, 14 d	
IFN-α1β + Thymosin α1	IFN-α1β	Immunomodulator	ISIN, 2-3 drops, 4 times	NCT04320238 (CN)
	Thymosin α1	Immunomodulator	SC, 1 time per week	
IFN-β1β + clofazimine	IFN-β1β	Immunomodulator	SC or IV, 16 million UI, 3 d	NCT04465695 (CN)
	Clofazimine	Nucleoside analog	PO, 100 mg, bid	
Adalimumab + Tocilizumab	Adalimumab	Humanized monoclonal antibody against the TNF-alpha	Adalimumab: SC, 40 mg, every 2 wekks; Tocilizumab: IV, 8 mg/kg, 6 times in 4 weeks	ChiCTR2000030580
	Tocilizumab	Anti-IL-6R antibody	IV	
	C486	-	SC or IV	
REGN10933 + REGN10987	REGN10933	Anti-Spike (S) SARS-CoV-2 antibody	SC or IV	NCT04426695 (US) NCT04519437 (US) NCT04452318 (US)
	REGN10987			
Tocilizumab + Dexamethasone	Tocilizumab	Anti-IL-6R antibody	IV, 8 mg/kg, Day 1 and 3	NCT04476979 (GF)
	Dexamethasone	Corticosteroids	IV, 10 mg for 5 d, 2.5 mg for 4 d	
Tocilizumab + Methylprednisolone	Tocilizumab	Anti-IL-6R antibody	IV, 8 mg/kg, Day 1 and 3	NCT04377503 (ES)
	Methylprednisolone	Corticosteroids	IV, 1.5 mg/kg/d, 21 d	
Tocilizumab + Pembrolizumab	Tocilizumab	Anti-IL-6R antibody	IV, 8 mg/kg	NCT04335305 (ES)
	Pembrolizumab	PD-1 antibody	IV, 200 mg	
Toremifene + Melatonin	Toremifene	Hormone	PO, 60 mg, qd	NCT04531748 (US)
	Melatonin	Hormone	PO, 40 mg, morning; 60 mg, evening	
Anakinra + Ruxolitinib	Anakinra	IL antagonists	IV, 300 mg/d, 5 d	NCT04366232 (FR)
	Ruxolitinib	JAK inhibitor	PO, 5 mg, bid, 14-28 d	
Anakinra + Siltuximab	Anakinra	IL antagonists	SC, 100 mg, qd	NCT04330638 (BE)
	Siltuximab	Anti-IL-6R antibody	IV, 11 mg/kg	
Anakinra + Tocilizumab	Anakinra	IL antagonists	SC, 100 mg, qd	NCT04330638 (BE)
	Tocilizumab	Anti-IL-6R antibody	IV, 8 mg/kg	
Colchicine + Edoxaban	Colchicine	NLRP Inflammasome inhibitor	PO, 0.5 mg, qd	NCT04516941 (CH)
	Edoxaban	Thrombolytic medication	PO, 60 mg, qd	
Colchicine + Methylprednisolone	Colchicine	NLRP Inflammasome inhibitor	PO, 0.5 mg, qd, 14 d	NCT04492358 (ES)
	Methylprednisolone	Corticosteroids	PO, 60 mg, qd, 3d	
Colchicine + Rosuvastatin	Colchicine	NLRP Inflammasome inhibitor	PO, 0.6 mg, qd, 3 d	NCT04472611 (US)
	Rosuvastatin	HMG-CoA reductase inhibitors	PO, 40 mg, qd, 3 d	
Dexamethasone + Placenta-Derived MMSCs	Dexamethasone	Corticosteroids	IV	NCT04461925 (UA)
	Placenta-Derived MMSCs	MSC therapy	IV, 1 ×10^6 cell/kg, Day 1, 4 and 7	
Diltiazem + Niclosamide	Diltiazem	Calcium-channel blocker	PO, 500 mg × 4 times, 10 d	NCT04372082 (FR)
	Niclosamide	-	PO, 60 mg, tid, 10 d	
Dipyridamole + Aspirin	Dipyridamole	Thrombolytic medication	PO, 200 mg, qd, 14 d	NCT04410328 (US)
	Aspirin	Thrombolytic medication	PO, 25 mg, qd, 14 d	
Enoxaparin + Methylprednisolone	Enoxaparin	Thrombolytic medication	SC, 4000-6000 UI	NCT04528888 (IT)
	Methylprednisolone	Corticosteroids	IV, 0.5 mg/kg	
Heparin + Methylprednisolone	Heparin	Thrombolytic medication	IV, 18 UI/kg/h	NCT04485429 (BR) NCT04528888 (IT)
	Methylprednisolone	Corticosteroids	IV, 0.5 mg/kg	
Heparin + Umbilical Cord Mesenchymal Stem Cells	Heparin	Thrombolytic medication	IV	NCT04355728 (US)
	Umbilical Cord Mesenchymal Stem Cells	MSC therapy	IV, 100 ×10^6 cell	
Levamisole + Isoprinosine	Levamisole	-	PO, 50 mg, tid, 14 d	NCT04383717 (EG) NCT04360122 (EG)
	Isoprinosine	-	PO, 1 g, 4 times	
Metenkefalin + Tridecactide	Metenkefalin	Opioid delta receptor agonists	IV, 5 mg	NCT04374032 (YU)
	Tridecactide	Th1 cell modulators	IV, 1 mg	
Nitazoxanide + atazanavir+ ritonavir	Nitazoxanide	Immunomodulator	PO, 1000 mg, bid	NCT04459286 (NG)
	Atazanavir	Nucleoside analog	PO, 300 mg, qd	
	Ritonavir	Nucleoside analog	PO, 100 mg, qd	
Paracetamol + ChAdOx1 nCoV-19	Paracetamol	Non-steroidal anti-inflammatory drugs	PO,	NCT04324606 (GB)
	ChAdOx1 nCoV-19	Vaccine	IV, 5 ×10^10 vp	
Paracetamol + MenACWY	Paracetamol	Non-steroidal anti-inflammatory drugs	IV	NCT04324606 (GB)
	MenACWY	Vaccine	IV	
Ruxolitinib + Simvastatin	Ruxolitinib	JAK inhibitor	PO, 5 mg, bid, 14 d	NCT04348695 (ES)
	Simvastatin	Statin medication	PO, 40 mg, qd, 14 d	
SnPP Protoporphyrin + Sunlight exposure	SnPP Protoporphyrin	Photodynamic therapy	IV, 5, 7 and 9 mg, 14 d	NCT04371822 (EG)
	Sunlight exposure		1 h, 14 d	
Sulfonatoporphyrin(TPPS) + Sunlight exposure	Sulfonatoporphyrin(TPPS)	Photodynamic therapy	IV, 5 mg, 14 d	NCT04371822 (EG)
	Sunlight exposure		1 h, 14 d	
Tacrolimus + Methylprednisolone	Tacrolimus	Immunosuppressant	PO, 8-10 ng/mL blood level	NCT04341038 (ES)
	Methylprednisolone	Corticosteroids	PO, 120 mg, 3 d	
Tirofiban + Clopidogrel + Acetylsalicylic acid + Fondaparinux	Tirofiban	Thrombolytic medication	IV, 0.15 μg/kg/min	NCT04368377
	Clopidogrel	Thrombolytic medication	PO, initial dose of 300 mg, then 75 mg/d	
	Acetylsalicylic acid	Thrombolytic medication	IV, initial dose of 75 mg, then 30 mg/d	
	Fondaparinux	Thrombolytic medication	IV, 2.5 mg	

PO: Oral administration; IV: Intravenous administration; INH: Inhalation; IM: intramuscular administration; SC: Subcutaneous administration; ISIN: Intrasinal administration; ID: intradermal injection; SL: Sublingual administration; TOPp: patch applied on the skin; ET: Endotracheal instillation

#### 2.1.3 Virus removal

Clinically, the immune response induced by SARS-CoV-2 infection is divided into two stages [94]. In the early and nonsevere stage, a specific adaptive immune response is required to eliminate the virus and prevent the disease from progressing to the severe stage. In the severe stage, a large number of damaged cells induce an excessive immune response, mainly an innate immune response. Therefore, in the first stage, strategies to enhance the immune response (antiserum, hyperreactive immunoglobulin, or interferon (IFN)) are essential. In this process, IFN, interleukin 7 (IL-7), and thymosin are used to stimulate the production of T cells and activate T-cell functions to clear the virus. The Trial Version 8 has mentioned α-interferon (5 million U, bid, nebulized inhalation) or its combination with ribavirin (500 mg, bid or tid, intravenous infusion) for antiviral treatment. Other drugs such as Toll-like receptor agonist (PUL-042 and polyinosinic:polycytidylic acid), Bacillus Calmette-Guérin, and bacterial lysate, which can enhance lung epithelial mucosa or innate immunity of the body, are also in clinical trials [95-97].

Members of the coronavirus family, including SARS-CoV-2, are associated with strong interferon suppression, including blood lymphopenia-related abnormality of NK cells [98]. Natural killer (NK) cells are important lymphocytes in the innate immune system to fight infection. The differentiation of NK cells from stem cells and cord blood cells is currently under clinical investigation (Clinical trial identifier: NCT04299152, NCT04324996). NK cells expressing ACE2 and natural killer group 2D (NKG2D) constructed by chimeric antigen receptor T cell (CART) technology can enhance the antiviral effect by directly targeting the S proteins of SARS-CoV-2 and NKG2DL on the surface of infected cells (Clinical trial identifier: NCT04324996). The S protein-binding antibody or virus-neutralizing antibody developed for SARS-CoV-2 is also a research hotspot of antiviral drugs.

#### 2.1.4 Combined use of different antiviral treatments

New drug development and research are time-consuming and cannot cope with the current virus outbreak. Drug repurposing is a popular exploratory method, including exploring new indications from old drugs [99]. Currently, most of the drugs clinically used to fight the virus are for influenza, viral hepatitis, and HIV, as well as drugs developed for MERS and SARS [100-170] (Supplementary Table 1). However, since the viral outbreak, many exploratory clinical trials of antiviral treatments have been published. Unfortunately, no specific drug has been developed to fight the virus. Another method of rapid development strategy is based on the mechanism for the combination therapy of different antiviral drugs.

Trial Version 8 also mentions the antiviral drug lopinavir/ritonavir (lopinavir: 400 mg, bid; ritonavir: 100 mg, bid, oral), with the commercial name of Aluvia. Lopinavir is an anti-retroviral of the protease inhibitor, while ritonavir is often used as a booster of other protease inhibitors. It has been considered as a promising therapeutic drug in the early stage of the epidemic, recommended from the first version of the Diagnosis and Treatment Protocol for Novel Coronavirus Pneumonia to the seventh version. However, in a clinical study including 199 patients with severe COVID-19, lopinavir/ritonavir did not significantly improve the 28-day mortality rate compared with standard treatment [171]. The updated Trial Version 8 recommended a combination with ribavirin. An open-label, randomized, Phase II clinical trial showed that the early use of INF-β1, lopinavir/ritonavir, and ribavirin triple therapy could significantly shorten the time for nasopharyngeal swab virus to turn negative, reduce the hospital stay, and relieve symptoms for patients with mild-to-moderate COVID-19, compared with the application of lopinavir/ritonavir. A large number of clinical trials of hydroxychloroquine, azithromycin, chloroquine, lopinavir/ritonavir, or nucleoside analogue antiviral drugs in combination with drugs of different antiviral mechanisms (Table 2) have been performed to observe the effectiveness of the combined treatments for antiviral therapy. This drug development strategy saves a lot of preclinical research and can also avoid the embarrassing situation of poor efficacy of a single drug.

Although antiviral drugs seem to be promising for treating COVID-19, their potential toxicities and side effects still need attention. In addition, the dosage and starting time point of antiviral drugs require clinical investigation. The large-scale administration can make the virus mutate under selection pressure and produce drug resistance because the new coronavirus has different variants [172] or different mutations [173, 174].

### 2.2 Immunomodulatory therapy

In the severe stage of COVID-19, when the immune system is overactivated to combat the virus, multiple inflammatory factors are produced. It results in a severe cytokine storm through cascade amplification, continuous activation, and expansion of immune cells (even some T cells and B cells) (Fig. 1), leading to organ damage, edema, subsequent air exchange dysfunction, acute respiratory distress syndrome (ARDS), acute heart injury, multiple-organ failure, and death eventually [175]. These injuries persist if only antiviral therapy is given, and the excessive immune response is ignored. From this point, immune regulation may be the key to the treatment of patients with severe COVID-19.

Corticosteroids can antagonize pathophysiological processes of ARDS, mainly to inhibit excessive inflammation, excessive cell proliferation, and abnormal collagen deposition. At present, the application of corticosteroids in SARS-CoV-2 infection is inconclusive [176]; however, they are widely used for treating SARS-CoV [177] and MERS-CoV infections [178]. A retrospective study of 401 patients with SARS found that the administration of corticosteroids at lower doses reduced the mortality of patients with severe SARS and shortened their hospital stay, which was not observed in patients with severe secondary lower respiratory infections or other complications [177]. Corticosteroids and immunomodulators were usually prescribed empirically in the early stages of the COVID-19 outbreak. The application of corticosteroids in COVID-19 is still controversial due to the methodological limitations of the available evidence [176]. In the WHO-issued interim guidelines on “Clinical management of severe acute respiratory infection when COVID-19 is suspected” (published on March 13, 2020) [179], corticosteroids are not recommended unless otherwise indicated [180]. Nevertheless, some scholars think that the application of pulsed dose or long-term high dose, or early administration of corticosteroids to treat serious viral pneumonia can affect virus clearance rate [178, 181] or survival [182]. On the contrary, some researchers found that adjuvant therapy with low-dose corticosteroids could reduce mortality in critical cases [183]. A retrospective analysis showed that the rational use of low-dose corticosteroids could suppress the cytokine storm and prevent secondary multiple-organ damage in patients with severe COVID-19, thus gaining time for intensive treatment to bring survival advantages [184]. A systematic review and meta-analysis found that the application of corticosteroids in SARS-CoV-2, SARS-CoV, and MERS-CoV infections was associated with the delay in virus clearance, with a mean difference (MD) = 3.78 days (95% CI = 1.16), while the duration of hospital stay was prolonged and the use of mechanical ventilation increased. The Diagnosis and Treatment Protocol for COVID-19 (Trial Version 8) [49] has pointed out that a low dose of corticosteroids 3-5 days, not exceeding 1-2 mg/(kg.d) of methylprednisolone equivalent is appropriate for patients with a worsening oxygenation index, rapid imaging progression, and overactivation of an inflammatory response, whereas a high dose of corticosteroids can result in a strong immunosuppressive effect, thereby delaying the clearance of the virus. In short, corticosteroids should be used with caution in patients with COVID-19, and the indications and dosage should be strictly followed.

Intravenous immunoglobulin (IVIG) has been used to treat patients with autoimmunity and chronic inflammatory diseases; it targets viral, bacterial, and fungal infections [185]. IVIG can fight against infection by regulating a variety of mechanisms, such as downregulation of pro-inflammatory cytokines, upregulation of anti-inflammatory cytokines [186, 187], inhibition of Th1-involved delayed hypersensitivity inflammation, and inhibition of Th1-involved autoimmunity and chronic inflammation [187], as well as neutralizing pathogens. The guideline has pointed out that IVIG may be considered for critical and severe cases [102]. IVIG is derived from nonspecific gamma globulin (IgG type) extracted from the plasma of healthy people. The antibodies in the plasma of the recovered patients can inhibit viremia. According to recent clinical experiences and literature reports [188], the IgM type usually appears 3-4 days after the onset of COVID-19 symptoms, while the titer of IgG antibody can be higher than four times in the recovery stage compared with the acute stage. Therefore, convalescent plasma has been suggested as a therapeutic approach [189]. A clinical study revealed that four patients who received mechanical ventilation and ECMO no longer needed respiratory support, while the CT features recovered within 1-12 days with the negative viral load 9 days after receiving convalescence plasma from patients with severe COVID-19 [190]. The National Health Commission of China has also released the "Convalescent plasma for treating severe and critical COVID-19 (Trial Version 2)" [191], which incorporates the plasma of the rehabilitated patients into comprehensive treatment.

Pneumonia and lung injury associated with COVID-19 are accompanied by lymphocytic decline and interferon suppression, which is part of virus-induced immunosuppression. As mentioned earlier, the progressive decrease in the number of lymphocytes, the progressive increase in plasma IL-6 and CRP levels, the increase in the lactate level, as well as the rapid development of medical image processing are the predictors of severe cases. The levels of IL-1β, IL-6, IL-12, IL-8, tumor necrosis factor (TNF)-α, and CCL2 are usually elevated in infected people [192-194]. SARS-CoV can induce the production of more IL-6 in human epithelial cells, compared with influenza A virus and human parainfluenza virus Type II (HPIV II), which can be the basis for the expansion of IL-6 response [98, 195]. Trial Version 7 has recommended that severe cases with an elevated IL-6 level can be treated with tocilizumab [102]. A retrospective observational study found that the application of tocilizumab could reduce the risk of death by 24% [196]. Meanwhile, humanized monoclonal antibodies that antagonize IL-6R, such as clazakizumab, sarilumab, and siltuximab, are also in clinical trials. In addition, monoclonal antibodies infliximab, emapalumab, BMS-986253, canakinumab, and anakinra, which can antagonize TNF-α, IFN-γ, IL-8, IL-1, and IL-1R, are also in clinical exploration to prove their efficacy in treating COVID-19-related CS. Granulocyte-macrophage colony-stimulating factor (GM-CSF) is an important myelopoietic factor and pro-inflammatory cytokine. Myeloid cells activated by GM-CSF can secrete reactive oxygen species and express high levels of pro-inflammatory cytokines (IL-1, IL-6, and TNF) and various chemokines (CCL2, IL-8, and CCL17) [197]. GM-CSF can also enhance CD4+ T helper cells (T_H_) [198]. These T_H_ cells can further enhance the immune response generated by pro-inflammatory myeloid cells [199]. The iInhibition of GM-CSF has shown benefits in conditions of high inflammation [200]. Triggering receptor expressed on myeloid cells 1 (TREM1) can also promote inflammation during this process. [201] Pattern recognition receptors [Toll or Toll-like receptors (TLR)] can recognize pathogens (pathogen-related molecular patterns or pathogen-associated molecular pattern (PAMPs) or components of injured cells (risk-related molecular patterns or damage-associated molecular patterns (DAMPs)). The CD24Fc can bind to DAMP to capture inflammatory stimulation, prevent its interaction with TLR receptors, and ameliorate excessive inflammation caused by tissue damage. Also, CD24Fc can also bind to another pattern recognition receptor Siglec G/10, which negatively regulates the activity of NF-κB, thereby reducing tissue damage (Clinical trials identifier: NCT04317040). Besides, the activation of many receptors of cytokines can activate downstream Janus kinase (JAK) family kinases. In a case report, three of four critically ill patients using JAK inhibitor ruxolitinib were out of danger, while one patient died [202]. Lymphocyte depletion is a sign of severe exacerbation of COVID-19. Thus, immunomodulators that can reverse the loss of lymphocytes are considered to have therapeutic potential in critical cases. The complement protein C5 inhibitor eculizumab can effectively block the cleavage of C5, thereby inhibiting the production of pro-inflammatory complement components C5a and C5b-9 [203] and preventing neutrophil recruitment and the formation of lytic membrane attack complex (C5b-9), which promotes inflammation and tissue damage [204], thus reversing the loss of T lymphocytes. In addition, BDB-001 is also a monoclonal antibody that antagonizes C5a complement and is currently in clinical trials. The failure of CD8 + T cells expressing inhibitory receptor programmed cell death 1 (PD-1), promotes the persistence of some viruses [205]. Also, the blockade of PD-1 signaling improves the proliferation of specific T lymphocytes *in vitro*. PD-1 monoclonal antibody treatment of COVID-19 is also in clinical trials. The activation of T lymphocytes also depends on the C3 receptor [206] and C-C chemokine receptor type 5 (CCR5) on the cell surface. Therefore, C3 antagonists also have therapeutic potential in CS.

Sphingosine-1-phosphate receptor 1 (S1P receptor) is widely distributed on the surface of vascular endothelial cells and lymphocytes, and is related to CS. Evidence shows that S1P receptor inhibitors are still effective against CS in lymphocyte-deficient Rag2-/- mice [207]. This is mainly related to S1P1 receptor inhibitors that can reduce the capillary leakage caused by CS. Artificial intelligence predicts that drugs related to AP2-related protein kinase 1 (AAK1) can destroy these proteins, thereby inhibiting the virus from entering target cells, indicating that AAK1 inhibitor baricitinib is also expected to be a potential drug [132]. In theory, anti-inflammatory drugs aiming at targets such as dihydroorotate dehydrogenase (DHODH), nucleotide-binding domain leucine-rich repeat and pyrin domain containing receptor 3 (NLRP3), Janus kinase (JAK), and Bruton's tyrosine kinase (BTK) [208] can also be used to regulate tissue damage and excessive inflammation caused by COVID-19. mTOR inhibitors can selectively inhibit the activation of memory B cells, preventing antibody-dependent enhancement, thereby slowing down CS to reduce the severity of COVID-19 [209]. At present, these therapeutic options have entered the clinical trial stage. When the disease is progressively worse, the CS can generate an uncontrolled systemic inflammatory response, which further aggravates the severity of the disease and becomes the main cause of patient death, together with ARDS [130]. Therefore, the application of inflammatory cytokine antagonists in the early stages of progressive diseases may benefit in prognosis.

### 2.3 Treatment of ARDS

Cytokine storm is an important cause of ARDS and the main cause of death of patients with COVID-19. Diffuse alveolar injury is the histological feature of ARDS, with rapid development of capillary congestion, atelectasis, intra-alveolar hemorrhage, and alveolar edema, followed by hyaline membrane formation, epithelial cell proliferation, and interstitial edema in the following days [210]. The pathogenesis of ARDS is divided into three stages, corresponding to histological characteristics. The first stage is ARDS exudative stage characterized by innate immune cell-mediated alveolar endothelial and epithelial barrier damage. In this stage, a large number of pro-inflammatory cytokines are produced, leading to the recruitment of neutrophils and monocytes or macrophages and the activation of effector T cells, thereby maintaining and promoting inflammation to continuous tissue damage [211]. In this process, endothelial activation and microvascular damage further promote and worsen tissue damage [212]. The second stage is the proliferative stage. In this stage, the tissue damage repair begins, the integrity of the epithelium is restored, alveolar edema is absorbed, and alveolar structure and function are restored [213]. This stage is critical to the survival of the host. The last stage is fibrosis, which is not present in all patients and is closely related to the duration of mechanical ventilation.

For the first stage of ARDS exudation, it is particularly important to control and prevent CS and excessive immune response. In addition, reducing endothelial cell damage and improving alveolar edema are also the treatment directions during this period. Angiotensin-converting enzyme (ACE) and ACE2 exist in the epithelium of the respiratory tract and have antagonistic physiological functions [214]. ACE cleaves angiotensin I into angiotensin II, which in turn binds to and activates type 1 angiotensin II receptor (angiotensin type 1 receptor, AT1), and hence exhibits vasoconstriction, pro-inflammatory, and pro-oxidant effects. On the contrary, ACE2 also degrades angiotensin II into angiotensin 1-7 (angiotensin peptide 1-7) and degrades angiotensin I into angiotensin 1-9. When angiotensin 1-9 binds to the Mas receptor, it exerts anti-inflammatory, antioxidant, and vasodilator effects. When the virus binds to ACE2, it reduces the activity of ACE2 so that the balance between ACE/ACE2 is biased toward the angiotensin II enhancement state [17], thus intensifying tissue damage. Therefore, the application of ACE inhibitors and angiotensin peptide 1-7 may benefit; currently, captopril, ramipril, losartan, and angiotensin peptide 1-7 have entered the clinical practice. The inhalation of nitric oxide can inhibit neutrophil migration and oxidative activity, thereby reducing endothelial damage [215]. Vascular endothelial growth factor (VEGF) is the most effective inducing factor to increase vascular permeability. Bevacizumab can antagonize VEGF, thereby reducing the increase in vascular permeability, which has also been exploratorily applied in clinical practice [216]. Drugs that achieve the function of reducing edema through different targets are also in clinical trials. In the exudative phase of ARDS, TNF-mediated tissue factor expression promotes platelet aggregation and microthrombosis, as well as coagulation and hyaline membrane formation in the alveoli [217]. Blocking the internal coagulation process and preventing the formation of microthrombus can also become therapeutic targets in this period. Activated FXIIa catalyzes FXI to produce coagulation factor XII (FXII), which initiates the activation of the internal coagulation pathway. Targeting FXII can prevent thrombosis and does not interfere with hemostasis [218]. Garadacimab is a drug candidate for treatment during this period. In addition, iloprost as prostacyclin protects endothelial damage and has the function of regulating blood clotting.

The second stage of ARDS proliferation is the key recovery period for the host. This stage is characterized by the temporary expansion of resident fibroblasts and the formation of a temporary matrix [217]. Promoting endothelial repair and preventing fibrosis are the key strategies during this period. Oxidative stress caused by harmful reactive oxygen species (ROS) may directly or indirectly participate in human lung fibrosis [219]. NF-E2-related factor 2 (NRF2) is a transcriptional activator of antioxidant/defense genes mediated by antioxidant response elements. The activation of Nrf2 can increase the level of glutathione-S-transferase (GST), NADP(H): quinone oxidoreductase 1 (NQO1), thioredoxin, glutathione peroxidase (GPx), and other antioxidant enzymes, thereby decreasing ROS and reducing pulmonary fibrosis [220]. A variety of drugs in the research and development for anti-fibrosis treatment have entered clinical trials.

The treatment with mesenchymal stem cells (MSCs) has been the research hotspot of ARDS immunotherapy in recent years [221]. MSCs exert anti-inflammatory protective effects on host tissues through the potential anti-inflammatory paracrine factors IL-1ra, TNF-α-stimulated gene 6 protein (TSG-6), insulin-like-growth factor 1 (IGF-1), and prostaglandin E2 (PEG 2) pathway [222]. Meanwhile, MSCs can maintain epithelial cell function [223] and increase the clearance rate of alveolar fluid [224]. MCSs have currently been regarded as a treatment option for patients with severe COVID-19 and are practiced in compassionate use [175, 225, 226] or in clinical trials. Thymosin has shown dual immunoregulatory functions; it is widely used in infectious diseases such as hepatitis B, hepatitis C, sepsis, and aspergillosis [227], as well as in severe COVID-19 [228]. Pulmonary hypertension is a characteristic of ARDS. The dynamic observation of patients with ARDS has revealed that sildenafil can improve pulmonary hypertension [229] but may also lead to the deterioration of oxygenation [229, 230]. However, the application of sildenafil in treating ARDS caused by COVID-19 remains controversial. In addition, immunomodulators such as thalidomide, fingolimod, and aviptadil; immune-stimulants such as GM-CSF; amniotic fluid; as well as anti-inflammatory agents such as tradipitant, colchicine, and losartan are already in clinical trials for treating COVID-19.

### 2.4 Coagulation disorders in COVID-19

Disseminated intravascular coagulation (DIC) is a secondary syndrome characterized by intravascular coagulation caused by other causes of local damage. It manifests as coagulation failure and the intermediate link to develop into multiple-organ failure (MOF) [231]. The initial coagulopathy of COVID-19 is manifested by a significant increase in the levels of D-dimer and fibrin/fibrinogen degradation products [232]. CS can activate the coagulation pathway, leading to the excessive consumption of coagulation factors and platelets [192]. The excessive consumption of coagulation substrates causes coagulation dysfunction, forming a vicious circle and hence leading to DIC. Therefore, D-dimer antagonists and plasminogen activators may be used to prevent the deterioration of coagulopathy and the development of DIC. At present, these anticoagulant drugs have entered the clinical trial stage. China has issued the “Expert consensus for diagnosis and treatment of coagulation dysfunction in COVID-19,” recommending that patients with severe COVID-19 combined with coagulation dysfunction undergo anticoagulation treatment with unfractionated heparin or low-molecular-weight heparin [231]. If patients with severe COVID-19 develop heparin-induced thrombocytopenia, the anticoagulant argatroban/bivalirudin must be replaced. If continuous renal replacement therapy (CRRT) is required for severe COVID-19 with active bleeding, local citrate anticoagulation is recommended.

### 2.5 Vaccines

Vaccines need to be urgently developed as the epidemic spreads and the situation continues to escalate. From the long discovery process, traditional vaccine development may take 15 years or more. Vaccines will be designed, and preclinical experiments will be conducted in this stage. The development of the SARS-CoV-2 vaccine has shortened the discovery process due to the knowledge and experience gained from the development of SARS-CoV and MERS-CoV vaccines, as well as the emergency situation based on the global pandemic. The process is simplified as the interim analysis of Phase I/II results, and then the Phase III trial is directly started. At the same time, vaccine manufacturers have begun the mass production of several risky vaccine candidates. According to the information released by the WHO, research teams from companies and universities around the world are developing more than 100 vaccines against SARS-CoV-2. These vaccines are in different stages of development: some are being tested in animals, while some have reached the clinical stage. The mainstream promising vaccines are listed in Supplementary Table 1. To date, seven main types of vaccine candidates are in the clinical stage: inactivated vaccines, subunit vaccines, adenovirus vector vaccines, lentiviral vector vaccines, measles virus vector vaccines, DNA vaccines, and mRNA vaccines. Inactivated and subunit vaccines are virus-based vaccines made from the killed virus or the fragment of the virus. In China, two inactivated vaccines BBIBP-CorV [161] and CoronaVac [160] developed by Sinopharm and Sinovac Biotech, respectively, are already in clinical Phase III. Adenovirus vector, lentiviral vector, and measles virus vector vaccines are viral vector-based vaccines divided into two types according to the technology: replicating viral vector and nonreplicating viral vector vaccines. ChAdOx1 nCov-2019 (Clinical Trial Identifier: ISRCTN89951424) is a recombinant adenovirus vaccine produced by the cooperation between Oxford University and AstraZeneca. The preliminary results showed an average efficacy of 70% in preventing COVID-19. The vaccine can be stored at normal refrigerated conditions (2-8 °C) (www.astrazeneca.com/media-centre/press-releases/2020/azd1222hlr.html). Ad5-nCoV is another adenovirus vector vaccine developed by CanSino Biologics Inc./Academy of Military Medical Sciences from China. Johnson & Johnson’s JNJ-78436735 vaccine [233] is based on nonreplicating viral vector vaccine technology. DNA- and RNA-based vaccines are nucleic acid vaccines that only need genetic materials to produce the vaccines. Technically, this is the easiest vaccine production strategy. MRT5500 [167] is an mRNA vaccine produced by Sanofi and Translate Bio. Recently, the US pharmaceutical company Pfizer and the German pharmaceutical company BioNTech announced their initial research data on mRNA-based vaccine candidate BNT162b2 [168], claiming that the vaccine jointly developed by the two companies is as efficient as 90%, which is much higher than the minimum requirement of the FDA (50%). Shortly afterward, the first interim analysis of Moderna’s COVID-19 vaccine candidate (mRNA-1273) was published [234]. The result met the primary efficacy endpoint with an efficacy of 94.5% (*P* < 0.0001) (https://investors.modernatx.com/node/10316/pdf). All current vaccines in the clinical stage can induce neutralizing antibodies against viral spike (S) proteins, thereby preventing the virus entry into host cells through the human ACE2 receptor. Once the safety and effectiveness of vaccine candidates are confirmed, COVAX (led by the WHO, Gavi, and Coalition for Epidemic Preparedness Innovations (CEPI)) will promote the fair access and distribution of these vaccines to protect people in all countries.

**Table 3:** Prescription for COVID-19 recommended by Chinese medicine.

Clinical stage		Syndrome	Symptom	Formula	Ref.
Observation period			Fatigue with gastrointestinal discomfort	*Huoxiang Zhengqi* (HXZQ) capsule (pill, liquid or oral solution)	[102, 238]
			Fatigue with fever	*Jinhua Qinggan* granules; *Lianhua Qingwen* capsule (or granules); *Shufeng Jiedu* capsule (or granules); *Fang Feng Tong Sheng* pill (or granules)	[102, 238]
Treatment period	Mild cases	*Cold-damp* invading the lung	Aversion to cold with fever or no fever, muscle aches or headaches, dry throat with cough, tiredness and fatigue, stuffy feeling in chest and stomach, or vomiting, loose stools, light or red tongue, white and greasy fur, tight, wiry or moisten pulse i	Rhizoma *Atractylodes* 15g, dried Pericarpium *Citri Reticulatae* 10g, Cortex *Magnoliae Officinalis* 10g, Chinese *Patchouli* 10g, Fructus *Amomi* 6g, raw Herba *Ephedrae* 6g, Rhizoma et Radix *Notopterygii* 10g, Rhizoma *Zingiberis Recens* 10g, Semen *Arecae* 10g	[238]
				Raw Herba *Ephedrae* 6g, *Gypsum Fibrosum* 15g, Semen *Armeniacae Amarum* 9g, Rhizoma et Radix *Notopterygii* 15g, Semen *Lepidii* 15g, Rhizoma *Cyrtomii* 9g, earthworm *Pheretima* 15g, Radix *Cynanchi Paniculati* 15g, Chinese *Patchouli* 15g, Herba *Eupatorii* 9g, Rhizome *Atratylodes* 15g, *Poria Cocos* 45g, raw white *Atractylodes* Rhizome 30g, charred triplet (charred malt, charred hawthorn, charred medicated leaven) 9g for each, Cortex *Magnoliae Officinalis* 15g, charred Semen *Arecae* 9g, roasted Fructus *Amomi* 9g, Rhizoma *Zingiberis Recens* 15g	[102]
		*Damp-heat* obstructing the lung	Low or no fever, slight aversion to cold, fatigue, heavy head and body feeling, muscular soreness, dry cough with less sputum, sore throat, dry mouth without desire of drinking, or accompanied by stuffy feeling in chest and stomach, no sweat or sweating is not smooth, with a disgusted and dull expression, laxness or loose stools, reddish tongue with thick and greasy or light yellow fur, rolling, rapid or moisten pulse	Semen *Arecae* 10g, Fructus *Amomi* 10g, Cortex *Magnoliae Officinalis* 10g, Rhizoma *Anemarrhenae* 10g, Radix *Scutellariae* 10g, Radix *Bupleuri* 10g, Radix *Paeoniae Rubra* 10g, Fructus *Forsythiae* 15g, Herba *Artemisiae Annuae* 10g (decocted later), Rhizoma *Atractylodes* 10g, Folium *Isatidis* 10g, raw Radix *Glycyrrhizae* 5g	[102]
				Chinese *Patchouli* 10g (decocted later), Cortex *Magnoliae Officinalis* 10g, Rhizoma *Pinellinae Praeparata* 10g, *Poria Cocos* 15g, Radix *Bupleuri* 15g, Radix *Scutellariae* 10g, Radix *Codonopsis* 10g, Semen *Armeniacae Amarum* 10g, Semen *Coicis* 20g, Agar 10g, Rhizoma *Alismatis* 10g, Fructus *Amomi Rotundu* 10g (decocted later), Semen *Sojae Preparatum* 10g, Medulla *Tetrapanacis* 10g, Rhizoma *Zingiberis Recens* 5g, Fructus *Jujubae* 5gg	[243]
	Moderate cases	Toxic *damp* depressing the lung	Low to medium fever, which is obvious in the afternoon, slight aversion to cold, dry cough with less sputum, dry and sore throat, light tongue with yellow greasy fur, rolling and rapid pulse	Folium *Isatidis* 15g, Radix *Scrophulariae* 20g, Radix *Bupleuri* 15g, Radix *Scutellariae* 15g, Rhizoma *Pinellinae Praeparata* 10g, Fructus *Arctii* 10g, Fructus *Forsythiae* 10g, Herba *Taraxaci* 20g, Rhizoma *Cyrtomii* 10g, Herba *Schizonepetae* 10g, raw Radix *Glycyrrhizae* 10g, Radix *Isatidis* 10g, *Poria Cocos* 15g, Semen *Armeniacae Amarum* 12g	[238]
				Raw Herba *Ephedrae* 6g, Semen *Armeniacae Amarae* 15g, *Gypsum Fibrosum* 30g, raw Semen *Coicis* 30g, Rhizoma *Atractylodes* 10g, Chinese *Patchouli* 15g, Herba *Artemisiae Annuae* 12g, Rhizoma *Polygontum Cuspidatum* 20g, Herba *Verbenae* 30g, dried Rhizoma *Phragmitis* 30g, Semen *Lepidii* 15g, Exocarpium *Citri Grandis* 15g, raw Radix *Glycyrrhizae* 10g	[102]
		*Cold-damp* obstructing the lung	Hidden low fever, dry cough with less sputum, tiredness and fatigue, stuffy feeling in chest and stomach, or vomiting, and loose stools, pale or light red tongue with white or greasy fur, moisten pulse	Rhizoma *Atractylodes* 15g, dried Pericarpium *Citri Reticulatae* 10g, Cortex *Magnoliae Officinalis* 10g, Chinese *Patchouli* 10g, Fructus *Amomi* 6g, raw *Ephedrae* 6g, Rhizoma et Radix *Notopterygii* 10g, Rhizoma *Zingiberis Recens* 10g, Semen *Arecae* 10g	[102]
		Toxic *damp* accumulated in interior, involving lung and spleen	Low fever, which is obvious in the afternoon, dry cough with less sputum, dry and sore throat, stuffy feeling in chest and stomach, vomiting and anorexia, laxness and fatigue, pale tongue with greasy fur, rolling and rapid pulse	Folium *Isatidis* 15g, Radix *Scrophulariae* 20g, Radix *Bupleuri* 15g, Radix *Scutellariae* 15g, Rhizoma *Pinellinae Praeparata* 10g, Fructus *Arctii* 10g, Fructus *Forsythiae* 10g, Herba *Taraxaci* 20g, Rhizoma *Cyrtomii* 10g, Herba *Schizonepetae* 10g, raw Radix *Glycyrrhizae* 10g, *Poria Cocos* 15g, Semen *Armeniacae Amarum*12g, Radix *Angelica Dahurica* 10g, Chinese *Patchouli* 10g, Herba *Eupatorii* 10g	[238]
		Toxic *damp* obstructing the spleen	Mental depression, fatigue, weakness, anorexia, loose stools, borborygmus, abdominal fullness, chest tightness and shortness of breath, pale tongue with greasy fur, soft and moisten pulse	Chinese *Patchouli* 15g, Radix *Angelica Dahurica* 10g, Folium *Perillae* 6g, *Poria Cocos* 30g, Rhizoma *Pinelliae* processed by ginger juice 12g, Rhizoma *Atractylodes* 10g, white *Atractylodes* Rhizome 15g, dried Pericarpium *Citri Reticulatae* 10g, Cortex *Magnoliae Officinalis* 10g, Radix *Glycyrrhiza Preparata* 6g, Fructus *Amomi Rotundu* 10g, Radix *Platycodonis* 10g	[238]
		*Evil heat* obstructing the lung	Fever or high fever, cough, yellow or thick phlegm, fatigue, headache, muscular stiffness, dry and bitter mouth, anxiety, red urinary and constipation, red tongue with yellow or greasy fur, rolling and rapid pulse	Fried Herba *Ephedrae* 8g, Semen *Armeniacae Amarum* 10g, *Gypsum Fibrosum* 30g, Radix *Glycyrrhizae* 10g, Semen *Arecae* 10g, Cortex *Magnoliae Officinalis* 10g, Fructus *Amomi* 10g, Rhizoma *Anemarrhenae* 10g, Radix *Paeoniae Alba* 10g, Radix *Scutellariae* 15g	[243]
	Severe cases	Toxic plague blocking the lung	Chest tightness, shortness of breath, coughing and panting, extreme fatigue, asthmatic, cough with less sputum or hemoptysis or yellow sputum, thirsty and irritable, high fever does not retreat, bloating and constipation, dark red tongue with yellow greasy fur, slippery or heavy pulse	Semen *Armeniacae Amarum* 10g, *Gypsum Fibrosum* 30g, Pericarpium *Trichosanthis* or Semen *Trichosanthis* 30g, raw Radix et Rhizoma *Rhei* 6g (decocted later), fried Herba *Ephedrae* 6g, Semen *Lepidii* 10g, Semen *Persicae* 10g, Fructus *Amomi* 6g, Semen *Arecae* 10g, Rhizoma *Atractylodes* 10g	[238]
				Semen *Armeniacae Amarum* 15g, *Gypsum Fibrosum* 30g (chopped), Fructus *Trichosanthis* 30g, Fructus *Aurantii Immaturus* 15g, raw Radix et Rhizoma *Rhei* 15g (decocted later), raw Herba *Ephedrae* 6~10g, Semen *Lepidii* 30g, Semen *Persicae* 10g, Radix *Paeoniae Rubra* 15g, raw Radix *Glycyrrhizae* 6g, Rhizoma *Phragmitis* 30g	[238]
				Raw Herba *Ephedrae* 6g, Semen *Armeniacae Amarum* 9g, *Gypsum Fibrosum* 15g, Radix *Glycyrrhizae* 3g, Chinese *Patchouli* 10g (decocted later), Cortex *Magnoliae Officinalis* 10g, Rhizoma *Atractylodes* 15g, Fructus *Amomi* 10g, Rhizoma *Pinellinae Praeparata* 9g, *Poria Cocos* 15g, raw Radix et Rhizoma *Rhei* 5g (decocted later), raw Radix *Astragali* 10g, Semen *Lepidii* 10g, Radix *Paeoniae Rubra* 10g	[102]
			Fever, cough, thick yellow phlegm, chest tightness, wheezing, thirst, stinking tone, bloating and constipation, dark red tongue with thick and yellow fur, slippery or tight pulse	Raw Herba *Ephedrae* 8g, Semen *Armeniacae Amarum* 12g, *Gypsum Fibrosum* 30g, raw Radix et Rhizoma *Rhei* 10g, Semen *Trichosanthis* 30g, Semen *Persicae* 10g, Radix *Paeoniae Rubra* 15g, Semen *Lepidii* 20g, Rhizoma *Coptidis* 3g, Radix *Scutellariae* 10g, Cortex *Mori* 10g, Rhizoma *Paridis* 10g, Cortex *Mouta*n 15g, Radix *Curcumae* 15g, Rhizoma *Acori Graminei* 15g, raw Radix *Rehmanniae* 15g, Radix *Scrophulariae* 15g	[243]
			Hidden fever, disturbed hidrosis, panting, dry coughing or bucking, or with sore throat, stuffy feeling in chest and stomach, dry mouth, bitter or sticky in the mouth, sticky stools, dark red tongue with yellow and greasy fur, slippery pulse	Raw Herba *Ephedrae* 8g, Semen *Armeniacae Amarum* 12g, *Gypsum Fibrosum* 30g, raw Radix *Glycyrrhizae* 10g, Talcum powder 30g, Herba *Artemisiae Scopariae* 20g, Radix *Scutellariae* 15g, Fructus *Amomi Rotundu* 10g (decocted later), Chinese *Patchouli* 15g, Rhizoma *Pinellinae Praeparata* 15g, Rhizoma *Atractylodes* 15g, Semen *Lepidii* 20g, Fructus *Forsythiae* 15g, *Bombyx Batryticatus* 5g, *Periostracum Cicadae* 5g, Rhizoma *Curcumae Longae* 10g, raw Radix et Rhizoma *Rhei* 5g, Rhizoma *Paridis* 10g, Cortex *Moutan* 15g, Radix *Paeoniae Rubra* 15g, Radix *Curcumae* 15g, Rhizoma *Acori Graminei* 15g, raw Radix *Rehmanniae* 15g, Radix *Scrophulariae* 15g	[243]
				raw Herba *Ephedrae* 8g, Semen *Armeniacae Amarum* 12g, *Gypsum Fibrosum* 30g, raw Radix *Glycyrrhizae* 10g, Talcum powder 30g, Herba *Artemisiae Scopariae* 20g, Radix *Scutellariae* 15g, Fructus *Amomi Rotundu* 10g (decocted later), Chinese *Patchouli* 15g, Rhizoma *Pinellinae Praeparata* 15g, Rhizoma *Atractylodes* 15g, Semen *Lepidii* 20g, Fructus *Forsythiae* 15g, *Bombyx Batryticatus* 5g, *Periostracum Cicadae* 5g, Rhizoma *Curcumae Longae* 10g, raw Radix et Rhizoma *Rhei* 5g, Rhizoma *Paridis* 10g, Cortex *Moutan* 15g, Radix *Paeoniae Rubra* 15g, Radix *Curcumae* 15g, Rhizoma *Acori Graminei* 15g, raw Radix *Rehmanniae* 15g, Radix *Scrophulariae* 15g	[243]
		Blazing heat in *qi* and *ying fen*	Hot and thirsty, panting, dizzy delirium, blurred vision with macula, hematemesis and epistaxis, convulsions in the limbs, crimson tongue with little or no fur, deep, thin and rapid pulse, or floating and rapid pulse	*Gypsum Fibrosum* 30~60g (decocted first), Rhizoma *Anemarrhenae* 30g, Radix *Rehmanniae Preparata* 30~60g, Cornu *Bubali* 30g (decocted first), Radix *Paeoniae Rubra* 30g, Radix *Scrophulariae* 30g, Fructus *Forsythiae* 15g, Cortex *Moutan* 15g, Rhizoma *Coptidis* 6g, Folium *Phyllostachys* 12g, Semen *Lepidii* 15g, raw Radix *Glycyrrhizae* 6g	[102]
	Critical cases	Inner blocking causing collapse	Dyspnea, asthmatic, or assisted ventilation needed, with dizziness and irritability, sweating, cold limbs, dark purple tongue with thick, greasy or dry fur, large floating and weak pulse	Radix *Ginseng* 15g, Radix *Aconiti Lateralis Preparata* 10g (decocted first), Fructus *Corni* 15g; with *Suhexiang* pill or *Angong Niuhuang* pill; high fever plus *Zixue* powder; severe breathing plus Semen *Lepidii* and Herba *Ephedrae*	[238]
				Radix *Panacis Quinquefolii* 15g, Radix *Aconiti Lateralis Preparata* 10g, Fructus *Corni* 30g, crude *Os Draconis* 30g, Concha *Ostreae* 30g, *Magnetitum* 30g; with *Angong Niuhuang* pill	[238]
				Radix *Ginseng* 15g, Radix *Aconiti Lateralis Preparata* 10g (decocted first), Fructus *Corni* 15g; with *Suhexiang* pill or *Angong Niuhuang* pill	[102]
			High fever and irritability, cough and shortness of breath, the nose wings incite, phlegm in the throat, holding breath in embarrassment, intermittent voice, spots of rash, or even dizzy coma, sweating, cold limbs, dark purple lips, dark red tongue with yellow and greasy fur, deep, thin and delicate pulse	Radix *Ginseng Rubra* 10g, Radix *Aconiti Lateralis Preparata* 10g (decocted first), Fructus *Corni* 30g, Radix *Ophiopogonis* 20g, Radix *Notoginseng* 10g	[243]
Recovery period		*Qi* deficiency of lung and spleen	Shortness of breath, fatigue, anorexia, vomiting, fullness, weak and loose stools, pale fat tongue with white and greasy fur.	Rhizoma *Pinellinae Praeparata* 9g, dried Pericarpium *Citri Reticulatae* 10g, Radix *Codonopsis* 15g, Radix *Astragali Preparata* 30g, *Poria Cocos* 15g, Chinese *Patchouli* 10g, Fructus *Amomi* 6g (decocted later)	[102, 238]
				Dried Radix *Ginseng* 10g (boiled separately), fried Rhizoma *Atractylodis Macrocephalae* 15g, *Poria Cocos* 15g, Semen *Lablab Album* 30g, Fructus *Amomi* 6g (chopped, decocted later), Semen *Nelumbinis* 30g, Radix *Glycyrrhiza Preparata* 6g, Radix *Platycodonis* 10g, Rhizoma *Dioscoreae* 15g, Semen *Coicis* 30g, fried Fructus *Hordei Germinatus* 30g, medicated leaven 10g	[243]
		Deficiency of both *qi* and *yin*	Shortness of breath, fatigue, coughing, less phlegm, low or no fever, thirst, anorexia, dry and dark tongue with dry and white fur, thin and weak pulse	Radix *Adenophorae* 10g, Radix *Glehniae* 10g, Radix *Ophiopogonis* 6g, Fructus *Schisandrae* 10g, *Gypsum Fibrosum* 15g, Herba *Lophatheri* 10g, Folium *Mori* 6g, Rhizoma *Phragmitis* 15g, Radix *Trichosanthis* 15g, *Poria Cocos* 10g, Radix *Salviae Miltiorrhizae* 15g, Semen *Persicae* 10g, Radix *Glycyrrhizae* 6g	[102, 238]
				*Buzhong Yiqi* pill; *Shengmai* drink; *Xiangsha Liujunzi* pill; *Shen Ling Baizhu* powder	[238]
				Radix *Panacis Quinquefolii* 20g, Herba *Dendrobii* 10g, Radix *Ophiopogonis* 10g, Rhizoma *Anemarrhenae* 10g, Herba *Lophatheri* 10g, Rhizoma *Coptidis* 3g, Radix *Glycyrrhizae* 6g, *Poria Cocos* 15g, Rhizoma *Pinellinae Praeparata* 10g, Exocarpium *Citri Rubrum* 10g, dried Pericarpium *Citri Reticulatae* 10g, fried Fructus *Hordei Germinatus* 30g	[243]
Prevention	High-risk group			Radix *Astragali* 15g, fried Rhizoma *Atractylodes* 10g, Radix *Sileris* 10g, Flos *Lonicerae* 10g, Rhizoma *Dryopteris Crassirhizomatis* 10g, dried Pericarpium *Citri Reticulatae* 10g, Semen *Lablab Album* 15g, *Poria Cocos* 10g	[238]
				Raw Radix *Astragali* 10g, fried white *Atractylodes* Rhizome 10g, Radix *Sileris* 6g, Flos *Lonicerae* 10g, Herba *Eupatorii* 10g, dried Pericarpium *Citri Reticulatae* 10g, Chinese *Patchouli* 10g, *Poria Cocos* 10g, raw Radix *Glycyrrhizae* 6g	[238]
	Healthy group			Roasted Rhizoma *Atractylodes* 3g, Flos *Lonicerae* 3g, dried Pericarpium *Citri Reticulatae* 3g, Rhizoma *Phragmitis* 6g, Folium *Mori* 3g, Folium *Perillae* 3g, raw Radix *Astragali* 6g	[238]
				Radix *Codonopsis* 20g, Folium *Perillae* 10g, Herba *Schizonepetae* 10g, Radix *Sileris* 10g	[243]
				Rhizoma *Zingiberis Recens* 10g, Fructus *Jujubae* 15g, Radix *Sileris* 10g	[243]

## 3. Understanding COVID-19 From the Perspective of TCM

Currently, numerous drug candidates for COVID-19 are in clinical trials; finding a specific drug may still take time. On May 1, the FDA issued an EUA that authorized the use of remdesivir to treat suspected or laboratory-confirmed adult and pediatric patients with severe COVID-19 [235]. So far, the information on the safety and efficacy of remdesivir in treating COVID-19 remains incomplete. Gilead Sciences Inc. (GILD) announced the results of the NCT04280705 trial, which recruited 1062 patients, and showed a significant reduction in the clinical recovery time of 31% (11 days vs 15 days, *P* < 0.001) in the remdesivir group compared with the placebo group, with no significant reduction in mortality (8% vs 11%, *P* = 0.059) [236]. The efficacy of remdesivir against COVID-19 was not demonstrated in a previous clinical trial involving 53 patients [88]. In a randomized, double-blind, placebo-controlled trial with severe COVID-19, the administration of remdesivir was not associated with the duration of clinical improvement. However, among patients with symptoms lasting 10 days or less, the duration of clinical improvement was significantly shorter in the remdesivir group than in the placebo group [237]. In the early stage of the epidemic, lopinavir/ritonavir, which was a promising drug combination for treating COVID-19, showed no clinical benefit in 99 severe cases [171]. Still, no effective antiviral drugs are available for severe cases, while clinical treatments mainly focus on the prevention and treatment of symptoms and complications. On the contrary, some traditional medicine practices, such as TCM, have shown certain group efficacy and advantages in this large-scale epidemic.

In the TCM theory, COVID-19 belongs to the category of blight (yì bìng), caused by plague, which refers to dampness (shī dú zhī xié). The characteristics of the disease are damp-heat and turbidity (shī rè zhuó dú), located in the lung, often involving the spleen, stomach, and large intestine [238]. "The Plague Theory (wēnyì lùn)" by Wu Youke (1582-1652 C.E.) in Ming Dynasty pointed out that the infection mode of external dampness was "the evil of the epidemic comes from the mouth and nose." The basic pathogenesis is exogenous dampness, hurting the lung guard; transforming into heat to enter deeper in the body, thereby consuming *qi* and hurting *yin*; and invading the viscera. The blood is transformed into sediment and transmitted directly to the pericardium quickly with the poisonous heat intertwining. This dampness eventually develops into a critical illness. In addition, the pathological characteristics of COVID-19 are "wet, blood stasis, poison, and deficiency" [239].

Ever since ancient times, TCM has been successful in treating plague. The treatment of plague is not only against the virus but also for the overall adjustment of the relationship between the *upright* (zhèng) and the *evil* ( xié) after the disease has invaded the body. "Book of Jin (jìn shū)" in the Tang Dynasty (completed in 648 AD) also has a record of "the *upright* stays inside, and the *evil* cannot interfere" [240]. According to "The Plague Theory," whether the dampness develops into disease after invading the human body depends on the ups and downs of the *upright qi* (zhèng qì) and the severity of the *evil qi*. The dampness is one type of *evil qi*. *Upright qi* is the opposite of *evil qi* (xié qì), which refers to a class of fine substances in the body that have the functions of disease resistance, evil elimination, regulation, repair, and so forth [241]. The theory of *upright qi* and *evil qi* has provided a reasonable explanation for asymptomatic infected persons and the virus transmission caused by asymptomatic infected persons [25]. "Those with strong *upright qi* can't be infected even though they face strong *evil qi*." That is, the body's strong immunity is not easy to be attacked by external *evil qi*, thus equating *upright qi* to a certain extent with nonspecific immunity [242]. As mentioned earlier, "The *upright* stays inside, and the *evil* cannot interfere." However, "when *upright qi* coincides with the loss, external *evil qi* would take advantage of it between breathing." And "if the plague prevails in this year, and the patients will be severe, the disease will be most contagious " [240], indicating that the epidemic goes into a vicious circle without a deep understanding of the etiology. As TCM emphasizes that the treatment of epidemic diseases needs to not only get rid of *evil qi* but also support *upright qi*, it is evident that some TCM-based treatment concepts for acute infectious diseases are very advanced.

TCM adopts the diagnosis and treatment method combining disease identification and syndrome differentiation, making a more accurate judgment on the condition of the disease. Modern science and technology have promoted the rapid development of modern medicine. In the face of the need to diagnose diseases and improve objective indicators, diseases diagnosed by modern medicine are renamed, classified, and staged according to the TCM theory in this epidemic [102, 238, 244] (Table 3). According to TCM, COVID-19 is caused by dampness involving different geographical locations, climates, customs, habits, physique differences, and so forth. This dampness has changed from cold to heat during the development of the disease. A person with a white face and *yang* (yáng) deficiency easily becomes cold after experiencing dampness or other *evil qi*, thereby developing cold dampness and presenting the syndrome of "*cold-damp* depressing the lung (hán shī yù fèi)." On the contrary, it is easy to heat up and develop heat dampness for a person with a red face and *yin* (yīn) deficiency, thus reflecting the syndrome of "*damp-heat* accumulating in the lung (shī rè yùn fèi)." These are the two main syndromes in the early stage of infection with dampness, which are more common in mild and moderate patients. At this time, the treatment should focus on squandering excessive *qi* and removing *evil qi*. This is also how antiviral drugs (recognized as *qu xie*) have shown a certain effect in the early stage of COVID-19. The *cold-damp* can further develop into a syndrome that not only obstructs the lung but also traps the spleen, showing clinical signs of chest tightness, vomiting, anorexia, and loose stools. In treating diseases in this stage, warm energy is also needed to disperse cold besides demonstrating lung *qi*. If symptoms of toxic dampness appear, it is necessary to strengthen the spleen and clean the lung. At this time, the *damp-heat* syndrome may also form the syndrome of *phlegm-heat* obstructing the lungs during further development; it is necessary to clear the heat and detoxify, as well as expel *evil qi* from the lungs.

**Table 4:** Commercial Chinese medicine preparations and herb supplement in treatment of COVID19.

Treatment	Administration and Dosage	Outcome Measures	Source
Lianhua Qingwen	PO, 4 capsules, tid, 7 d	Virological clearance	NCT04433013 (SG)
Xiyanping injection	IV, 10-20 mL, qd, Combination medication, 14 d	Clinical recovery time	NCT04275388 (CN) NCT04295551 (CN)
Huaier keli	PO, 20 g, 14 d	Mortality	NCT04291053 (CN)
T89 (Danshen Diwan)	PO, 30 pills, bid	Improve oxygen saturation	NCT04285190 (CN)
Fuzheng Huayu Tablet	PO, 1.6 g, tid	Evaluation of Pulmonary fibrosis Improvement.	NCT04279197 (CN)
Conventional medicines (Oxygen therapy, alfa interferon via aerosol inhalation, and lopinavir/ritonavir) and Traditional Chinese Medicines (TCMs) granules	PO, 20 g, bid, 14 d	The incidence rate of ARDS development	NCT04251871 (CN)
Chinese Herbal Medicine	PO	Patient reported main complaint	NCT04380870 (US)
Reginmune	PO, 1000 mg, tid, 10 d	Time to clinical improvement	NCT04494204 (IN)
Licorice extract	PO, 250 mg standardized extract, 10 d	Increased number of recovery from COVID19	NCT04487964 (EG)
Iota-Carrageenan	ISIN, 4 times, 21 d	Change in SARS-CoV-2 antibody titers	NCT04521322 (AR)
çaí palm berry extract	PO, 520 mg, tid, 30 d	7-point ordinal symptom scale	NCT04404218 (CA)
P2Et (Caesalpinia spinosa extract)	PO, 250 mg, bid, 14 d	Proportion of patients who reduce the time in the hospital	NCT04410510 (CO)
Nigella sativa	PO, 500 mg, Black seed oil	Proportion of patients who are clinically recovered	NCT04401202 (SA)
Nigella Sativa / Black Cumin	PO, 80 mg/kg/d, 14 d	Time needed to turn positive COVID-19 PCR to negative	NCT04472585 (PK) NCT04347382
Natural Honey	PO, 30 mL, bid, 14 d	Time needed to turn positive COVID-19 PCR to negative	NCT04323345 (EG) NCT04347382 (PK)
Antioxidation Therapy	Two proprietary formulations composed of reduced glutathione, N-acetylcysteine, supero×ide dismutase, and bovine lactoferrin and immunoglobulins. PO, 28 d	Time to clinical improvement	NCT04466657 (NG)
Aromatherapy	INH, 15 min	Change from baseline in state anxiety on the State portion of the State Trait Anxiety Scale (STAI-S) at 15 minutes	NCT04495842 (US)
Ayurveda	PO	Time to achieve afebrile	NCT04395976 (GB) NCT04345549 (GB) NCT04351542
Ayurvedic Kadha	PO, 30 d	Prevention	NCT04387643 (IN)
Natural Honey	PO, 1 g/kg/d, 14 d	Rate of recovery	NCT04323345 (EG) NCT04347382 (PK)
QuadraMune(TM)	PO, 2 pills, 84 d	Prevention	NCT04421391 (US)
Resveratrol	PO, 15 d	Length of stay in hospital	NCT04400890 (US)
Tetrandrine	PO, 60 mg, qd, 7 d	Survival rate	NCT04308317 (CN)
Quercetin	PO, 500 mg, 90 d	Prevalence of COVID-19	NCT04377789 (TR)
Silymarin	PO, 140 mg, tid, 7-28 d	Time to clinical improvement	NCT04394208 (EG)
Escin	PO, 40 mg, tid, 12 d	Mortality	NCT04322344 (IT)
Pyronaridine-Artesunate	PO, Pyronaridine 180mg/ Artesunate 60mg, 7 d	Virological clearance	NCT04475107 (KR)
Artemisinin / Artesunate	PO, 100 mg, 5 d	Length of stay in hospital	NCT04387240 (SA)
ArtemiC	PO	Time to clinical improvement	NCT04382040 (IL)
Fisetin	PO, 20 mg/kg/d	Serious adverse events and change in oxygenation status	NCT04476953 (US)
LEAF-4L6715	IV, initial dose of 5 mg, 2.5 mg, qd	Improve oxygenation status	NCT04378920 (FR)
LEAF-4L7520	IV, 0.25 mg/kg per 3h	Improve oxygenation status	NCT04378920 (FR)
Manremyc	PO, 1×10^5 heat-inactivated Mycobacterium s. 14 d	Documented cumulative incidence of SARS-CoV-2 infection	NCT04452773 (ES)
Glycine	PO, 0.5 g/kg	Mortality	NCT04443673 (US)
EPA-FFA	PO, 1 g, bid	Rate of treatment failure	NCT04335032 (CH)
omega3-FA	PO, 300 mg, 60 d	Inflammatory factor levels	NCT04483271 (JO)
Vitamins	AZINC forme etvitalité®, 2 tablets, PO, 9 d	Occurrence of hospitalization and mortality	NCT04356495 (FR)
Vitamin D	PO, 1000-500000 UI, 30 d	Mortality	NCT04334005 (ES) NCT04335084 (US) NCT04344041 (FR)
Vitamin D3	PO, 2000-100000 UI, 10-30 d	Severity, mortality and inflammatory factor levels	NCT04351490 (FR) NCT04395768 (AU) NCT04400890 (US)
Vitamin C	IV, 10-12 g or 50-60 mg/kg, 9 d	Mortality and dependency on mechanical ventilation, renal replacement, or vasopressors	NCT03680274 (CN) NCT04323514 (IT) NCT04328961 (US)
	PO, 500-1000 mg	Symptom Severity	NCT04354428 (US) NCT04530539 (US)
Vitamin C +Vitamin D +zinc	PO	Prevention	NCT04335084 (US) NCT04334512 (US)
Vitamin C + Folic Acid	VC: PO, 500 mg, 9 d Folic acid: PO, 400 mcg, 9 d	Incidence of hospitalization or mortality and change in upper respiratory viral shedding	NCT04354428 (US)
Vitamin B	Super B-Comple×, bid, PO, 42 d	Prevention	NCT04343248 (US)
Vitamin B12	Adjuvant therapy: PO, 14 d	Symptoms, length of hospital stay and mortality	NCT04395768 (AU)
Vitamin B3	IV, 20000 UI, 3 d	Change in SARS-CoV-2 antibody titers	NCT04482673 (US)
Zinc gluconate	PO, 15-20 mg, bid, 60 d	Survival rate in asymptomatic subjects at inclusion	NCT04351490 (FR) NCT04447534 (EG) NCT04472585 (PK)
L-citrulline	PO, 7 d	SOFA score for organ failures on D7 or last known SOFA score if the patient has died or been resuscitated	NCT04404426 (FR)
Nutritional support system	Diet based on the Basal Energy E×penditure plus the stress factor using the Harris Benedict equation.	Oxygen saturation	NCT04507867 (MX)
SivoMixx	Composition of SivoMi××: Streptococcus thermophilus DSM322245, Bifidobacterium lactis DSM 32246, Bifidobacterium lactis DSM 32247, Lactobacillus acidophilus DSM 32241, Lactobacillus helveticus DSM 32242, Lactobacillus paracasei DSM 32243, Lactobacillus plantarum DSM 32244, Lactobacillus brevis DSM 27961 (NB: DSM n°... : bacterial strain identification code), 21 d	Delta of time of disappearance of acute diarrhea	NCT04368351 (IT)
Omnibiotic AAD	Bacterial strains in Omni-Biotic® 10 AAD are Bifidobacterium bifidum W23, Bifidobacterium lactis W51, Enterococcus faecium W54, Lactobacillus acidophilus W37, Lactobacillus acidophilus W55, Lactobacillus paracasei W20, Lactobacillus plantarum W1, Lactobacillus plantarum W62, Lactobacillus rhamnosus W71 and Lactobacillus salivarius W24 which are embedded in a matri× containing maize starch, maltode×trin, inulin, potassium chloride, hydrolysed rice protein, magnesium sulphate, fructooligosaccharide (FOS), enzymes (amylases), vanilla flavour and manganese sulphate, 30 d	Delta of time of disappearance of acute diarrhea	NCT04420676 (AT)
MRx-4DP0004	Lyophilised formulation of a proprietary strain of bacteria: 4 × 10^9 to 4 ×10^10 colony forming units, PO, 14 d	Change in mean clinical status score in each treatment arm	NCT04363372 (GB)
Probiotic	PO, 1×10^9 cfu of the probiotic, 30 d	Cases with discharge to ICU	NCT04390477 (ES) NCT04517422
bacTRL-Spike	Single dose of bacTRL-Spike, equivalent to 1-3 billion colony forming units (cfu) of Bifidiobacterium longum; PO	Genetically modified probiotic bacteria colonize the gut, bind directly to intestinal epithelial cells and constitutively replicate, secrete and deliver plasmid DNA molecules encoding antigenic transgenes and neutralizing nanobodies.	NCT04334980 (US, CA)
Previfenon®	PO, 250 mg, tid	Improve symptoms	NCT04446065 (AU)

In COVID-19 disease, cold dampness changes rapidly, with the main event of *yang* injury, combined with heat, dryness, *yin* injury, blood stasis, and atrophy. This means that *cold-damp* can also turn into *damp-heat* during disease development. In late mild cases or early severe cases, common symptoms of toxic plague blocking the lung (yì dú bì fèi) are always observed. *Damp-heat* causes dryness, which damages *yin*. When the disease develops into middle and later stages, many *yin* injuries occur. In severe cases, both *qi* and *yin* are depleted, presenting a dark red tongue with less or peeling fur. Most of the patients who are about to recover from the disease have *qi* deficiency of the lung and spleen, thereby presenting the symptoms of blazing heat in both *qi* and blood. In critical cases, the *yin* and *yang* are almost departed and separated. Due to *qi* desertion, patients are more willing to lie down in low spirits. As the *upright qi* is to take off, the *yin* fluid loses astringency, presenting shortness of breath and sweating. When the *yin* fluid is deficient, it causes sudden *yang* collapse, presenting with cold limbs, severe shortness of breath, dripping cold sweat, and delicate and desperate pulse. In this case, the treatment strategy should be tonifying *qi*, recuperating depleted *yang*, and rescuing the patient from collapse. During the recovery period, the *qi* deficiency of the lung and spleen are observed, which are more common in patients who have been discharged from the hospital after treatment.

Currently, some commercial Chinese medicine preparations are also used in this epidemic. A randomized clinical trial evaluated the clinical efficacy of *Huoxiang Zhengqi* drop pills and/or *Lianhua Qingwen* granules combined with Western medicine in treating COVID-19 [245]. The results showed that *Huoxiang Zhengqi* drop pills combined with *Lianhua Qingwen* granules and Western medicine had advantages in improving nausea, vomiting, and sore limbs, as well as reducing the use of antibiotics. The efficacy evaluation of *Xiyanping* injection (NCT04275388) and *Huai’er* granules for COVID-19 has also entered clinical trials. Herbal extracts, such as licorice, acai palm berry, black locust, and black cumin; star compounds of herbal origin, such as silymarin, artesunate, pyrrolidine-artesunate, tetrandrine, resveratrol, quercetin, vitamins, and folic acid; and even Ayurveda also play a role in adjuvant therapy as supplementary or alternative therapies during treatment. Recently, several observational studies have been carried out worldwide (Table 4).

Although Western medicine-based treatment proposes to release the isolation and discharge standards, it lacks follow-up treatment for these patients. Establishing this period of TCM-based treatment plan can benefit patients in terms of their further recovery. Also, adding corresponding notes can fill the gap in the follow-up treatment of these patients by Western medicine, which has potential significance and unique advantages. So far, TCM-based treatment of COVID-19 can significantly improve the clinical symptoms of patients. Mild and moderate patients are easily cured after treatment with TCM. Meanwhile, the conversion of moderate into severe cases has been significantly reduced. Also, patients in the recovery period benefit from TCM.

## 4. Discussion

The present review outlined the drug development and treatment strategies since the outbreak of SARS-CoV-2, as well as the theory and practice of TCM treatment of COVID-19. The development of Western medicine for COVID-19 is based on the pathogenesis of the disease, involving viral infection, immune suppression induced during the infection process, and CS, ARDS, and DIC caused by excessive inflammation. During TCM treatment, the cause of the disease is examined, the pathogenesis is analyzed, and the measures are adapted to individual conditions. The treatment options of the two medical systems are very different, but similarities exist in the treatment concept. Western medicine has several contradictory drugs, such as the controversial application of glucocorticoids, interferon and interferon antagonist monoclonal antibodies, nonsteroidal anti-inflammatory drugs, anticoagulation and hemostatic therapy, as well as antagonistic GM-CSF monoclonal antibodies and GM-CSF. In the initial stage of infection, blocking the infection and removing the virus are the primary tasks. Besides directly targeting viral invasion and replication, improving innate immunity contributes to clearing the virus in patients with mild COVID-19. The application of interferon is beneficial during this period. In this stage, preventive glucocorticoids can suppress the immune response and delay viral clearance and prolong the course of the disease. Patients with severe and critical COVID-19 also experience CS at the same time. Inhibiting the inflammatory response and preventing the further development of CS are the core tasks of treatment during this period. Therefore, the application of glucocorticoids and interferon antagonism is required.

Taken together, treatment for COVID-19 should be based on the severity of the disease, period of infection, and individual differences, which coincides with the idea of "treatment based on an overall diagnosis" (stages and types of diseases), "prescribing the right medicine," and "appropriate measures to the individual" of TCM theory. From a social perspective, drug development often takes a long time and is costly, making it difficult to respond to the current global outbreak on time. In this case, traditional medicine, as empirical medicine, can make up for the shortcomings of drug treatment as supplementary treatment, adjuvant treatment, and preventive means. Moreover, the feasibility of the combined use of Chinese and Western medicine has been verified in clinical practice. A 58-year-old severely ill female patient received nonselective antiviral Western medicine treatment (ropinavir/ritonavir, arbidol, and methyl-prednisolone) and also a formula including 16 Chinese medicines based on the patient’s symptoms, which showed the therapeutic effect of TCM on the critical COVID-19 [246]. The Shanghai Public Health Clinical Center reported four mild or severe cases given antiviral lopinavir/ritonavir and arbidol treatment combined with a Chinese patent medicine *Shufeng Jiedu* Capsule. Two out of four patients recovered after the treatment, while the other two patients showed significant improvement. [247] In a retrospective clinical study, 63 patients were treated with *Qingfei Paidu* Decoction combined with antiviral drugs such as interferon, lopinavir, or arbidol, leading to obvious anti-inflammatory benefits. Although the mortality rate and duration of hospital stay were not affected, combination therapy tended to reduce multiple-organ damage. [248] In addition, several meta-analyses showed that the combination of TCM, such as *Lianhua Qingwen* decoction [249], *Qingfei Paidu* Decoction [250], and *Reduning* injection [251], with Western medicine achieved certain clinical benefits.

## Supplementary Materials

The Supplemenantry data can be found online at: www.aginganddisease.org/EN/10.14336/AD.2020.1124.


