# Supplementary material for: A Comprehensive Summary of the Knowledge on COVID-19 Treatment
Source: Aging Dis. 2021 Feb 1;12(1):155–91. doi: 10.14336/AD.2020.1124 (PMC7801274; doi:10.14336/AD.2020.1124)
Supplement: Supplementary file 1 [file AD-12-1-155-s.pdf]

SUPPLEMENTARY DATA

# Comprehensive Summary of the Knowledge on COVID-19 Treatment

**Yu Peng[1, #], Hongxun Tao[1,#], Senthil Kumaran Satyanarayanan[1], Kunlin Jin[2,*], Huanxing Su[1,*]**

[1]State Key Laboratory of Quality Research in Chinese Medicine, Institute of Chinese Medical Sciences, University of Macau, Macao, China.
[2]Department of Pharmacology and Neuroscience, University of North Texas Health Science Center, Fort Worth, TX 76107, USA.

© 2020. Peng Y et al. Published online at http://www.aginganddisease.org/EN/10.14336/AD.2020.1124

# SUPPLEMENTARY DATA

**Supplementary Table 1.** Potential drug candidates for the treatment of COVID-19.

| Classification | Drug | Drug target | Dosage | Source | Mechanism | Indications and usage | Ref. |
|---|---|---|---|---|---|---|---|
| **Antiviral therapy** | Camostat Mesilate | Type II transmembrane serine protease (TMPRSS2) inhibitor | PO, 200 mg, tid, 5d | NCT04353284 (US)<br>NCT04455815 (GB)<br>NCT04521296 (JP) | Block the virtual entry. | Treatment of some forms of cancer | [67] |
| | Nafamostat mesilate | TMPRSS2 inhibitor | Intravenous administration, 0.1-0.2 mg/kg/h | NCT04418128 (US)<br>NCT04390594 (SN)<br>NCT04352400 (CH) | Block TMPRSS2 may reduce viral replication | Treatment of pancreatitis | |
| | Imatinib | TMPRSS2 inhibitor | PO | Theoretical hypothesis<br>NCT04422678 (EG)<br>NCT04356495 (FR) | Block virtual entry. | Treatment of chronic myelogenous leukemia (CML), gastrointestinal stromal tumors (GISTs) and a number of other malignancies | [100] |
| | alpha-1antitrypsin | TMPRSS2 inhibitor, alpha1-proteinase inhibitor | INH, 8 ml alpha1-proteinase inhibitor Glassia as nebulizer every 12 hours for 5 days | NCT04385836 (SA) | 1. Bind to elastase localized on the cell surface in which case;<br>2. Bind to neutrophil elastase released by inflammatory cells.<br>3. an Inhibitor of the SARS-CoV2–Priming Protease TMPRSS2 | Treatment of chronic obstructive pulmonary disease | [68] |
| | Bicalutamide | TMPRSS2 inhibitor, antiandrogen medication. | PO, 150 mg, 28 d | NCT04509999 (US)<br>NCT04374279 (US) | Block TMPRSS2 may reduce viral replication | Treatment of prostate cancer | |
| | Enzalutamide | TMPRSS2 inhibitor, hormone | PO, 40 mg, 28 d | NCT04456049 (CH)<br>NCT04475601 (SE) | Inhibit the expression of androgen regulated proteins, such as TMPRSS2, then stop the cleave of viral S glycoprotein | Treatment of prostate cancer | [101] |
| | Meplazumab | Antibody against CD147 | IV, 10 mg, 2 d | NCT04275245 (CN) | Block the infection of SARS-CoV-2. | Investigational drug | [72] |
| | Arbidol | Membrane fusion inhibitor and immunomodulator | PO, 200 mg, tid, < 10 d | Guidelines (version 8) for treatment of COVID-19<br>NCT04273763 (CN)<br>NCT04255017 (CN) | 1. Membrane fusion inhibition;<br>2. Stimulate humoral immune response, induces IFN-production | Antiviral treatment for influenza infection | [102] |
| | Suramin sodium | cGAS antogonist | IV, | ChiCTR2000030029<br>Artificial intelligence search | An entry inhibitor for various of viruses including DNA virus and RNA virus. | Treatment of trypanosomiasis | [103] |
| | Chlorpromazine | Cleavage of viral spike protein | PO, 75-300 mg | NCT04366739 (FR)<br>NCT04354805 (EG) | 1. Dopamine antagonist;<br>2. Anti-serotonergic;<br>3. Antihistaminergic properties;<br>4. Inhibit assembly and disassembly of Clathrin lattices on cell surfaces and endosomes by CPZ-HCl | Antipsychotic medication | |

© 2020. Peng Y et al. Published online at http://www.aginganddisease.org/EN/10.14336/AD.2020.1124

| | | | | | | | |
|---|---|---|---|---|---|---|---|
| | | | | | and prevent the entry of virus into host cells. | | |
| | Chloroquine | Receptor binding and membrane fusion inhibitor | PO, 500 mg bid, 7 d | Guidelines (version 8) for treatment of COVID-19<br>NCT04323527 (BR)<br>NCT04341727 (US) | 1. Increases late endosomal and lysosomal pH,block virus genetic material release into the cell;<br>2. Inhibit viral RNA-dependent RNA polymerase;<br>3. Iinhibit receptor binding and membrane fusion. | Prevent and treat malaria | [49] |
| | Hydroxychloroquine | Receptor binding and membrane fusion inhibitor | PO, 400 mg bid loading dose, 200 mg bid maintenance doses for 4d | NCT04308668 (US)<br>NCT04315948 (FR)<br>NCT04338698 (BR) | 1. Increases late endosomal and lysosomal pH,block virus genetic material release into the cell;<br>2. Inhibit viral RNA-dependent RNA polymerase;<br>3. Iinhibit receptor binding and membrane fusion. | Prevent and treat malaria | [104] |
| | | | INH, 20 or 50 mg/mL, 8 d | NCT04461353 (US)<br>NCT04477083 (EG) | | | |
| | Mefloquine | Receptor binding and membrane fusion inhibitor | PO, 250 mg, tid, Day 1<br>250 mg, bid, Day 2<br>250 mg, qd, Day 3-7 | NCT04347031 (RU) | 1. Increases late endosomal and lysosomal pH,block virus genetic material release into the cell;<br>2. Inhibit viral RNA-dependent RNA polymerase;<br>3. Iinhibit receptor binding and membrane fusion. | Prevent and treat malaria | |
| | Remdesivir | Adenosine analog, RdRP inhibitor | IV, 200 mg loading dose, 100 mg/d maintenance doses for 9d | NCT04252664 (CN)<br>NCT04401579 (US)<br>NCT04321616 (NO) | Adenosine nucleotide analog, inhibit Viral RNA-dependent RNA polymerase, control the virus replication. | Antiviral drug in the clinical trial stage | [105] |
| | Darunavir | Adenosine analog, RdRP inhibitor | PO | NCT04252274 (CN) | Adenosine nucleotide analog, inhibit Viral RNA-dependent RNA polymerase, control the virus replication. | Antiviral drug in the clinical trial stage | [106] |
| | Galidesivir | Adenosine analog, RdRP inhibitor | PO, | Artificial intelligence search<br>NCT03891420 (BR) | Adenosine nucleotide analog, inhibit Viral RNA-dependent RNA polymerase, control the virus replication. | Antiviral drug in the clinical trial stage | [107] |
| | Favipiravir | Adenosine analog, RdRP inhibitor | PO | NCT04310228 (CN)<br>NCT04336904 (IT)<br>NCT04346628 (US) | Adenosine nucleotide analog, inhibit Viral RNA-dependent RNA polymerase, control the virus replication. | Antiviral drug in the clinical trial stage | [106] |
| | Atazanavir | Adenosine analog, RdRP inhibitor | PO, 600 mg, bid in Day 1, 300 mg bid for 9 days. | NCT04468087 (BR) | Adenosine nucleotide analog, inhibit Viral RNA-dependent RNA polymerase, control the virus replication. | Antiviral drug in the clinical trial stage | |
| | Daclatasvir | Adenosine analog, RdRP inhibitor | PO, Initial dose of 120 mg, 60 mg, qd, 10d | NCT04468087 (BR)<br>NCT04460443 (EG) | Adenosine nucleotide analog, inhibit Viral RNA-dependent RNA polymerase, control the virus replication. | Antiviral drug in the clinical trial stage | |
| | Oseltamivir | Adenosine analog, RdRP inhibitor | PO, 75 mg, bid, 5d | NCT04338698 (PK)<br>NCT04255017 (CN)<br>NCT04338698 (BR) | Adenosine nucleotide analog, inhibit Viral RNA-dependent RNA polymerase, control the virus replication. | Antiviral drug in the clinical trial stage | |
| | Ribavirin | Guanosine analog, Nucleoside inhibitor | IV, 500 mg, bid or tid, in combination with IFN-α or lopinavir/ritonavir, < 10 d | Guidelines (version 8) for treatment of COVID-19 | Guanosine (ribonucleic) analog, used to stop viral RNA synthesis and viral mRNA capping. | Treatment of hepatitis C and viral hemorrhagic fevers | [49] |

© 2020. Peng Y et al. Published online at http://www.aginganddisease.org/EN/10.14336/AD.2020.1124

| | | | | | | | |
|---|---|---|---|---|---|---|---|
| | Triazavirin | Guanosine analog, Viral RNA-dependent RNA polymerase inhibitor/ CYP3A protein inhitor | PO, 250 mg, tid, 7 d | ChiCTR2000030001 | Guanine nucleotide analog, inhibit Viral RNA-dependent RNA polymerase, control the virus replication. | Treatment of influenza and other virus infections | [108] |
| | Clevudine | Nucleoside analog | PO, 120 mg, qd, 14 d | NCT04347915 (KR) | Pyrimidine analogue, triphosphate metabolite competes with thymidine for incorporation into viral DNA, thereby causing DNA chain termination and inhibiting the function of HBV DNA polymerase (reverse transcriptase). | Antiviral drug | |
| | Clofazimine | Nucleoside analog | PO, 100 mg, bid, 3 d | NCT04465695 (CN) | 1. Anti-inflammatory effect;<br>2. Immunosuppressive effects | Treatment of leprosy | |
| | Dipyridamidole | Nucleoside transport inhibitor and a PDE3 inhibitormedication | PO, 150 mg, tid, 7 d | ChiCTR2000030055 | 1. Reduce viral replication;<br>2. Suppress hypercoagulability;<br>3. Enhancing immune recovery. | Used to dilate blood vessels | [109] |
| | Azvudine | Reverse transcriptase inhibitor | PO, 5 mg, qd | ChiCTR2000029853 | Reverse transcriptase inhibitor. | Investigational drug for HCV | [110] |
| | Decitabine | Nucleic Acid Synthesis Inhibitor | IV, 10 mg/m^2, 5 d | Artificial intelligence searchNCT04482621 (US) | RdRp inhibitor | Treatment of myelodysplastic syndromes, a class of conditions where certain blood cells are dysfunctional, and for acute myeloid leukemia | [111] |
| | Selinexor | XPO1 inhibitor | PO | In vitro model NCT04534725 (AU) NCT04349098 (US) | Selective inhibitor of nuclear export (SINE) compound that blocks cellular protein XPO1 and interfere with key host protein interactions with SARS-CoV-2. | Treatment of relapsed/refractory multiple myeloma | |
| | ABX464 | Moleclure binding to the cap binding complex (CBC) of the viral mRNA | PO, 50 mg qd for 28 d | NCT04393038 (BE, FR, DE, BR, IT, MZ, ES, GB) | 1. Antiviral effect to inhibit the SARS-CoV-2 viral replication;<br>2. Anti-inflammatory effect to prevent and treat the acute respiratory distress syndrome (ARDS);<br>3. Tissue repair properties preventing longer-term pulmonary dysfunction. | Anti-HIV thearpy | [112] |
| | PF-07304814 | SARS-CoV-2 3CL protease inhibitor | IV | NCT04535167 (US) | SARS-CoV-2 3CL protease inhibitor to block virtral replication | Investigational drug | |
| | Brequinar | Dihydroorotate dehydrogenase (DHODH) inhibitor | IV, 100 mg, 5 d | NCT04425252 (US) | Synthetic quinolinecarboxylic acid analogue, suppress host nucleotide synthesis | Treatment of tumor | |
| | PTC299 | DHODH inhibitor | PO, 200 mg, bid for 7 d, followed 50 mg, qd for 7 d | NCT04439071 (US) | Reduce the high viral replication and also selectively attenuate the immune response | Investigational drug for tumor | |
| | Leflunomide | DHODH inhibitor | PO, 20 mg, qd | ChiCTR2000030058 | Inhibiting dihydroorotate dehydrogenase. | Treatment of active moderate-to-severe | [113] |

| | | | | | | | |
|---|---|---|---|---|---|---|---|
| | | | | NCT04361214 (US) | | rheumatoid arthritis and psoriatic arthritis | |
| | Fenofibrate | Fibrate class | PO, 145 mg/d, 10 d | NCT04517396 (US) | Inhibite SARS-CoV-2 viral replication | Medication of the fibrate class used to treat abnormal blood lipid levels. | |
| | Atovaquone | Atovaquone analog | PO, 1500 mg, bid | NCT04456153 (US) | Inhibit the replication of SARS-CoV-2 in vitro | Treatment or prevention of pneumocystis pneumonia, toxoplasmosis, malaria and babesia | |
| | Ivermectin | - | PO, 6 mg, tid | NCT04510233 (EG) NCT04530474 (US) NCT04529525 (AR) | Inhibitor of the COVID-19 causative virus (SARS-CoV-2) in vitro. | Treatment of certain parasitic roundworm infections. | |
| | | | 1 mL per nostril, bid, ISIN | NCT04510233 (EG) | | | |
| | | | IV, 0.2 mg/kg | NCT04431466 (BR) | | | |
| | Niclosamide | - | PO, 2 g/d, 14 d | NCT04399356 (US) NCT04524052 (JP) | Inhibit SARS-CoV replication | Treatment of tapeworm infestations | [114] |
| | Ebselen | Glutathione peroxidase mimetic | PO, 400 or 800 mg, bid, 14 d | NCT04483973 (US) NCT04484025 (US) | Against COVID-19 by irreversible inhibition of the main protease via a covalent bond formation with the thiol group of the active center's cysteine (Cys-145) | Investigational drug for reperfusion injury and stroke | |
| | Opaganib | Sphingosine Kinase-2 (SK2) Inhibitor | PO, 500 mg, bid | NCT04502069 (IL) NCT04467840 (MX, RU) | Anti-viral and anti-inflammatory activity via selective SphK2 inhibition | Investigational drug for tumor | |
| | MPT0B640 | Heat Shock Protein Inhibitor | PO, 15, 30, 60, 80 mg | NCT04526717 (KR) | inhibit hsp90, which emerged as a major component enables viruses to hijack infected cells through the process of autophagy | Investigational drug for tumor | |
| | DAS181 | Recombinant sialidase | INH, 9 mg/d, 10 d | NCT04324489 (CN) NCT04338698 (BR) | - | Treatment of parainfluenza and influenza in clinical trials | |
| | EIDD-2801 | Ribonucleoside analog | PO, 200 or 300 mg | NCT04405739 (US) | Increase transition mutation frequency in viral | Investigational drug for broad-spectrum antiviral | [115] |
| | Bismuth potassium citrate | NTPase and RNA helicase Inhibitor | PO, 0.6 g, bid | ChiCTR2000030398 | Inhibit both the NTPase and RNA helicase activities of SARS-CoV-2 nsp13. | Used in combination with antibiotics and a proton pump inhibitor for the treatment of Helicobacter pylori infections | [93] |
| | Azithromycin | Tetrcycline | PO, 500 mg bid | NCT04381962 (GB) NCT04332107 (US) NCT04365582 (FR) | 1. The coronaviruses is known to bind to metalloproteases (MMPs) of the host, in particular to ensure viral survival. Tetracyclines are known to chelate zinc from MMPs to limite virtal replicate in the host. 2. Inhibit the replication of positive-polarity single-stranded RNA viruses. 3. Be modulators of innate immunity (anti-inflammatory activity), decrease the expression of NFKB, the release of inflammatory cytokines | Antibiotic drug | |

© 2020. Peng Y et al. Published online at http://www.aginganddisease.org/EN/10.14336/AD.2020.1124

| | | | | | | | |
|---|---|---|---|---|---|---|---|
| | | | | | such as TNF-α, IL-1β and IL-6, inhibit granulomas inflammatory and free radical release. | | |
| | Carrimycin | Tetrcycline | PO, 400 mg, qd, 7 d | NCT04286503 (CN) | - | Treatment of Mycobacterium tuberculosis infection | [116] |
| | Doxycycline | Tetrcycline | PO, 200 mg, 14 d | NCT04371952 (FR) NCT04528927 (TN) NCT04433078 (US) | 1. The coronaviruses is known to bind to metalloproteases (MMPs) of the host, in particular to ensure viral survival. Tetracyclines are known to chelate zinc from MMPs to limite virtal replicate in the host.<br>2. Inhibit the replication of positive-polarity single-stranded RNA viruses.<br>3. Be modulators of innate immunity (anti-inflammatory activity), decrease the expression of NFKB, the release of inflammatory cytokines such as TNF-α, IL-1β and IL-6, inhibit granulomas inflammatory and free radical release. | Antibiotic drug | |
| | XAV-19 | Anti-SARS-CoV-2 antibody | IV, 0.5 or 2 mg/kg, Day 1 and 5 | NCT04453384 (FR) | Polyclonal humanized anti-SARS-CoV2 antibodies (XAV-19) | Investigational drug for COVID-19 | |
| | AZD7442 | Anti-SARS-CoV-2 antibody | IV and IM | NCT04507256 (GB) | A combination of two mAbs derived from convalescent patients with SARS-CoV-2 infection. | Investigational drug for COVID-19 | |
| | JS016 | Anti-SARS-CoV-2 antibody | IV | NCT04441918 (CN) | - | Investigational drug for COVID-19 | |
| | SCTA01 | Anti-SARS-CoV-2 antibody | IV | NCT04483375 (CN) | Neutralize virus | Investigational drug for COVID-19 | |
| | Anti-SARS-CoV-2 equine immunoglobulin fragments (INOSARS) | Anti-SARS-CoV-2 Equine Antibody Fragments | IV, 150 mL，4.0 mL/min over 40 min. | NCT04514302 (MX) | Recombinant SARS-CoV-2 spike protein receptor-binding domain (RBD) | Investigational drug for COVID-19 | [117] |
| | CT-P59 | Anti-SARS-CoV-2 spike RBD antibody | IV | NCT04525079 (KR) | A monoclonal antibody targeted against SARS-CoV-2 spike RBD as a treatment for SARS CoV 2 infection. | Investigational drug for COVID-19 | |
| | LY3819253 | SARS-CoV-2 neutralizing antibody | IV | NCT04497987 (US) NCT04518410 (US) | Neutralizing IgG1 monoclonal antibody (mAb) directed against the spike protein of SARS-CoV-2 | Investigational drug for COVID-19 | |
| | BGB DXP593 | SARS-CoV-2 neutralizing antibody | IV, 10-30 mg/kg | NCT04532294 (AU) | Viral neutralisation. | Investigational drug for COVID-19 | |
| | Hyperimmune plasma or Convalescent plasma treatment | - | IV | NCT04261426 (CN) NCT04323800 (US) NCT04483960 (AU) | - | - | [118] |
| | INM005 | Equine hyperimmune serum | IV, 4 mg/kg | NCT04494984 (AR) | - | - | |
| | hyper immunoglobulins containing | Immunoglobulin | IV | NCT04383548 (EG) | 1. Activation of complement;<br>2. Activation of effector | - | |

| | | | | | | | |
|---|---|---|---|---|---|---|---|
| | anti-Corona VS2 immunoglobuli n | | | | cells;<br>3. Natural antibodies;<br>4. puried from COVID19 convalescent plasma | | |
| | hyper immunoglobuli ns containing anti-Corona VS3 immunoglobuli n | Immunoglo bulin | IV | NCT04521322 (AR) | 1. Activation of complement;<br>2. Activation of effector cells;<br>3. Natural antibodies;<br>4. puried from COVID19 convalescent plasma | - | |
| | Octagam | Immunoglo bulin | IV, 0.5 g/kg | NCT04411667 (US) | - | - | |
| | SAB-185 | Immunoglo bulin | IV, 10, 25 and 50 mg/kg | NCT04469179 (US)<br>NCT04468958 (US) | An anti-SARS-CoV-2 Human Immunoglobulin Intravenous (transchromosomic [Tc] bovine-derived) | - | |
| | SAR443122 | Immunoglo bulin | PO, 14 d | NCT04469621 (AR, BR, CL, MX, RU) | Improve hyperinflammatory state | Investigational drug for Systemic Inflammatory disease | |
| | Interferon-α | Immunomo dulator | IFN-α: 5 million U or equivalent dose, bid, < 10 d, INH | Guidelines (version 8) for treatment of COVID-19<br>NCT04534725 (AU) | Involved in innate immunity against viral infection. | Antiviral treatment | [49] |
| | | | IFN-α1β: 2-3 drops for each nostril, 4 times per day, 28 d, ISIN | NCT04320238 (CN) | 1. Activation of complement;<br>2. Activation of effector cells;<br>3. Natural antibodies. | Treatment of rheumatoid arthritis, multiple sclerosis, psoriasis, many forms of cancer including non-Hodgkin's lymphoma, colorectal cancer, head and neck cancer and breast cancer | [119] |
| | | | Cerrokin: INH, 10 μg, bid, 10 d | ChiCTR200003048 0 | Recombinant human IFN-alpha 1beta.<br>1. Activation of complement;<br>2. Activation of effector cells;<br>3. Natural antibodies. | Antiviral or antineoplastic drug | |
| | | | IFN-α2β: INH, 10 μg, bid, 10 d | NCT04293887 (CN)<br>NCT04379518 (USA) | 1. Activation of complement;<br>2. Activation of effector cells;<br>3. Natural antibodies. | Antiviral or antineoplastic drug | [120] |
| | Inovaferon-β | Immunomo dulator | IFN-β: 20 μg, bid, INH, 7-14 d | ChiCTR200002949 6 | Involved in innate immunity against viral infection. | Antiviral treatment | [121] |
| | | | IFN-β1α: 44 mcg, three times per week, 14 d, SC | NCT04449380 (IT) | | | |
| | | | SNG001 IFN-β1α: INH | NCT04385095 | been well tolerated during virus infections enhanced antiviral activity in the lungs (measured in sputum and blood samples) provided significant lung function benefit over placebo in asthma in two Phase II trials. | | |
| | | | IFN-β1β: 9.6 million U x2/d for 48 hours, then 9.6 million U x1/d for 8 to 16 d), INH | NCT04469491 (FR) | 1. Balances the expression of pro- and anti-inflammatory agents;<br>2. Leads to a reduction of neuron inflammation. | Treatment of the relapsing-remitting and secondary-progressive forms of multiple sclerosis (MS) | |
| | Peg IFN-λ1α | Immunomo dulator | IM, 0.5 mL, 180 mcg | NCT04354259 (CA)<br>NCT04344600 (US) | Involved in innate immunity against viral infection. | Antiviral treatment | |

© 2020. Peng Y et al. Published online at http://www.aginganddisease.org/EN/10.14336/AD.2020.1124

# SUPPLEMENTARY DATA

| | | | | | | | |
|---|---|---|---|---|---|---|---|
| | | | | NCT04388709 (US) | | | |
| | IL-7 | Immunomodulator | IM, 10 µg/kg | NCT04407689 (FR) NCT04379076 (GB) NCT04442178 (US) | Restore lymphocyte count in several viral infections | Treatment of metastatic breast cancer. | [122] |
| | NT-I7 (efineptakin alfa) | Immunomodulator | IM | NCT04501796 (US) NCT04498325 (US) | Long Acting IL-7: Restore lymphocyte count in several viral infections | Treatment of metastatic breast cancer. | |
| | Thymosin | Immunomodulator | SC or IV, 1.6 mg, qd, 5 d | NCT04320238 (CN) NCT04268537 (CN) | Regulate cellular immunity in sepsis patients. | Treatment of septic shock, acute respiratory distress syndrome, peritonitis, acute cytomegalovirus infection and infectious disease | [123] |
| | Thymalfasin | Immunomodulator | SC, 1.6 mg, twice per week | NCT04428008 (US) NCT04487444 (US) | A biological response modifier which activates various cells of the immune system, | Treatment of Hepatitis B and C | |
| | PUL-042 | TLR 2/6/9 agonist | INH, 50 µg on Day 1, Day 3 and Day 6 | NCT04313023 (US) NCT04312997 (US) | Amplify the innate immune defenses of the lung epithelial mucosa. | Preclinical Investigational drug | [95] |
| | Polyinosinic:polycytidylic acid | Immunostimulant | IM, 2 mg, once every 2 days | ChiCTR2000029776 | Structurally similar to double-stranded RNA, which is present in some viruses and is a "natural" stimulant of TLR3. | Used to simulate viral infections in scientific research on the immune system | [96] |
| | Apolactoferrin | Immunomodulator | PO, 600 - 1500 mg, bid, > 8 w | NCT04412395 (EG) NCT04475120 (IT) | 1. Block the viral receptors on the host cells to prevent viral entry and replication; 2. Protect against the cytokine-storm and thrombotic complications | Food supplement | |
| | Lactoferrin | Immunomodulator | PO, 200 or 300 mg, bid | NCT04421534 (EG) | Lactotransferrin: antimicrobial activity and antiviral activity | - | |
| | Desferal | Immunomodulator | IV | NCT04333550 (IR) | Iron Chelator: Antiviral and immunomodulatory effects in vitro and in vivo | Ttreatment of acute iron intoxication and of chronic iron overload due to transfusion-dependent anemias | |
| | Nitazoxanide | Immunomodulator | PO, 1g, bid, 7 d | NCT04359680 (US) NCT04463264 (AR) NCT04498936 (EG) | Amplify cytoplasmic RNA sensing, potently augment type I interferon and autophagy-mediated anti-viral responses, have immunomodulatory properties eg inhibits macrophage IL-6 production, and interferes with SARS-CoV-2 glycosylation | Antiprotozoal drug | |
| | KB109 | Immunomodulator, Glycan | PO, | NCT04486482 (US) NCT04414124 (US) | Microbiome Metabolic Therapy | Investigational drug for COVID-19 | |
| | NK cell | NK cell therapy | IV, 0.1-2x10^7 cells/kg, twice a week | NCT04280224 (CN) NCT04344548 (CO) | Natural killer (NK) cells are innate lymphocytes that may serve as useful effectors against danger infection. | Anti-cancer therapy | |
| | FT516 | NK cell therapy | IV, 9 × 10^7 cells, Day 1 and Day 4 | NCT04363346 (US) | Natural killer (NK) cells are innate lymphocytes that may serve as useful effectors against danger infection. | - | |
| | CYNK-001 | NK cell therapy | IV | NCT04365101 (US) | An allogeneic off the shelf cell therapy enriched for | - | |

© 2020. Peng Y et al. Published online at http://www.aginganddisease.org/EN/10.14336/AD.2020.1124

| | | | | | | | |
|---|---|---|---|---|---|---|---|
| | | | | | CD56+/CD3- NK cells expanded from human placental CD34+ cells. | | |
| | NKG2D-ACE2 CAR-NK cell | NK cell therapy | IV, 1x10^8 cells/kg, once a week | NCT04324996 (CN) | Cord blood derived NKG2D-ACE2 CAR-NK cell, carried IL15 superagonist- and GM-CSF neutralizing scFv-secreting | Anti-cancer therapy | |
| | Stem Cell Educator-Treated Mononuclear Cells Apheresis | Stem cell therapy | IV | NCT04299152 (CN) | Stem Cell Educator (SCE) therapy functions via CB-SC induction of immune tolerance in autoimmune T cells and restore immune balance and homeostasis. | Treatment of inflammatory | |
| | BCG Vaccine | Immunostimulant | ID, 2x10^5 to 8x10^5 U | NCT04327206 (AU) NCT04348370 (US) NCT04387409 (DE) | Trained immunity, BCG inducescross-protection. | Treatment of tuberculosis, mycobacteria, and non–muscle-invasive bladder cancer | [97] |
| | NasoVAX | Immunostimulant | IM | NCT04442230 (US) | Efficient Adenovirus vector to express viral Spike protein. | Investigational vaccine for COVID-19 | |
| | BACMUNE | Immunostimulant | SL, 2 spray puff every 12 hours, 45 d | NCT04452643 (MX) | A bacterial preparation that contains a mixture of Gram + and Gram - inactivated bacteria | Investigational drug for infectious disease | |
| | IMM-101 | Immunostimulant | SC, 1.0 mg Day1, 0.5 mg Day 2 and Day 3 | NCT04442048 (CA) | made from the heat-treated bacterium Mycobacterium obuense. | Investigational drug for tumor | |
| | Naproxen | Inhibitor of both cyclo oxygenase (COX-2) and of Influenza A virus nucleoprotein (NP) | PO, 250 mg, bid | NCT04325633 (FR) | 1. Binding competed with NP association with viral RNA and impeded the NP self-association process which strongly reduced viral transcription/replication; 2. Anti-inflammation. | Nonsteroidal anti-inflammatory drugs | [124] |
| | Famotidine | Histamine-2 blocker | PO, 20 mg, bid, 28 d | NCT04389567 (US) | Histamine-2 blocker, Unknown mechanism | Treatment and prevention of ulcers in the stomach and intestines | |
| | Linagliptin | DPP4 inhibitor | PO, 5 mg, 14 d | NCT04341935 (US) | - | Treatment of diabetes mellitus type 2 | |
| | Niclosamide | - | PO, 2 g/d, 14 d | NCT04399356 (US) NCT04524052 (JP) | Inhibit SARS-CoV replication | Treatment of tapeworm infestations | [114] |
| | Povidone-Iodine | - | ISIN, 0.5% or 2%, 4 times each nare, 5 d | NCT04371965 (FR) | Highly virucidal against | Antiseptic drug | |
| | Iodine Complex | - | PO, 200 mg, tid | NCT04473261 (PK) | Clear viral load | - | |
| **Corticosteroids** | Dexamethasone | Corticosteroids | IV, 20 mg qd for 5 days, then 10 mg qd for 5 days | NCT04325061 (ES) NCT04327401 (BR) NCT04344730 (FR) | Inhibit excessive inflammation. | A type of corticosteroid medication | |
| | Ciclesonide | Corticosteroids | INH, 320 μg, bid, 14 d | NCT04330586 (KR) | Inhibit excessive inflammation. | Treatment of asthma and hay fever | [125] |
| | Methylprednisolone | Corticosteroids | IV, 40-80 mg, qd. 7 d | NCT03852537 (US) NCT04244591 (CN) NCT04323592 (IT) | Inhibit excessive inflammation. | A type of corticosteroid medication | [126] |
| | Budesonide | Corticosteroids | INH, 1mg/2mL diluted in 250mL, 30 d | NCT04361474 (FR) NCT04355637 (ES) | Inhibit excessive inflammation. | A type of corticosteroid medication | |
| | Hydrocortisone | Corticosteroids | IV, 200 mg/ 24 h, continuous infusion; 50 mg/ 6h, bolus injections, 7 d | NCT04348305 (DK) | Inhibit excessive inflammation. | A type of corticosteroid medication | |

© 2020. Peng Y et al. Published online at http://www.aginganddisease.org/EN/10.14336/AD.2020.1124

# SUPPLEMENTARY DATA

| | | | | | | | |
|---|---|---|---|---|---|---|---|
| **Treatment of Cytokine release syndrome** | Immunoglobulin | Immunomodulator | IV, 0.5g / kg / d, 5 d | NCT04261426 (CN)<br>NCT04381858 (MX)<br>NCT04381936 (GB) | 1. Activation of complement;<br>2. Activation of effector cells;<br>3. Natural antibodies. | Treatment of rheumatoid arthritis, multiple sclerosis, psoriasis, many forms of cancer including non-Hodgkin's lymphoma, colorectal cancer, head and neck cancer and breast cancer | [118] |
| | Infliximab | Anti-TNF antibody | IV, 5 mg/Kg | NCT04425538 (US) | Involve in the inflammatory process, whereas the receptor fusion protein apparently lacks this capability. | Treatment of a number of autoimmune diseases | |
| | Emapalumab | Anti-IFNγ antibody | IV, 6 mg/kg on Day 1, Day 4, Day 7, Day 10 and Day 13 | NCT04324021 (IT) | Bind and neutralize IFN-γ, preventing it from inducing pathological effects. | Treatment of hemophagocytic lymphohistiocytosis | [127] |
| | Tocilizumab | Anti-IL-6R antibody | IV, 8 mg/kg, 6 times in 4 weeks | Guidelines (version 8) for treatment of COVID-19<br>NCT04363736 (US)<br>NCT04315480 (IT)<br>NCT04331808 (FR) | Hinder IL-6 from exerting its pro-inflammatory effects. | Treatment of rheumatoid arthritis (RA) and systemic juvenile idiopathic arthritis | [49] |
| | CMAB806 | Anti-IL-6R antibody | IV | ChiCTR2000030196 | Hinder IL-6 from exerting its pro-inflammatory effects. | Treatment of rheumatoid arthritis (RA) and systemic juvenile idiopathic arthritis | [128] |
| | Sarilumab | Anti-IL-6R antibody | SC, 200 mg | NCT04315298 (US)<br>NCT04324073 (FR)<br>NCT04386239 (IT) | hinder IL-6 from exerting its pro-inflammatory effects. | Treatment of rheumatoid arthritis (RA) and systemic juvenile idiopathic arthritis | [67] |
| | Siltuximab | Anti-IL-6R antibody | IV, 11 mg/kg, 28 d | NCT04330638 (BE) | hinder IL-6 from exerting its pro-inflammatory effects. | Treatment of rheumatoid arthritis (RA) and systemic juvenile idiopathic arthritis | [129] |
| | Clazakizumab | Anti-IL-6 antibody | IV, 12.5 or 25mg in 50 mL infusion over 30 minutes | NCT04494724 (US)<br>NCT04343989 (US) | Anti-IL-6, aglycosylated, humanized rabbit monoclonal antibody | Treatment of musculoskeletal aspects of psoriatic arthritis | |
| | Olokizumab | Anti-IL-6 antibody | SC, 64 mg | NCT04452474 (US)<br>NCT04380519 (RU) | Hinder IL-6 from exerting its pro-inflammatory effects. | Treatment of rheumatoid arthritis (RA) and systemic juvenile idiopathic arthritis | |
| | Sirukumab | Anti-IL-6 antibody | IV, 5 mg/Kg | NCT04380961 (BE) | against the proinflammatory cytokine IL- 6 (IL-6). | Treatment of rheumatoid arthritis | |
| | Infliximab | Anti-TNF antibody | IV, 5 mg/Kg | NCT04425538 (US) | Involve in the inflammatory process, whereas the receptor fusion protein apparently lacks this capability. | Treatment of a number of autoimmune diseases | |
| | Emapalumab | Anti-IFNγ antibody | IV, 6 mg/kg on Day 1, Day 4, Day 7, Day 10 and Day 13 | NCT04324021 (IT) | Bind and neutralize IFN-γ, preventing it from inducing pathological effects. | Treatment of hemophagocytic lymphohistiocytosis | [127] |
| | BMS-986253 | Immunomodulator | IV, 2400 mg | NCT04347226 (US) | Anti-IL-8 | Treatment of tumor | |
| | Canakinumab | Immunomodulator | IV, 450-750 mg | NCT04362813 (US)<br>NCT04510493 (CH)<br>NCT04348448 (IT) | Anti-IL-1 | Treatment of systemic juvenile idiopathic arthritis and active Still's disease, including adult-onset Still's disease | |
| | Anakinra | IL- 1R antagonist protein | IV, 100 mg, qid, 15 d | NCT04324021 (IT)<br>NCT04339712 | Recombinant and slightly modified version of the human IL- 1 receptor | Treatment of rheumatoid arthritis | [130] |

© 2020. Peng Y et al. Published online at http://www.aginganddisease.org/EN/10.14336/AD.2020.1124

| | | | | | | | |
|---|---|---|---|---|---|---|---|
| | | | | (GR) NCT04364009 (FR) | antagonist protein. Bind IL1R, preventing it from inducing pathological effects. | | |
| | RPH-104 | Heterodimeric fusion protein containing IL-1R1 and IL-1RAcP linked to immunoglobulin heavy chains | SC, 80 mg | NCT04380519 (RU) | Inhibit human IL-1 beta/IL-1F2 signaling pathway, | Treatment of rheumatoid arthritis (RA) and systemic juvenile idiopathic arthritis | |
| | UTTR1147A (IL-22Fc) | IL - 22Fc protein | IV | NCT04386616 (US) | human IL - 22Fc (immunoglobulin G (IgG)4) fusion protein plays role of tissue regeneration, acting directly on epithelial cells | - | |
| | MSTT1041A (astegolimab) | Anti-ST2 antibody | IV | NCT04386616 (US) | Inhibit binding of IL-33 (IL-33) to the ST2 receptor. | Investigational drug for moderate to severe atopic dermatitis | |
| | Lenzilumab | Anti-GM-CSF antibody | IV, 600 mg | NCT04351152 (US) NCT04534725 (AU) | anti-human GM-CSF monoclonal antibody, alleviate the immune-mediated cytokine release syndrome | Investigational drug for treatment of chronic myelomonocytic leukemia (CMML) and juvenile myelomonocytic leukemia (JMML) | |
| | Gimsilumab | Anti-GM-CSF antibody | IV, 10 mg/kg | NCT04351243 (US) | Recombinant anti-granulocyte colony-stimulating factor (G-CSF) receptor monoclonal antibody | Investigational drug for multiple inflammatory diseases and cancer | |
| | Otilimab | Anti-GM-CSF antibody | IV | NCT04376684 (US) | Recombinant anti-granulocyte colony-stimulating factor (G-CSF) receptor monoclonal antibody | Investigational drug for multiple inflammatory diseases and cancer | |
| | mavrilimumab | Anti-GM-CSF-Rα antibody | IV, 6, 10 mg/kg | NCT04447469 (US) NCT04397497 (IT) | Anti-granulocyte-macrophage colony-stimulating factor receptor alpha (GM-CSF-Rα) monoclonal antibody | Treatment of rheumatoid arthritis | |
| | CSL324 | Anti-G-CSF antibody | IV | NCT04519424 (AU) | Recombinant anti-granulocyte colony-stimulating factor (G-CSF) receptor monoclonal antibody | Investigational drug for rheumatoid arthritis, vasculilitis, cystic fibrosis and inflammatory bowel disease | |
| | TJ003234 | Anti-G-CSF antibody | IV, 3 or 6 mg/kg | NCT04341116 (US) | Recombinant anti-granulocyte colony-stimulating factor (G-CSF) receptor monoclonal antibody | Investigational drug for rheumatoid arthritis | |
| | Nangibotide | TREM-1 inhibitor | IV, 1.0 mg/kg/h | NCT04429334 (FR) | Inhibit TREM-1to prevent the inflammatory activation. | Investigational drug for septic shock | |
| | IC14 | CD14 antibody | IV, initial dose of 4 mg/kg, 2 mg/kg for 3 days | NCT04391309 (US) NCT04346277 (IT) | Blockade of PAMP and DAMP interactions with CD14, thus attenuating the inflammatory cascade that leads to increased endothelial and epithelial permeability and injury resulting in alveolar injury and fluid accumulation characteristic of ARDS. | Investigational drug for amyotrophic lateral sclerosis | [131] |
| | EB05 | TLR4 antibody | IV | NCT04401475 | Inhibit TLR4, which is a key component of the innate immune system | | |

© 2020. Peng Y et al. Published online at http://www.aginganddisease.org/EN/10.14336/AD.2020.1124

| | | | | | | | |
|---|---|---|---|---|---|---|---|
| | | | | | which functions to detect molecules generated by pathogens, acting upstream of cytokine storm and IL-6-mediated acute lung injury. | | |
| | M5049 | TLR inhibitor | PO, 100 or 200 mg, 14 d | NCT04448756 (US, BR) | A potentially first-in-class small molecule that blocks the activation of Toll-like receptor (TLR)7 and TLR8, two innate immune sensors that detect single-stranded (ss) RNA from viruses such as SARS-CoV-2, the virus responsible for COVID-19. | Investigational drug for lupus pathogenesis | |
| | Baricitinib | JAK inhibitor | PO, 2 mg/d, 4 mg/d | Artificial intelligence search NCT04320277 (IT) NCT04340232 (US) NCT04362943 (ES) | 1. An inhibitor of janus kinase (JAK), blocking the subtypes JAK1 and JAK2; 2. dual antiviral and antiinflammatory activity in vitro. | Rheumatoid arthritis treatment | [132] |
| | Ruxolitinib | JAK inhibitor | PO, 10 mg bid for 14 d, then 5 mg bid for 2 d, 5 mg qd for 1d | ChiCTR2000029580 NCT04331665 (CA) NCT04334044 (MX) NCT04348071 (US) | 1. An inhibitor of janus kinase (JAK), blocking the subtypes JAK1 and JAK2; 2. Antiinflammatory activity in vitro. | Treatment of intermediate or high-risk myelofibrosis | [133] |
| | Jakotinib | JAK inhibitor | PO, 50 mg, bid, 7 d | ChiCTR2000030170 | 1. An inhibitor of janus kinase (JAK), blocking the subtypes JAK1 and JAK2; 2. Antiinflammatory activity in vitro. | Investigational drug | [134] |
| | Pacritinib | JAK inhibitor | PO, 400 mg/d | NCT04404361 (US) | 1. An inhibitor of janus kinase (JAK), blocking the subtypes JAK1 and JAK2; 2. dual antiviral and antiinflammatory activity in vitro. | Treatment of myelofibrosis | |
| | TD-0903 | JAK inhibtor | INH | NCT04402866 (GB) NCT04350736 (GB) | 1. An inhibitor of janus kinase (JAK), blocking the subtypes JAK1，JAK2，JAK3 and TYK2; 2. Block the release of cytokines and chemokines that may be associated with Acute Lung Injury and the initiation of a cytokine storm syndrome. | Investigational drug for acute lung injury | |
| | Tofacitinib | Jak-1/3 inhibitor | PO, 10 mg, bid, 14 d | NCT04412252 (US) NCT04415151 (US) | 1. An inhibitor of janus kinase (JAK), blocking the subtypes JAK1 and JAK2; 2. dual antiviral and antiinflammatory activity in vitro. | Treatment of rheumatoid arthritis, psoriatic arthritis, ankylosing spondylitis, and ulcerative colitis | |
| | ATI-450 | MAPKAPK 2 (MK2) inhibitor | PO, 50 mgqd for 14 d | NCT04481685 (US) | Regulate pro-inflammatory cytokines associated with CRS. | Investigational drug for COVID-19 and rheumatoid arthritis | |
| | CERC-002 | LIGHT inhibitor | SC, 16 - 1200 mg/kg | NCT04412057 (US) | LIGHT inhibitor, to ameliorate the cytokine storm | Investigational drug for COVID-19 | |
| | CD24Fc | CD24Fc protein | IV, 480 mg, in 60 min | NCT04317040 (US) | 1. CD24Fc comprises the nonpolymorphic regions of CD24 attached to the Fc region of human IgG1; 2. Ameliorate production of multiple inflammatory cytokines and reverse the loss of T lymphocytes. | Investigational drug for COVID-19 | |
| | Eculizumab | Immunomodulator | IV, 900 mg, once a week | NCT04288713 (US) NCT04346797 (FR) | Modulate the activity of the distal complement and prevent the formation of | Treatment of paroxysmal nocturnal hemoglobinuria | [135] |

© 2020. Peng Y et al. Published online at http://www.aginganddisease.org/EN/10.14336/AD.2020.1124

| | | | | | | | |
|---|---|---|---|---|---|---|---|
| | | | | NCT04355494 (US) | the membrane attack complex. | (PNH), atypical hemolytic uremic syndrome (aHUS), and neuromyelitis optica | |
| | Zilucoplan® | Complement C5 inhibitor | SC, 14 d | NCT04382755 (BE) | Improve the lung damage caused by the excessive activation of complement caused by the virus. | - | |
| | Avdoralimab | Anti-C5aR Antibody | IV | NCT04371367 (FR) | Block C5aR and reduce the inflammatory response in the lungs. | Immunomodulator in clinical trials | |
| | BDB-001 | Anti-C5a Antibody | IV | NCT04449588 (CN) | Improve the lung damage caused by the excessive activation of complement caused by the virus. | Immunomodulator in clinical trials | [106] |
| | Leronlimab | CCR5 antagonist | IV, 700 mg weekly | CytoDyn Inc. NCT04343651 (US) NCT04347239 (US) | 1. Enhance the immune response;<br>2. Mitigate the "cytokine storm" . | Treatment of HIV/AIDS | [136] |
| | Maraviroc | Chemokine receptor antagonist | PO, 300 mg, bid, 14 d | NCT04441385 (ES) NCT04475991 (MX) NCT04435522 (US) | CC Chemokine Receptor 5 (CCR5) antagonist, CCR5 antagonism prior to the 'second wave' of inflammatory mediator expression in SARS-CoV-2 may reverse lymphoid depletion and may alter cell trafficking of inflammatory cells, both increasing viral control capacity and dampening damage to lung tissue. | Investigational drug for HIV | |
| | AMY-101 | C3 Inhibitor | IV, 5 mg/Kg/d, 14 d | NCT04395456 (US) | Inhibit complement activation and contain systemic, complement-mediated inflammatory reactions by C3 interception. | Investigational drug for membranous glomerulonephritis and paroxysmal nocturnal haemoglobinuria | [137] |
| | PD-1 blocking antibody | PD-1 antibody | IV, 200 mg | NCT04268537 (ES) | Block PD-1 or PD-L1 can prevent T cell death, regulate cytokine production, reduce organ dysfunction and reduce death in sepsis. | Treatment of cancer | [138] 2 |
| | Nivolumab | PD-1 antibody | IV, 240 mg | NCT04413838 (FR) NCT04343144 (FR) NCT04356508 (CN) | Estore exhausted T lymphocytes immunity. | Treatment of tumor | |
| | Abatacept | Anti-CTLA-4 antibody | IV, 10 mg/kg on Day 1 | NCT04477642 (US) NCT04472494 (US) | A fusion protein composed of the Fc region of the immunoglobulin IgG1 fused to the extracellular domain of CTLA-4, inhibits T cell co-stimulation. | Treatment of autoimmune diseases like rheumatoid arthritis | |
| | Itolizumab | Anti-CD6 antibody | IV, 1.6 mg/kg | NCT04475588 (IN) | Bind to CD6, down regulates T cell activation, causes reduction in synthesis of pro-inflammatory cytokines and possibly plays an important role by reducing T cell infiltration at sites of inflammation. | Treatment of psoriasis. | |
| | AK119 | Immunomodulator | Anti-CD73 Antibody: IV | NCT04516564 (NZ) | 1. 5-nucleotidase inhibitors;<br>2. Antibody-dependent cell cytotoxicity;<br>3. T lymphocyte stimulants. | Treatment of tumor | |

© 2020. Peng Y et al. Published online at http://www.aginganddisease.org/EN/10.14336/AD.2020.1124

# SUPPLEMENTARY DATA

| | | | | | | | |
|---|---|---|---|---|---|---|---|
| | Fingolimod | Sphingosine 1-phosphate receptor modulator | PO, 0.5 mg, 3 d | NCT04280588 (CN) | Immune regulation effects. | Treatment of multiple sclerosis in clinical trial | [139] |
| | Acalabrutinib | BTK inhibitor | | NCT04497948 (US) NCT04380688 (US) NCT04346199 (AR, FR, DE, IT, ES, TR) | Reduce inflammation through BTK inhibition alleviates the severity of respiratory distress. | Treatment of patients with chronic lymphocytic leukemia and mantle cell lymphoma | |
| | Abivertinib | BTK inhibitor | PO, 100 mg qd for 7 d | NCT04528667 (US) NCT04440007 (US) | Reduce inflammation through BTK inhibition alleviates the severity of respiratory distress. | Treatment of patients with chronic lymphocytic leukemia and mantle cell lymphoma | |
| | Ibrutinib | BTK inhibitor | PO, 400 mg, bid, 7 d | NCT04439006 (US) | Reduce inflammation through BTK inhibition alleviates the severity of respiratory distress. | Treatment of patients with chronic lymphocytic leukemia and mantle cell lymphoma | |
| | Zanubrutinib | BTK inhibitor | PO, 320 mg/d | NCT04382586 (US) | Reduce inflammation through BTK inhibition alleviates the severity of respiratory distress. | Treatment of mantle cell lymphoma | |
| | Piclidenoson | A3 adenosine receptor agonist (A3AR) | PO, 12 mg, bid, < 28 d | NCT04333472 (IL) | modulate key signaling proteins, such as PI3K, PKA, PKB/Akt, IKK and NF-kB, resulting in de-regulation of the Wnt/β-catenin pathway and inhibition of inflammatory cytokine production. | Anti-inflammatory drug | |
| | Pentoxifylline | Phosphodiesterase-4 inhibitor | PO, 1200 mg/d | NCT04433988 (EG) | Anti-inflammatory role via reducing the production of proinflammatory cytokines such as TNF-a, IL-1 and IL-6. | Treatment of muscle pain in peripheral artery disease. | |
| | Ibudilast | Phosphodiesterase-4 inhibitor | PO,50 mg, bid, 7 d | NCT04429555 (CA) | Alleviate inflammation and apoptosis | Anti-inflammatory drug | [140] |
| | Losmapimod | MAPKs inhibitor | PO, 15 mg, bid, 14 d | NCT04511819 (US, BR, MX) | Inhibit p38 to attenuate COVID-19 infection. | Investigational drug for facioscapulohumeral dystrophy (FSHD) | [141] |
| | Progesterone | Hormone | SC, 100 mg, bid | NCT04365127 (US) | The anti-inflammatory actions of E2 on innate immunity includes the suppression of the production of proinflammatory cytokines, for example, IL-6, IL-1β, and TNF-α, by monocytes and macrophages (a major factor in the COVID-19 cytokine storm) and a strong inhibition of CCL2, thus preventing innate immune cells migration into inflamed areas, particularly neutrophils and monocytes. | sex hormone | [142] |
| | Isotretinoin | Synthetic retinoid derivative | PO,0.5 mg/kg/d, 14 d<br>INH, 0.2-4 mg/kg/d | NCT04353180 (EG) | Inhibit the overproduction of early response proinflammatory cytokines (IL- 6 and tumor necrosis factors alpha ) | Treatment severe acne | |
| | LAU-7b (fenretinide) | Synthetic retinoid derivative | PO, 300 mg, 7 d | NCT04417257 (CA) | Retinoic acid receptor agonists, suppress inflammation and the progression to cytokine storms that damage vital organs | Investigational drug for prostate cancer and other tumor | |

© 2020. Peng Y et al. Published online at http://www.aginganddisease.org/EN/10.14336/AD.2020.1124

| | | | | | | | |
|---|---|---|---|---|---|---|---|
| | Metformin | - | PO, 500 mg, bid | NCT04510194 (US) | Treatment of autoimmune disease and reduced macrophage cytokines synthesis. | Treatment of type 2 diabetes | |
| | Montelukast | Leukotriene receptor antagonist | PO, 10 mg, 60 d | NCT04389411 (CA) | Block the action of leukotriene D4 in the lungs resulting in decreased inflammation and relaxation of smooth muscle | Treatment and prevention of symptoms in asthma or allergies patients | |
| | DFV890 | NLRP3 inhibitor | PO and nasogastric administration, 14 d | NCT04382053 (DK, DE, HU, RU, ES) | NLRP3 inhibitor, modurate the innate immunity. | Investigational drug for Parkinson disease, Alzheimer disease, non-alcoholic steatohepatitis | |
| | Duvelisib | PI3K inhibitor | PO, 25 mg, bid, 10 d | NCT04372602 (US) NCT04487886 (US) | PI3K inhibitor, quell aberrant hyperactivtation of the innate immune system, preferentially polarize macrophages, reduce pulmonary inflammation, and limit viral persistence, thereby improving patient outcomes. | Treatmeng of chronic lymphocytic leukemia (CLL) or small lymphocytic lymphoma (SLL) after other treatments have failed | |
| | Apilimod Dimesylate | Class III PI3K inhibitor | PO, 125 mg, bid, 10 d | NCT04446377 (US) | Inhibitor of the class III PI kinase phosphatidylinositol-3-phosphate 5-kinase. Inhibit the toll-like receptor (TLR)-induced production of various cytokines, including IL-12 (IL-12) and IL-23, thereby preventing IL-12/IL-23-mediated immune responses. | Investigational drug for rheumatoid arthritis and severe Crohn's disease | |
| | Sirolimus | Immunosuppressant | PO, Initial dose of 6 mg, then 2 mg/d, 9 d | NCT04461340 (EG) NCT04371640 (US) NCT04482712 (US) | Macrolide compound, immunosuppressant functions | medicine for preventing the rejection of kidney transplants | |
| | Cyclosporine | Immunosuppressant | PO and IV, 9 mg/kg/day oral divided q12h, 3mg/kg/day by continuous IV infusion | NCT04412785 (US) NCT04392531 NCT04492891 | calcineurin inhibitor, used as an immunosuppressant medication . | Prevent organ rejection in people who have received a liver, kidney, or heart transplant. | |
| | TAK-671 | Ulinastatin-FC (UTI-Fc) fusion protein | IV | NCT04464460 (JP) | 1. Suppress the activation of polymorphonuclear leukocytes (e.g. neutrophils), macrophages and platelets;<br>2. Inhibit various serine proteases;<br>3. Inhibit polymorphonuclear leukocytes (e.g. neutrophils) and pro-inflammatory cytokines including IL-s (e.g. IL-1, IL-6 and IL-8) | Treatment of severe acute pancreatitis | [143] |
| | Fluoxetine | Serotonin reuptake inhibitor | PO, 20-60 mg, 14-60 d | NCT04377308 (US) | Selective serotonin reuptake inhibitor, | Antidepressant | |
| | Fluvoxamine | Serotonin reuptake inhibitor | PO, 100 mg, tid, 15 d | NCT04342663 (US) | Selective serotonin reuptake inhibitor, | Antidepressant | |
| | Tranilast | NLRP Inflammasome inhibitor | PO, 150 mg, bid | ChiCTR2000030002 | Tryptophan analogue; anti-allergic, inhibits NLRP3 in the inflammasome pathway. | Treatment of asthma, keloid scars, and hypertrophic scars | [113] |

© 2020. Peng Y et al. Published online at http://www.aginganddisease.org/EN/10.14336/AD.2020.1124

| | | | | | | | |
|---|---|---|---|---|---|---|---|
| | Colchicine | NLRP Inflammasome inhibitor | PO, 0.5 mg, bid for 3 d; then 0.5 mg qd for 27 d | NCT04322565 (IT) NCT04363437 (US) NCT04367168 (MX) | Inhibit inflammasome NLP3 and microtubule formation and associations to limite the myocardial necrosis and pneumonia development. | Preventation or treatment of gout attacks | [144] |
| | Azoximer bromide | Immunomodulator | IV 3 day, followed by 12 mg, 10 times | NCT04381377 (RU) | A N -oxidized polyethylene–piperazine derivative. | Investigational drug for improving secondary immunodeficient states | |
| | OP-101 (Dendrimer N-acetylcysteine) | Immunomodulator | IV, 2 mg/kg | NCT04458298 (US) | Reduce proinflammatory cytokines | - | |
| | EDP1815 | Monoclonal microbe | PO, 550 mg | NCT04488575 (US) | Harnes the connections between intestinal mucosal immunology and systemic inflammation for broad inflammation resolution without immunosuppression. | Investigational drug for moderate psoriasis | |
| | Dialyzable Leukocyte Extract | Immunomodulator | PO, 2 mg for 14 d, followed by 2 mg per week for 3 w, 35 d | NCT04379479 (MX) | Complexes consisting of a large number of low molecular weight substances, have antigen-independent immunorestorative properties in vitro. | - | |
| | Nebulized Platelet Lysate | Platelet lysate | INH, 2 mL, 42 d | NCT04487691 (US) | Reduce inflammation and stimulates bronchial tree healing | - | |
| | CAP-1002 | Allogeneic Cardiosphere-Derived Cells | IV | NCT04338347 (US) | Allogeneic Cardiosphere-Derived Cells | - | |
| | Autologous Non-Hematopoietic Peripheral Blood Stem Cells | Autologous non-hematopoietic peripheral blood stem cells | INH | NCT04473170 (AE) | The NHPBSC were characterized as CD90+, CD133+, Oct-4+ (pluripotent markers), and CD45-, CD71-, based on multiparameter flow cytometry. | Treatment of autoimmunity | [145] |
| | Allocetra-OTS | Cell-based therapeutic composed of donor early apoptotic cells | IV, 140 ×10^6 ± 20% cells / kg | NCT04513470 (IL) | Harness the naturally occurring activity of early apoptotic cells to induce an immuno-modulated state. | Prevention of graft vs. host disease (GvHD) in patients receiving human stem cell transplantation (HSCT) | [146] |
| | Autologous adipose-derived stem cells | Mesenchymal Stem Cell Therapy | Autologous adipose-derived stem cells: IV, 200 million celss, 3 doses per 3 days | NCT04428801 (US) NCT04349631 (US) | (Autologous adipose-derived mesenchymal stem cells) | Treatment of inflammatory and degenerative diseases | |
| | RAPA-501-Allo | Reprogrammed T cell | IV, 40, 160 × 10^6 cells | NCT04482699 (US) | Anti-inflammatory via modulate the TREG and Th2 pathways | Allogeneic off-the-shelf RAPA-501 cells | |
| | CSTC-Exo | Specific T-cells | INH, 2.0 × 10^8 nano vesicle / 3 ml; | NCT04389385 (TR) | The COVID-19 specific fragment peptides activate specific T-cells and stimulate the secretion of potent mediators including IFN gamma in forms of exosomes. | - | |
| | CK0802 | T-Regulatory Cell Infusion | IV, 1×10 ^ 8 cells | NCT04468971 (US) | (Cryopreserved Cord Blood Derived T-Regulatory Cell Infusions) | - | |
| | CAStem | Immunity- and matrix-regulatory cells | IV, 3-5×10^7 cell/kg | NCT04331613 (CN) | An injectable product composed of immunity- and matrix-regulatory cells | - | |

© 2020. Peng Y et al. Published online at http://www.aginganddisease.org/EN/10.14336/AD.2020.1124

| | | | | | | | |
|---|---|---|---|---|---|---|---|
| | Stem Cell Educator-Treated Mononuclear Cells Apheresis | Stem Cell Educator-Treated Mononuclear Cells Apheresis | IV | NCT04299152 (CN) | Restore immune balance and homeostasis in inflammation-associated diseases. | - | |
| | Cannabidiol | Active cannabinoid | PO, 150 mg, bid, 14 d | NCT04467918 (BR) | Reduce the cytokine storm and excessive lung inflammation | Treatment of Lennox-Gastaut syndrome and Dravet syndrome | |
| **Treament of Acute respiratory distress syndrome** | Captopril | Angiotensin-converting enzyme (ACE) inhibitor | INH, 25 mg | NCT04355429 (FR) | Decrease infectivity of COVID-19 by modulating the expression of ACE2. | Treatment of hypertension and some types of congestive heart failure. | |
| | Ramipril | Angiotensin-converting enzyme (ACE) inhibitor | PO, 2.5 mg/d, 14 d | NCT04366050 (US) | Lower IL-6 and peak viral load | Treatment of high blood pressure, heart failure, and diabetic kidney disease | [147] |
| | Losartan | angiotensin II receptor (type AT1) antagonis | PO, 25 mg, qd | NCT04312009 (US) NCT04311177 (US) NCT04328012 (US) | Inhibition of Viral Macrodomain of COVID-19 and Human TRPM2. | Treatment of hypertension | [148] |
| | Telmisartan | Angiotensin II receptor (type AT1) antagonis | PO, 40 mg, bid, 14 d | NCT04359953 (FR) NCT04356495 (FR) | a second class RAAS inhibitors, do not affect ACE2 levels. | Treatment of hypertension | [149] |
| | Angiotensin peptide | Angiotensin peptide (1-7) derived plasma | IV | NCT04375124 (TR) NCT04332666 (NL) NCT04401423 (US) | Improve the mortal hyper-inflammation state by supply angiotensin peptide. | - | [150] |
| | BIO101 | MAS activator | PO, 350 mg, bid, < 28 d | NCT04472728 (US, BE, BR, FR) | Activate the MAS receptor, a key component of the protective arm of the RAS | Investigational drug for sarcopenia | |
| | C21 | Nonpeptide angiotensin II type 2 receptor agonist | < 28 d 100 mg, bid, 7 d | NCT04452435 (IN, US) | Antifibrotic effects | Investigational drug of fibrosis | |
| | | | INH, 20 mcg, 5 d | NCT04445246 (QA) | | | |
| | Nitric Oxide Gas Inhalation Therapy | Targeted pulmonary vasodilator | INH, NO delivered through a non-invasive CPAP system, concertation maintained between 140 and 180 ppm, 20-30 min, twice a day, 14 d | NCT03331445 (CA) NCT04290858 (CN) NCT04290871 (CN) NCT04305457 (US) NCT04306393 (US) NCT04337918 (CA) NCT04358588 (US) NCT04383002 (CA) NCT04398290 (US) NCT04421508 (US) NCT04443868 (US) NCT04456088 (US) NCT04476992 (RU) | NO delivered through a non invasive CPAP system, concertration maintained between 140 and 180 ppm, 20-30 min, twice a day. Vasodilation in the lung. | Treatment of respiratory failure in premature babies | [151] |

© 2020. Peng Y et al. Published online at http://www.aginganddisease.org/EN/10.14336/AD.2020.1124

# SUPPLEMENTARY DATA

| | | | | | | | |
|---|---|---|---|---|---|---|---|
| | RESP301 | Targeted pulmonary vasodilator: Nitric Oxide generating solution | INH, 10 d | NCT04460183 (GB) | NO delivered through a non-invasive CPAP system, concertation maintained between 140 and 180 ppm, 20-30 min, twice a day. Vasodilation in the lung. | Treatment of respiratory failure in premature babies | |
| | Sodium Nitrite | Targeted pulmonary vasodilator, (Methylene blue, vitamin C, N-acetyl cysteine) | IV, 30 mg/mL intravenous infusion | NCT04401527 (US) | Enhance lung airway epithelial cells wound healing | - | |
| | Methylene Blue | Targeted pulmonary vasodilator, (Methylene blue, vitamin C, N-acetyl cysteine) | - | NCT04370288 (IR) | an inhibitor of nitric oxide synthase and guanylate cyclase | Treatment of methemoglobinemia | |
| | GLS-1200 | Targeted pulmonary vasodilator | 1 mL per nostril, tid, ISIN | NCT04408183 (US) | Stimulate nasal production of nitric oxide to inhibit coronavirus activity. | Treatment of respiratory failure in premature babies | |
| | VIB7734 | Anti-ILT7 antibody | SC | NCT04526912 (US) | Anti-ILT7 | Investigational drug for autoimmune diseases | |
| | vadadustat | HIF prolyl-hydroxylase inhibitor | PO, 14 d | NCT04478071 (US) | Improve the level of HIF in endothelial damage via inhibit HIF prolyl-hydroxylase | Treatment of anemia secondary to chronic kidney disease | [152] |
| | Iloprost | Prostacyclin | IV, 1 ng/kg/min, 3 d | NCT04420741 (DK) | Improve endothelial function and integrity | Treatment of pulmonary arterial hypertension (PAH), scleroderma, Raynaud's phenomenon | |
| | Melatonin | Hormone | PO, 10-30 mg, tid, 14 d | NCT04474483 (US) NCT04531748 (US) NCT04353128 (ES) | An anti-oxidative and anti-inflammatory agent counters acute lung injury (ALI)/acute respiratory distress | Treatment of insomnia in children and adolescents | |
| | Ramelteon | Melatonin Agonist | PO, 8 mg, 10 d | NCT04470297 (BR) | - | Sleep agent medication | |
| | Thalidomide | Angiogenesis inhibitor and immunomodulator | PO, 100 mg, qd, 14 d | NCT04273529 (CN) NCT04273581 (CN) | 1.Anti-inflammatory; 2.Anti-fibrotic; 3. Anti-angiogenes; 4. Immune regulation effects. | Treatment of cancer | [153] |
| | Bevacizumab | Anti VEGF recombinant humanized monoclonal antibody, | IV, 7.5 mg/kg, < 28 d | NCT04305106 (CN) NCT04275414 (CN) NCT04344782 (FR) | Vascular endothelial growth factor (VEGF) is known as the most potent inducing factors to increase vascular permeability. | Treatment of tumor | [154] |
| | Garadacimab | FXIIa inhibtor | IV, 75, 200, 600 mg | NCT04409509 (US) | Factor XIIa Antagonist Monoclonal Antibody to avoid edema formation | Investigational drug for prevention of bradykinin-mediated angioedema | |
| | Conestat alfa | C1 esterase Inhibitor | IV, 8400 U, followed by 4200 U every 8 hours | NCT04414631 (CH) | 1. Dampen uncontrolled complement activation and collateral lung damage; 2. Reduce capillary leakage and subsequent pulmonary edema by direct inhibition of KK system | Treatment of acute attacks of hereditary angioedema (HAE) due to C1 esterase inhibitor deficiency | |
| | Sildenafil | Phosphodiesterase inhibitor | PO, 20-50 mg, tid | NCT04304313 (CN) | Treat high altitude pulmonary edema. | Treatment of sexual dysfunction, pulmonary hypertension, and investigational drug | [155] |

© 2020. Peng Y et al. Published online at http://www.aginganddisease.org/EN/10.14336/AD.2020.1124

| | | | | | | | |
|---|---|---|---|---|---|---|---|
| | | | | | | for high-altitude pulmonary edema | |
| | lanadelumab | Plasma kallikrein antibody | IV, 300 mg | NCT04422509 (NL) NCT04460105 (US) | Target plasma kallikrein (pKal) in order to promote prevention of angioedema in patients with hereditary angioedema | Investigational drug for acute angioedema attacks in hereditary angioedema patients | |
| | Zavegepant | CGRP receptor antagonist | ISIN, 10 mg, tid, 14 d | NCT04346615 (US) | CGRP receptor antagonist. CGRP might contribute to abnormal vascular reactivity observed in acute lung injury | Treatment of acute migraine | [156] |
| | Mesenchymal Stem Cell | Mesenchymal stem cell therapy | IV, MSC: 3x10^7 cells on Day 0, Day 3 and Day 6 | NCT04252118 (CN) NCT04400032 (CA) NCT04525378 (BR) | Inhibit the overactivation of the immune system and promote endogenous repair by improving the lung microenvironment. | Treatment of inflammatory and degenerative diseases | |
| | | | IV, NestCell® MSC: 1x10^6 cells/kg on Day 1, Day 3 and Day 7 | NCT04315987 (BZ) | (produced by Cellavita ) | | [157] |
| | | | Wharton's Jelly-Mesenchymal Stem Cells: IV, 1x10^6 cells/kg on Day 1, Day 4 and Day 7 | NCT04456361 (MX) NCT04333368 (FR) NCT04390152 (CO) | | | |
| | | | Remestemcel-L: IV, 2 ×10 ^ 6 MSC / kg | NCT04371393 (US) NCT04456439 (CH) | | Treatment of inflammatory and degenerative diseases | |
| | | | SBI-101: IV, 2.5 × 10 ^ 9 cells | NCT04445220 (US) | | Treatment of inflammatory and degenerative diseases | |
| | | | Dental pulp MSC: IV, 1x10^6 cells/kg on Day 1, Day 3 and Day 7 | NCT04302519 (CN) | (Dental pulp drived mesenchymal stem cell) | | |
| | | | Adipose tissue derived MSC: IV, 0.5-1.5 million/kg | NCT04348461 (ES) NCT04352803 (US) | (Allogeneic adipose-derived mesenchymal stem cells) | | |
| | | | Hope Biosciences-ad MSC: IV | NCT04348435 (US) | (Allogeneic adipose-derived mesenchymal stem cells) | | |
| | | | Allogenic pooled olfactory mucosa derived MSC: IV | NCT04382547 (BY) | (Allogenic pooled olfactory mucosa-derived mesenchymal stem cells) | | |
| | | | MSC - KI-MSC-PL-205: IV | NCT04447833 (SE) | (Bone marrow derived mesenchymal stromal stem cells) | | |
| | | | BM-Allo.MSC: IV | NCT04397796 (US) | (Bone marrow derived mesenchymal stromal stem cells) | | |
| | Human Umbilical Cord Mesenchymal Stem Cells | Mesenchymal stem cell therapy | IV, 0.5x10^6 cells/kg on Day 1, Day 3, Day 5 and Day 7 | NCT04269525 (CN) NCT04366271 (ES) NCT04490486 (US) | Inhibit the overactivation of the immune system and promote endogenous repair by improving the lung microenvironment. | Treatment of inflammatory and degenerative diseases | |
| | | | MAS825: IV, 0.5 × 10^6 cells/kg, Day 1, 2, 3, 7 | NCT04293692 (CN) NCT04382651 (US) | | Investigational drug for inflammatory diseases | |
| | | | PLX-PAD: IM | NCT04389450 (US) | (allogeneic ex vivo expanded placental mesenchymal-like adherent stromal cells） | | |

© 2020. Peng Y et al. Published online at http://www.aginganddisease.org/EN/10.14336/AD.2020.1124

# SUPPLEMENTARY DATA

| | | | | | | | |
|---|---|---|---|---|---|---|---|
| | MSCs-derived exosomes | Mesenchymal stem cell therapy | INH, 2.0x10^8 nano vesicles/3 ml at Day 1, Day 2, Day 3, Day 4, Day 5 | NCT04276987 (CN) | Inhibit the overactivation of the immune system and promote endogenous repair by improving the lung microenvironment. | Treatment of inflammatory and degenerative diseases | |
| | Descartes 30 | Mesenchymal stem cell therapy | IV | NCT04524962 (US) | Mesenchymal Stem Cells or MSCs RNA-engineered to secrete a combination of DNases. | - | |
| | Human umbilical cord derived CD362 enriched MSCs | Mesenchymal stem cell therapy | IV | NCT03042143 (GB) | Human umbilical cord derived CD362 enriched MSCs.<br>Inhibit the overactivation of the immune system and promote endogenous repair by improving the lung microenvironment. | Treatment of inflammatory and degenerative diseases | |
| | MultiStem | MultiStem cell therapy | IV | NCT04367077 (US) | - | Investigational drug for diseases and conditions in the neurological, inflammatory and immune | |
| | Bardoxolone methyl | Nrf2 Pathway Activator | PO, 20 mg, qd | NCT04494646 (US) | An activator of the Nrf2 pathway and an inhibitor of the NF-κB pathway. | Investigational drug for COVID-19 and rheumatoid arthritis | |
| | ATYR1923 | NRP2 modulator | IV, 1 or 3 mg/kg | NCT04412668 (US) | Inhibit cytokines and chemokines involved in the regulation of inflammatory and fibrotic responses. | Investigational drug for interstitial lung disease | |
| | BLD-2660 | Dimeric calpain (CAPN) 1, 2, and 9 inhibitor | PO, 150 mg, bid | NCT04334460 (US) | Reverse the overactivation of the immune system and progression of fibrosis in several organs. | Treatment of fibrosis | |
| | PB1046 | Analog of the neuropeptide vasoactive intestinal peptide | SC, 10, 40 and 100 mg per week | NCT04433546 (US) | Long-acting, sustained release human vasoactive intestinal peptide (VIP) analogue. Attenuate inflammation and fibrosis, modulate of inflammatory cell activity, based on the known mechanisms of VIP | - | |
| | TD-139 | Galectin-3 inhibitor | INH, 10 mg, bid in Day 1, 2; 10 mg, qd for 12 days | NCT04473053 (GB) | Modulate the fibrogenic response to tissue injury | Investigational drug for idiopathic pulmonary fibrosis | |
| | Nintedanib | RTK inhibitor | PO, 150 mg, bid, 56 d | NCT04338802 (CN) | Reduce fibrotic damage driven by immune dysregulation. | Treatment of idiopathic pulmonary fibrosis | |
| | Pirfenidone | NCT04282902 (CN) | PO, 200 mg, tid, > 28 d | NCT04282902 (CN) | Anti-SOD activity can effectively inhibit IL-1beta and IL-4, and can open the prevention mode of pulmonary interstitial fibrosis. | Treatment of idiopathic pulmonary fibrosis | |
| | Tradipitant | Neurokinin 1 antagonist | PO, 85 mg, bid | NCT04326426 (CN) | Block the neurokinin-1 receptor (NK1R), which involved in a range of neuroinflammatory processes that leads to significant lung injury. | Investigational drug for for chronic pruritus (itchiness) in atopic dermatitis | [158] |
| | AVM0703 | Immunomodulator | IV, 10 mg/mL | NCT04366115 (US) | Supercharge immune cells (natural killer T-cells, gamma delta T cells and dendritic cells) | Investigational drug for B-cell lymphoma | |
| | Ifenprodil | N-methyl-D-Aspartate (NDMA) inhibitor that is specific for the NR2B | PO, 20 mg, tid | NCT04382924 (US) | The activation of T cells and cytokine release can be blocked in vitro by the addition of Ifenprodil | Investigational drug for idiopathic pulmonary fibrosis | |

© 2020. Peng Y et al. Published online at http://www.aginganddisease.org/EN/10.14336/AD.2020.1124

| | | | | | | | |
|---|---|---|---|---|---|---|---|
| | | subunit of the NMDA Recepto | | | | | |
| | Aviptadil | Synthetic version of Vasoactive Intestinal Polypeptide | IV, 50 pmol, 100 pmol, 150 pmol/kg/hr | NCT04311697 (US)<br>NCT04453839 (US)<br>NCT04360096 (US) | 1. Prevent NMDA-induced caspase-3 activation in the lung;<br>2. Inhibits IL6 and TNFa production, protects against HCl-induced pulmonary edema;<br>3. Restore barrier function at the endothelial/alveolar interface and thereby protects the lung and other organs from failure. | Treatment of erectile dysfunction | |
| | Ibuprofen | Nonsteroidal anti-inflammatory drug | PO, 200 mg | NCT04334629 (GB) | Improve fever and inflammation | Pain killer | |
| | Indomethacin | Nonsteroidal anti-inflammatory drug | PO, 50 mg, pid, 14 d | NCT04344457 (US) | Improve fever and inflammation | Pain killer | |
| | Estradiol | Hormone therapy | TOPp, 100 µg/d, 7 d | NCT04359329 (US) | Estrogen, immune modulation; Reduce the damaging effects of the virus on the lung and symptom severity. | Estrogen steroid hormone | |
| | Sargramostim | Yeast-derived recombinant humanized granulocyte-macrophage colony stimulating factor (rhuGM-CSF) | INH and IV, 125 mcg bid for 5 d, then 125mcg/m2 by by intravenous for 5d | NCT04326920 (BE)<br>NCT04411680 (US)<br>NCT04400929 (SG) | Stimulus to restore immune homeostasis in the lung. | Activating mature granulocytes and macrophages | |
| | Human Amniotic Fluid (acellular) | - | 3 mL, INH and 6 mL, IV, 5 d | NCT04319731 (US) | Reduce inflammation and fibrosis. | Treatment of tissue injury | |
| | XC221 | - | PO, 100 mg, bid | NCT04487574 (RU) | - | Investigational drug for Resp. Syncytial Virus | |
| **Anticoagulant therapy** | Aspirin | Thrombolytic medication | PO, 81 mg, bid, 45 d | NCT04498273 (US)<br>NCT04365309 (CN)<br>NCT04466670 (BR) | 1. Inhibit virus replication;<br>2. Anticoagulant;<br>3. Anti-inflammatory. | Treatment of a number of conditions, including fever, pain, rheumatic fever, and inflammatory conditions | |
| | Prasugrel | P2Y12 inhibitors | PO, 60 mL, 15 d | NCT04445623 (IT) | P2Y12 inhibitor for the prevention of thrombosis in the pulmonary circulation and attenuation of inflammation. | Drug used to prevent formation of blood clots | |
| | epoprostenol | Prostacyclin analogue | INH, 10 d | NCT04452669 (US) | 1. Vasodilate pulmonary and systemic arterial vascular beds;<br>2. Inhibit platelet aggregation | Treatment of pulmonary arterial hypertension | |
| | Crizanlizumab | P-selectin antibody | IV, 5 mg/Kg | NCT04435184 (US) | Decrease inflammation by binding to P-selectin, blocking leucocyte and platelet adherence to the vessel wall. | Drug to prevent vaso-occlusive crisis in patients with sickle cell anemia | |
| | Alteplase | Thrombolytic medication | IV, 100 mg or 200 mg | NCT04357730 (US) | Use of plasminogen activators to limit ARDS progression | Treatment of acute ST elevation myocardial infarction and pulmonary embolism associated with low blood pressure, acute ischemic stroke, and | |

# SUPPLEMENTARY DATA

| | | | | | | | |
|---|---|---|---|---|---|---|---|
| | | | | | | blocked central venous access devices (CVAD) | |
| | Defibrotide | Thrombolytic medication | IV, 25 mg/kg/6 h, 7 d | NCT04335201 (IT) | A mixture of single-stranded oligonucleotides, increases t-PA function and decrease plasminogen activator inhibitor-1 activity. | Treatment of veno-occlusive disease | |
| | Bivalirudin | Thrombolytic medication | IV, 0.15-0.2 mg/kg/hr | NCT04445935 (QA) NCT04366921 (AT, BE, CS, DK, FR, DE, GR, HU, IL, IT, LT, NL, PT, PL, RU, ES, CH, SE, GB) | Direct thrombin inhibitor | Thrombolytic medication | [159] |
| | Dipyridamole | Thrombolytic medication | PO, 100 mg, 14 d | NCT04391179 (US) | Decreas concentrations of D-dimers, increase lymphocyte and platelet recovery in the circulation, and markedly improve clinical outcomes | Drug for antiplatelet | |
| | Heparin | Thrombolytic medication | INH, 40 or 60 mg/d | NCT04466670 (BR) | Reduce formation of microthrombi and clinically improve relevant disseminated intravascular coagulation | Anticoagulant | |
| | | | IV, 4000 IU / 24h | NCT04505774 (US) NCT04393805 (IT) | | | |
| | | | SC, 1 mg/kg | NCT04485429 (BR) NCT04372589 (US) NCT04528888 (IT) | | | |
| | Tinzaparin | Thrombolytic medication | SC | NCT04412304 (SE) | Reduce formation of microthrombi and clinically improve relevant disseminated intravascular coagulation | Antithrombotic drug | |
| | Enoxaparin | Thrombolytic medication | SC, 40 mg | NCT04366960 (IT) NCT04466670 (BR) NCT04528888 (IT) | Reduce formation of microthrombi and clinically improve relevant disseminated intravascular coagulation | Anticoagulant | |
| | Dociparastat | Thrombolytic medication | IV, 4 mg/kg, 8 d | NCT04389840 (US) | A glycosaminoglycan derived from porcine heparin. | - | |
| | Sulodexide | Two-compound drug: glycosaminoglycans (20 %) + heparin (80 %) | PO, 500 LSU, 21 d | NCT04483830 (MX) | Reduce formation of microthrombi and clinically improve relevant disseminated intravascular coagulation | Treatment of thromboembolic diseases | |
| | Rivaroxaban | Thrombolytic medication | PO, 10 mg, 21 d | NCT04504032 (US) NCT04416048 (DE) NCT04394377 (BR) | Inhibit both free Factor Xa and Factor Xa bound in the prothrombinase complex. Reduce formation of microthrombi and clinically improve relevant disseminated intravascular coagulation | Treatment and prevention of stroke, heart attack, deep vein thrombosis and pulmonary embolism | |
| | Apixaban | Thrombolytic medication and blood thinner | PO, 2.5 or 5.0 mg, bid, 45 d | NCT04498273 (US) NCT04512079 | Prevent of thrombosis, pulmonary hemorrhage and high prevalence of VTE. | Treatment and prevention of stroke, heart attack, deep vein thrombosis and pulmonary embolism | |
| | LB1148 (tranexamic acid) | Hemostatic agent | PO, 7.5 g | NCT04390217 (US) | Stop bleeding events that result from disseminated intravascular coagulation | Antifibrinolytics | |
| **Vaccine** | BBV159 | Inactivated vaccine | IM, 0.5 mL, twice | NCT04471519 (IN) | Whole-Virion Inactivated SARS-CoV-2 Vaccine | Investigational vaccine for COVID-19 | |

| | | | | | | | |
|---|---|---|---|---|---|---|---|
| | CoronaVac | Inactivated Vaccine | IM, 600 SU / 0.5ml or 1200 SU / 0.5ml, day 0 and day 14 | NCT04352608 (CN) | Inactivated Vaccine | Investigational vaccine for COVID-19 | [160] |
| | BBIBP-CorV | Inactivated Vaccine | IM, 2 μg, 4 μg, or 8 μg on Day 0 and day | ChiCTR2000032459 | Inactivated Vaccine | Investigational vaccine for COVID-19 | [161] |
| | MF59 adjuvanted SARS-CoV-2 Sclamp vaccine | Inactivated vaccine | IM, 5, 15 and 45 mcg | NCT04495933 (AU) | MF59 adjuvanted SARS-CoV-2 Sclamp vaccine | Investigational vaccine for COVID-19 | [162] |
| | Inactivated SARS-CoV-2 Vaccine | Inactivated vaccine | SC, 50 U | NCT04412538 (CN) NCT04508075 (IN) | Inactivated SARS-CoV-2 Vaccine (Vero cell) | Investigational vaccine for COVID-19 | |
| | Covax-19™ | Subunit vaccine | Spike antigen (25ug) + 15 mg Advax-2 adjuvant, IM | NCT04428073 (US) | COVID19 recombinant spike protein with Advax-SM adjuvant | Investigational vaccine for COVID-19 | |
| | recombinant SARS-CoV-2 vaccine (Sf9 Cell) | Subunit vaccine | IM, 20 μg / 0.5 ml | NCT04530656 (CN) | Insect cell line Sf9 expressed vaccine | Investigational vaccine for COVID-19 | [163] |
| | AdimrSC-2f | Subunit vaccine | IM | NCT04522089 (TW) | The baculovirus-insect cells expression system to amplify and purify the recombinant receptor binding domain (RBD) of SARS-CoV-2 spike (S) protein | Investigational vaccine for COVID-19 | |
| | Intramuscular Vaccine | Subunit vaccine | IM, 3.75 μg, 7.5 μg and 15 μg VLP | NCT04450004 (CA) | Coronavirus-Like Particle COVID-19 Vaccine | Investigational vaccine for COVID-19 | |
| | MVC-COV1901 | Subunit vaccine | IV | NCT04487210 (TW) | MVC-COV1901 is formulated in the different dosages of Spike (S) protein with CpG 1018 and aluminum content as adjuvant | Investigational vaccine for COVID-19 | |
| | AP TP Vaccine | Subunit vaccine | IV, 0.1mg | NCT03348670 (US) NCT03305341 (US) | COVID-19 Therapeutic Vaccine - Nucleocapsid-GM-CSF Protein Lactated Ringer's Injection | Investigational vaccine for COVID-19 | |
| | KBP-COVID-19 | Subunit vaccine | IM | NCT04473690 (US) | RBD-based vaccine | Investigational vaccine for COVID-19 | |
| | SCB-2019 | Subunit vaccine | IM, 3 - 30 μg, twice | NCT04405908 (AU) | S-Trimer subunit vaccine | Investigational vaccine for COVID-19 | |
| | Recombinant Novel Coronavirus Vaccine | Subunit vaccine | IM, 25 or 50 μg | NCT04445194 (CN) NCT04466085 (CN) | Recombinant new coronavirus vaccine (CHO cell) | Investigational vaccine for COVID-19 | |
| | EpiVacCorona | Subunit vaccine | IM, 0.5 mL | NCT04527575 (RU) | The vaccine relies on chemically synthesized peptide antigens of SARS-CoV-2 proteins, conjugated to a carrier protein and adsorbed on an aluminum-containing adjuvant (aluminum hydroxide). | Investigational vaccine for COVID-19 | |
| | Ad5-nCoV | Adenovirus vaccine vector | IM, 5x10^10 U; 1x10^11 U; 1.5x10^11 U | NCT04313127 (CN) NCT04398147 (CA) | Efficient Adenovirus vector to express viral Spike protein. | Investigational vaccine for COVID-19 | [164] |
| | CTII-nCoV | Adenovirus vaccine vector | IM, 1×10^11vp; 5×10 ^ 10vp | NCT04341389 (CN) | Efficient Adenovirus vector to express viral Spike protein. | Investigational vaccine for COVID-19 | |
| | Ad26.COV2.S (JNJ-78436735) | Adenovirus vaccine vector | IM, 1×10^11 virus particles (vp) | NCT04505722 (US) NCT04509947 (JP) | Efficient Adenovirus vector to express viral Spike protein. | Investigational vaccine for COVID-19 | [165] |
| | Gam-COVID-Vac | Adenovirus vaccine vector | IM, 0.5 mL | NCT04436471 (RU) NCT04437875(RU) | Adenoviral-based vaccine against SARS-CoV-2 | Investigational vaccine for COVID-19 | |

| | | | | | | | |
|---|---|---|---|---|---|---|---|
| | | | | NCT04530396 (RU) | | | |
| | GRAd-COV2 | Adenovirus vaccine vector | IM, 0.5-2 × 10^11 vp | NCT04528641 (IT) | Adenoviral-based vaccine against SARS-CoV-2 | Investigational vaccine for COVID-19 | |
| | ChAdOx1 nCoV-19 (AZD1222) | Adenovirus vaccine vector | IM, 5×10^10 cell | NCT04324606 (BR) NCT04400838 (BR) NCT04444674 (ZA) | Chimpanzee adenovirus-vectored vaccine (ChAdOx1 nCoV-19) expressing the SARS-CoV-2 spike protein compared with a meningococcal conjugate vaccine | Investigational vaccine for COVID-19 | |
| | COVID-19 aAPC Vaccine | Lentiviral vector system | SC, 5x10^6 U, 3 times | NCT04299724 (CN) | Efficient lentiviral vector system (NHP/TYF) to express viral proteins and immune modulatory genes. | Investigational vaccine for COVID-19 | [166] |
| | LV-SMENP-DC vaccine/ antigen-specific CTLs | Lentiviral vector system | vaccineaccine: SC or IV, 5x10^6 ; CTLs: 1x10^8 | NCT04276896 (CN) | Efficient lentiviral vector system (NHP/TYF) to express viral proteins and immune modulatory genes. | Investigational vaccine for COVID-19 | [166] |
| | TMV-083 | Measles vector-based vaccine | IM, 2.0 mg, 2-4 times | NCT04497298 (BE, FR) | - | Investigational vaccine for COVID-19 | |
| | V591 | Measles virus vector vaccine | IM | NCT04498247 (US) | SARS-CoV-2 vaccine candidate that uses a measles virus vector platform | Investigational vaccine for COVID-19 | |
| | SARS-CoV-2 Recombinant Spike Protein Nanoparticle Vaccine (NVX-CoV2373) | Subunit vaccine | IM, 25 µg | NCT04368988 (AU) | SARS-CoV-2 Recombinant Spike Protein | Investigational vaccine for COVID-19 | |
| | INO-4800 | DNA vaccine | IM, 1 mg; 1.0 mg twice | NCT04336410 (US) NCT04447781 (KR) | DNA plasmid encoding S protein delivered by electroporation. | Investigational vaccine for COVID-19 | [164] |
| | AG0301-COVID19 | DNA vaccine | IM, 1.o or 2.0 mg, 2 times | NCT04463472 (JP) | - | Investigational vaccine for COVID-19 | |
| | AG0302-COVID19 | DNA vaccine | IM, 2.0 mg, 2-4 times | NCT04527081 (JP) NCT04463472 (JP) | - | Investigational vaccine for COVID-19 | |
| | GX-19 | DNA vaccine | IM | NCT04445389 (KR) | DNA vaccine expressing SARS-CoV-2 S-protein antigen | Investigational vaccine for COVID-19 | |
| | MRT5500 | mRNA-based vaccine | IM, Day 0 and Day 28 | - | RNA Vaccine | Investigational vaccine for COVID-19 | [167] |
| | BNT162 | mRNA-based vaccine | IM, 0.5 mL | NCT04368728 (US) NCT04523571 (CN) NCT04380701 (GE) | RNA Vaccine (BNT162a1, BNT162b1, BNT162b2 and BNT162c2): mRNA-based vaccine, which encodes for vitral spike (S) protein. | Investigational vaccine for COVID-19 | [168] |
| | CVnCoV | mRNA-based vaccine | IM, 6 µg on Day 1 and Day 29 | NCT04515147 (DE) | mRNA-based vaccine, which encodes for vitral spike (S) protein. | Investigational vaccine for COVID-19 | |
| | mRNA-1273 | mRNA-based vaccine | IM, 100 mcg on Day 1 and Day 29 | NCT04283461 (US) NCT04299724 (CN) NCT04470427 (US) | Novel lipid nanoparticle (LNP)-encapsulated mRNA-based vaccine, which encodes for vitral spike (S) protein. | Investigational vaccine for COVID-19 | [169] |
| | ARCT-021 | mRNA-based vaccine | IM, 0.5 mL | NCT04480957 (SG) | Vaccine comprising a self-replicating (replicon) mRNA that encodes for the prefusion spike protein of 2019-nCoV formulated in a lipid nanoparticle (LNP). | Investigational vaccine for COVID-19 | |
| | DeltaRex-G | mRNA-based vaccine | IV, 1 × 10^11 cfu/dose | NCT04378244 (US) | DeltaRex-G Retroviral Vector Encoding a Cyclin G1 Inhibitor | Investigational vaccine for COVID-19 | |

© 2020. Peng Y et al. Published online at http://www.aginganddisease.org/EN/10.14336/AD.2020.1124

# SUPPLEMENTARY DATA

| | | | | | | | |
|---|---|---|---|---|---|---|---|
| | SARS-CoV-2 mRNA vaccine | mRNA-based vaccine | - | ChiCTR2000034112 | - | Investigational vaccine for COVID-19 | |
| **Auxiliary medication** | Bromhexine | Expectorant | PO, 8 mg, per 8h, 60 d | NCT04340349 (MX) NCT04405999 (RU) | Support the body's mechanisms for clearing mucus from the respiratory tract | Treatment of respiratory disorders associated with viscid or excessive mucus | |
| | N-acetylcysteine | Expectorant | PO, 600 mg | NCT04419025 (GB) | mucus-dissolving therapy | Treatment of paracetamol (acetaminophen) overdose, and thick mucus in individuals with cystic fibrosis or chronic obstructive pulmonary disease. | |
| | Dornase Alpha | Recombinant human deoxyribonuclease I (rhDNase) | INH, 2.5 mg, bid, 7 d | NCT04432987 (TR) NCT04402944 (US) NCT04359654 (GB) | Dornase alfa hydrolyzes the DNA present in sputum/mucus of cystic fibrosis patients and reduces viscosity in the lungs, promoting improved clearance of secretions. | Investigational drug for cystic fibrosis | |
| | Lucinactant | Synthetic surfactant | IV, 80 mg/kg | NCT04389671 (US) | A synthetic, peptide-containing endogenous pulmonary surfactant, lowers the surface tension at air-liquid interface of the alveolar surfaces during respiration. | Treatment of infant respiratory distress syndrome | |
| | CUROSURF® | Poractant alfa | ET: 30 mg/kg | NCT04502433 (GB) NCT04384731 (FR) | Surfactant covers the alveolar surface to reduce the work of breathing and prevent the lungs from collapsing. | - | |
| | Decidual Stromal Cells | Decidual Stromal Cells | IV, 1×10 ^ 6 cells/kg, Day1, Day 5 | NCT04451291 (CA) | Reduce lung inflammation and then time that patients require help with breathing (mechanical ventilation). | - | |
| | Almitrine | Respiratory stimulant | IV, 2 µg/kg/min for 5 d | NCT04357457 (FR) | Reduce intrapulmonary shunt by enhancement of hypoxic pulmonary vasoconstriction in combination with inhaled nitric oxide (iNO). | Treatment of chronic obstructive pulmonary disease | |
| | S-Nitrosylation Therapy | S-nitrosylation therapy | INH, 20, 40 and 80 ppm × 2 h, sequential increasing dose regimen | NCT04528771 (US) | S-nitrosylation therapy, improve oxygen delivery of banked blood. | - | |
| | Prolectin-M | Immunomodulator | - | NCT04512027 (IN) | (1-6)-alpha-D-Mannopyranose class, decrease viral load | - | |
| | Measles-Mumps-Rubella Vaccine | Immunomodulator | IM, 0.5 mL | NCT04357028 (EG) NCT04475081 (US) | Boost a immune response, reduce levels of SARS-CoV-2, and lessen the symptoms associated with COVID-19. | Measles-Mumps-Rubella Vaccine | |
| | MenACWY | Immunomodulator | IM | NCT04324606 (GB) NCT04400838 (GB) | Boost a immune response, reduce levels of SARS-CoV-2, and lessen the symptoms associated with COVID-19. | MenACWY vaccine protects against 4 different strains of the meningococcal bacteria that cause meningitis and blood poisoning (septicaemia) | |
| | Mycobacterium w | Immunomodulator | SC, 0.3 mL, 3 d | NCT04358809 (IN) | Autoclaved Mycobacterium w, acts through the toll-like receptors (TLRs) pathway and enhances the host-T cell responses. | Immunomodulator for leprosy | |
| | oral polio vaccine | Immunomodulator | PO, 0.1 mL | NCT04445428 (GN) | Boost a immune response, reduce levels of SARS- | - | |

© 2020. Peng Y et al. Published online at http://www.aginganddisease.org/EN/10.14336/AD.2020.1124

| | | | | | | | |
|---|---|---|---|---|---|---|---|
| | | | | | CoV-2, and lessen the symptoms associated with COVID-19. | | |
| | RUTI® vaccine | Immunomodulator | IM, 25 μg | NCT04453488 (ES) | fragmented, purified and liposomed heat-inactivated Mycobacterium tuberculosis bacilli. | Therapeutic vaccine for tuberculosis. | |
| | Triiodothyronine-3 | Hormone | IV, 0.8 μg/kg, < 30 d | NCT04348513 (GR) | Regulate stress via inducing p38 MAPK activation, exert antiapoptotic action and protects tissue from injury | Treatment of depressive disorders | |
| | Prolectin-M | Immunomodulator | - | NCT04512027 (IN) | (1-6)-alpha-D-Mannopyranose class, decrease viral load | - | |
| | AT-001 | Aldose reductase inhibitor | PO, 1500 mg, bid, 14 d | NCT04365699 (US) | 1. Prevent oxidative damage to cardiomyocytes; 2. Decrease oxidative-induced damage. COVID-19 can cause significant cardiac morbidities, including cardiomyopathy. | Investigational drug for diabetic cardiomyopathy | |
| | Amiodarone | Ion channel blocker | INH, 150 mg in 10 min, infusion of 1 mg/min for 6 h, next infusion of 0.5 mg/min for 18 h, and 200-400 mg/day PO. | NCT04351763 (PL) | Intravenous amiodarone to mitigate the risk of sudden cardiac death in COVID19 patients | Treatment of ventricular and supraventricular arrhythmias | [170] |
| | Verapamil | Non–dihydropyridine calcium channel blocker | IV, 0.075-0.15 mg/kg | NCT04351763 (PL) | Management of hypertension in COVID-19 | Treatment of high blood pressure and angina | |
| | DUR-928 | Hepatoprotective drug | IV, 90 or 150 mg | NCT04447404 (US) | A sulfated oxysterol, plays an important regulatory role in cellular functions such as lipid homeostasis, inflammation, and cell survival. | Investigational drug for nonalcoholic steatohepatitis and alcoholic hepatitis | |
| | Atorvastatin | Statin medication | PO, 40 mg | NCT04380402 (US) NCT04466241 (FR) NCT04486508 (IR) | Statins affect endothelial dysfunction and have anti-inflammatory and immunomodulatory effects. | Prevention medicine of cardiovascular disease | |
| | Bucillamine | Thiol antioxidant | PO, 100 or 200 mg, tid | NCT04504734 (CA) | Attenuate clinical symptoms in respiratory viral infections in animals and humans, primarily via donation of thiols to increase antioxidant activity of cellular glutathione | Treatment of rheumatism | |

PO: Oral administration; IV: Intravenous administration; INH: Inhalation; IM: intramuscular administration; SC: Subcutaneous administration; ISIN: Intrasinal administration; ID: intradermal injection; SL: Sublingual administration; TOPp: patch applied on the skin; ET: Endotracheal instillation

© 2020. Peng Y et al. Published online at http://www.aginganddisease.org/EN/10.14336/AD.2020.1124
